# Neddylation Targets and Stabilizes NLRP3 to Augment Inflammasome‐Mediated Colitis and Mood Disorder

**DOI:** 10.1002/advs.202505906

**Published:** 2026-01-09

**Authors:** Wenbin Gai, Mengyao Wu, Anbiao Wu, Zhaofei Jing, Zhenjie Ye, Jiayan Jin, Yaolin Zhang, Min Zhao, Genyu Liu, Xu Wang, Xiqin Yang, Jie Dong, Yunlu Xu, Jiyan Zhang

**Affiliations:** ^1^ Beijing Institute of Basic Medical Sciences Beijing China; ^2^ School of Basic Medical Sciences Anhui Medical University Hefei China; ^3^ Laboratory of Snake Venom Fujian Medical University Fuzhou China; ^4^ Hengyang Medical School University of South China Hengyang China; ^5^ Chinese Institute for Brain Research Beijing China

**Keywords:** colitis, inflammasome, mood disorder, neddylation, NLRP3, ubiquitination

## Abstract

The activation of NLRP3 inflammasome contributes to the development of numerous chronic inflammatory diseases, including ulcerative colitis and stress‐induced anxiety. Recent studies have revealed that NLRP3 K48‐linked ubiquitination by several E3 ligases, including Trim31, leads to degradation. Neddylation is a process highly similar to ubiquitination by covalently conjugating NEDD8 to lysines in specific substrate proteins. Neddylation of substrate proteins alters their subcellular localization, stability, and activity. The role of neddylation in the NLRP3 inflammasome remains elusive. Here, we report that neddylation promotes the activation of the NLRP3 inflammasome in macrophages. Myeloid deficiency of UBA3, the catalytic subunit of the NEDD8‐activating enzyme (NAE), renders mice resistant to dextran sodium sulfate‐induced colitis. Inducible *Uba3* deletion in microglia mitigates psychological stress‐induced anxiety‐like behavior. Neddylation blockade led to a reduced protein level of NLRP3 without affecting its mRNA level. Further exploration revealed that NLRP3 undergoes neddylation at K287 with Ube2M as the E2 and Smurf2 as an E3, respectively. NLRP3 neddylation hinders its interaction with Trim31 and thereby inhibits its K48‐linked ubiquitination and subsequent degradation. MLN4924, a potent compound NAE inhibitor in phase 1/2/3 clinical trials for cancers, alleviates psychological stress‐induced NLRP3 inflammasome activation, microglia inflammatory activation, and anxiety‐like behavior, suggesting novel clinical activity of MLN4924.

## Graphical abstract

NLRP3 inflammasome contributes to colitis and mood disorder. This study demonstrates that neddylation targets NLRP3 at K287, which hinders its interaction with K48‐linked ubiquitination E3 Trim31 and thereby stabilizes it. Neddylation blockade in myeloid cells and microglia mitigates DSS‐induced colitis and psychological stress‐induced anxiety‐like behavior, respectively. Neddylation inhibitor MLN4924 also alleviates psychological stress‐induced mood disorder, suggesting potential clinical application.

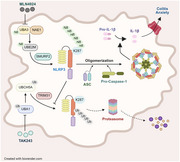

## 1 Introduction

The nucleotide‐binding domain (NOD)‐, leucine‐rich repeat (LRR)‐, and pyrin domain (PYD)‐containing protein 3 (NLRP3) inflammasome, which primarily functions in macrophages, is a critical component of the innate immune system [1, 2, 3]. Activation of nuclear factor‐κB (NF‐κB) mediates the transcription of NLRP3 and pro‐IL‐1β, which is often achieved by lipopolysaccharide (LPS). NLRP3 undergoes self‐oligomerization through its NOD, whereas its PYD mediates homotypic interactions with the adaptor protein apoptosis‐associated speck‐like protein containing a caspase recruitment domain (ASC) [1, 2, 3]. Then, various stimuli such as adenosine triphosphate (ATP) [4], monosodium urate (MSU) [5], and cholesterol crystals [6] trigger the assembly of the complex containing NLRP3 and ASC, which in turn recruits and activates pro‐Caspase‐1. Dimerization of pro‐Caspase‐1 is believed to facilitate its autoproteolytic processing [7], leading to the cleavage of pro‐IL‐1β and gasdermin D (GSDMD) [8]. The limited proteolysis of GSDMD generates an N‐terminal fragment to form large oligomeric membrane pores to release IL‐1β [8]. The activation of NLRP3 inflammasome contributes to the development of numerous chronic inflammatory diseases, including ulcerative colitis [9, 10] and stress‐induced anxiety [11, 12].

The generation of reactive oxygen species (ROS) is implicated in NLRP3 inflammasome assembly and activation [13]. ROS activates c‐Jun N‐terminal kinase (JNK), which depends on its phosphorylation at Thr183/Tyr185 (P‐JNK) [14]. JNK1‐mediated NLRP3 phosphorylation is essential for NLRP3 inflammasome activation [15]. Besides phosphorylation, NLRP3 can undergo different forms of ubiquitination [9, 16, 17, 18, 19, 20]. For example, Trim31 catalyzes K48‐linked NLRP3 ubiquitination and the consequent proteasomal degradation, whereas Cullin1 promotes K63‐linked NLRP3 ubiquitination and thereby represses NLRP3 function without affecting its degradation [17, 20]. The regulation of NLRP3 ubiquitination remains the focus of this field.

Neddylation is a process highly similar to ubiquitination. Mature ubiquitin (Ub)‐like protein neural precursor cell expressed developmentally downregulated protein 8 (NEDD8) is activated by the NEDD8‐activating enzyme E1 (NAE), a heterodimer of the scaffold amyloid precursor protein binding protein and the catalytic subunit Ub‐like modifier activating enzyme 3 (UBA3) [21]. Subsequently, NEDD8 is transferred to the NEDD8‐conjugating enzyme E2 (Ube2M or Ube2F) through a trans‐thiolation reaction [22]. Finally, NEDD8 is conjugated to the lysine residue of the substrate protein by a substrate‐specific NEDD8 E3 ligase [23]. Neddylation of Cullins activates Cullin‐RING ligases, the largest family of E3 ligases, which contributes to phosphorylation‐dependent ubiquitination and degradation of IκBα and thereby promotes NF‐κB activation [24]. On the other hand, neddylation of other substrates alters their subcellular localization, stability, and activity [23]. A previous study reported the interaction of endogenous NEDD8 with endogenous Caspase‐1 in inflammasome‐activated macrophages. Autocatalytic activity of pro‐Caspase‐1 was augmented after NEDD8 overexpression but blocked by MLN4924, an inhibitor of NAE [25]. However, the strategy of substrate identification failed to meet the criteria for a genuine neddylation target [23], and an in vivo role of neddylation in LPS + ATP‐induced IL‐1β secretion was only tested with MLN4924 [25]. The role of neddylation in the NLRP3 inflammasome remains elusive.

Here, we report that neddylation targets and stabilizes NLRP3 and thereby contributes to dextran sodium sulfate (DSS)‐induced colitis and psychological stress‐induced anxiety.

## 2 Results

### 2.1 Neddylation Promotes the Activation of NLRP3 Inflammasome in Macrophages

**FIGURE 1:**
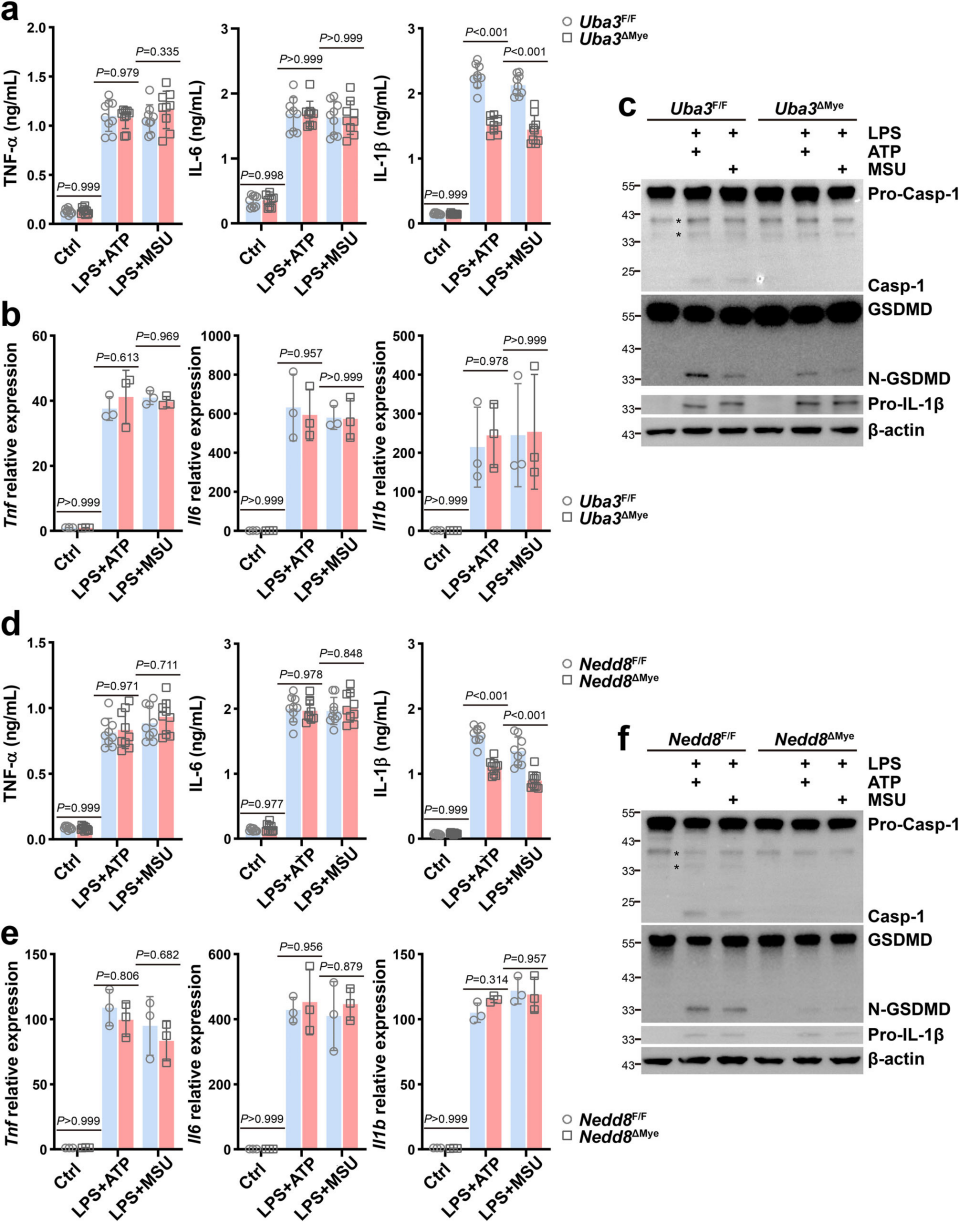
Neddylation promotes the activation of NLRP3 inflammasome in macrophages. BMDMs from *Uba3* ^ΔMye^ (a–c) or *Nedd8* ^ΔMye^ (d–f) mice and their corresponding littermates were primed with 100 ng/mL LPS for 4 h, followed by treatment with 5 mm ATP for 30 min (LPS +ATP) or 200 ng/mL MSU for 6 h (LPS +MSU). (a,d) Supernatants were harvested and subjected to ELISA to detect TNF‐α, IL‐6, and IL‐1β levels (*n* = 9/group). (b,e) Total RNA was extracted and subjected to quantitative RT‐PCR of *Tnf*, *Il6*, and *Il1b* (*n* = 3/group). (c,f) Whole cell lysates were harvested and subjected to IB to detect inflammasome activation. Error bars show mean ± SD. *P* values were determined by two‐way ANOVA. ^*^, nonspecific band; Casp‐1, Caspase‐1. All data in this figure are representative of three independent experiments.

We previously showed that myeloid neddylation promotes anti‐viral innate immunity without hindering the development and survival of macrophages under steady state [26, 27]. To explore a possible role of neddylation in the activation of NLRP3 inflammasome, bone marrow‐derived macrophages (BMDMs) from *Uba3* ^F/F^ and *Uba3* ^ΔMye^ mice were primed with LPS, followed by stimulation with ATP (LPS +ATP) or MSU (LPS +MSU). ELISA revealed comparable levels of TNF‐α and IL‐6 in the supernatants of UBA3‐sufficient and ‐deficient BMDMs (Figure 1a). However, UBA3‐deficient BMDMs secreted a lower level of IL‐1β than their UBA3‐sufficient counterparts (Figure 1a). It is possible that the reduced secretion of IL‐1β results from impaired NF‐κB activation. In this scenario, we first analyzed IκBα degradation. Immunoblotting (IB) analysis revealed that LPS‐induced IκBα degradation was only partially abrogated in the absence of UBA3 (Figure S1a). Then we performed a microarray‐based transcriptome analysis of BMDMs from *Uba3* ^F/F^ and *Uba3* ^ΔMye^ mice before and after LPS priming. As expected, LPS significantly up‐regulated mRNA levels of various NF‐κB target genes such as *Tnf*, *Il6*, *Il1a*, *Il1b*, and *Ccl2*. Intriguingly, the up‐regulation of most NF‐κB target genes in UBA3‐deficient BMDMs was similar to that in UBA3‐sufficient BMDMs (Figure S1b). Indeed, gene set enrichment analysis indicated no enrichment of NF‐κB target genes between the two groups (Figure S1c). Quantitative RT‐PCR confirmed the comparable induction of *Tnf*, *Il6*, and *Il1b* by LPS + ATP or LPS + MSU in the presence or absence of UBA3 (Figure 1b). These data rule out the possibility that the reduced secretion of IL‐1β results from impaired transcription, suggesting that neddylation promotes the activation of the NLRP3 inflammasome. In line with this notion, IB revealed that NLRP3 inflammasome agonists ATP and MSU‐triggered generation of active Caspase‐1 and N‐terminal fragment of GSDMD (N‐GSDMD) diminished in the absence of UBA3, even though protein levels of pro‐Caspase‐1, GSDMD, and pro‐IL‐1β were not significantly lower (Figure 1c).

To confirm the role of neddylation in the activation of NLRP3 inflammasome, we also cultured BMDMs from *Nedd8* ^F/F^ and *Nedd8* ^ΔMye^ mice (Figure S2). Consistent with the data shown in Figure 1a, ELISA revealed that NEDD8‐deficient BMDMs secreted similar levels of TNF‐α and IL‐6, but a lower level of IL‐1β, after LPS + ATP or LPS + MSU treatment, as compared to their NEDD8‐sufficient counterparts (Figure 1d). Quantitative RT‐PCR indicated comparable up‐regulation of *Tnf*, *Il6*, and *Il1b* by LPS + ATP or LPS + MSU in NEDD8‐sufficient and ‐deficient BMDMs (Figure 1e). ATP and MSU‐triggered generation of active Caspase‐1, N‐GSDMD, and mature IL‐1β diminished in the absence of NEDD8, even though protein levels of pro‐Caspase‐1, GSDMD, and pro‐IL‐1β were not significantly lower (Figure 1f). Thus, neddylation promotes the activation of the NLRP3 inflammasome.

### 2.2 Myeloid Neddylation Blockade Renders Mice Resistant to DSS‐Induced Colitis

**FIGURE 2:**
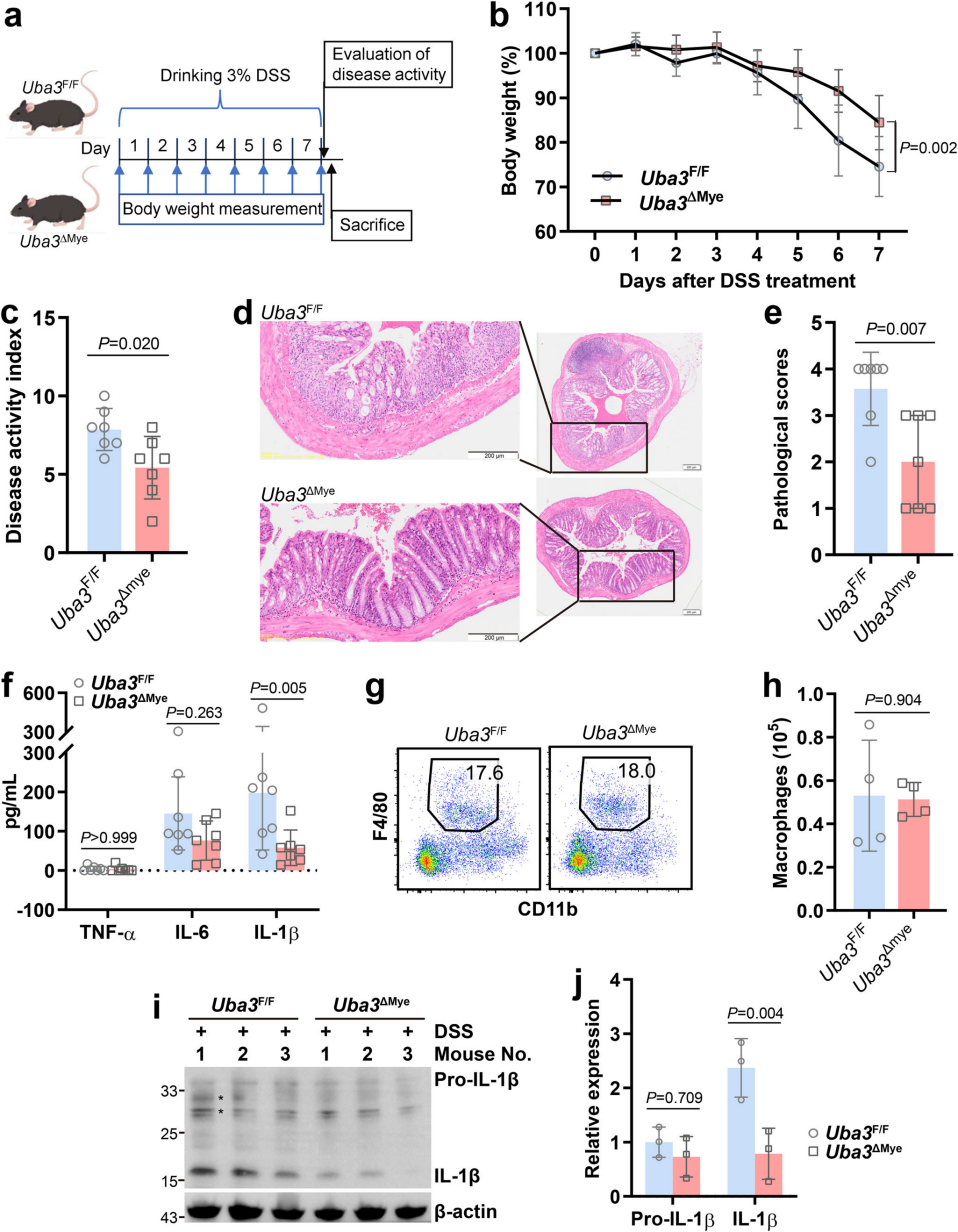
Myeloid neddylation blockade renders mice resistant to DSS‐induced colitis. (a–f) *Uba3* ^F/F^ and *Uba3* ^ΔMye^ mice (*n* = 7/group) were treated with 3% DSS for 7 consecutive days. Body weight was monitored daily. After the disease activity index was calculated, all mice were sacrificed, and the colon tissues were harvested. The experiment schedule (a), body weight changes (b), disease activity index (c), representative images of H & E staining (d, scale bar: 200 µm), pathological scores according to the histology (e), and cytokine levels analyzed by ELISA (f) are shown. (g,h) Flow cytometry analysis of macrophages infiltrating the colon (*n* = 4/group) after 4 days of 3% DSS treatment, gating live single CD45^+^ cells. Representative plots (g) and the number of macrophages (h) are shown. (i,j) IB analysis of pro‐IL‐1β and IL‐1β in the colon after 4 days of 3% DSS treatment (i, *n* = 3/group) and the corresponding densitometric readings (j). Error bars show mean ± SD. *P* values were determined by two‐tailed Student's *t* test (c,e,h) or two‐way ANOVA (b,f,j). ^*^, nonspecific band. All data in this figure are representative of two independent experiments.

The pathogenesis of ulcerative colitis has been demonstrated to be closely related to the activation of the NLRP3 inflammasome [9, 10]. Since our aforementioned data indicate that neddylation promotes the activation of NLRP3 inflammasome, it is of interest to evaluate how neddylation blockade in myeloid cells might affect the pathogenesis of ulcerative colitis. For this purpose, *Uba3* ^ΔMye^ mice and their control littermates were fed with 3% DSS solution for 7 consecutive days (Figure 2a). Compared with control littermates, *Uba3* ^ΔMye^ mice experienced less bodyweight loss (Figure 2b). Synchronously, *Uba3* ^ΔMye^ mice had lower disease activity index scores (Figure 2c), which is a vital parameter reflecting the severity of colitis. Furthermore, histopathological examination of colon sections of *Uba3* ^F/F^ mice showed severe epithelial destruction, areas of mucosal ulceration, and loss of goblet cells, as well as increased numbers of mucosal and submucosal infiltrating leukocytes, which were much alleviated in colon sections of *Uba3* ^ΔMye^ mice (Figure 2d). Consequently, *Uba3* ^ΔMye^ colons exhibited lower histological scores than *Uba3* ^F/F^ colons (Figure 2e). Furthermore, ELISA of the colon tissue revealed a lower level of IL‐1β in *Uba3* ^ΔMye^ colons than that in *Uba3* ^F/F^ colons, while there was no significant difference in TNF‐α and IL‐6 levels between the two groups (Figure 2f).

To clarify whether the above effects of myeloid UBA3 deficiency result from a reduced number of macrophages infiltrating the colon, we isolated immune cells in the colon. Flow cytometry analysis revealed a similar number of macrophages in the colon of *Uba3* ^F/F^ and *Uba3* ^ΔMye^ mice after 4 days of 3% DSS treatment (Figure 2g,h). At this time point, IB of the colon tissue revealed a reduced level of mature IL‐1β in the absence of myeloid UBA3, even though that of pro‐IL‐1β was largely unchanged (Figure 2i,j). Therefore, myeloid neddylation is required for activating NLRP3‐dependent ulcerative colitis.

### 2.3 Inducible Neddylation Blockade in Microglia Mitigates Psychological Stress‐Induced Anxiety‐Like Behavior

**FIGURE 3:**
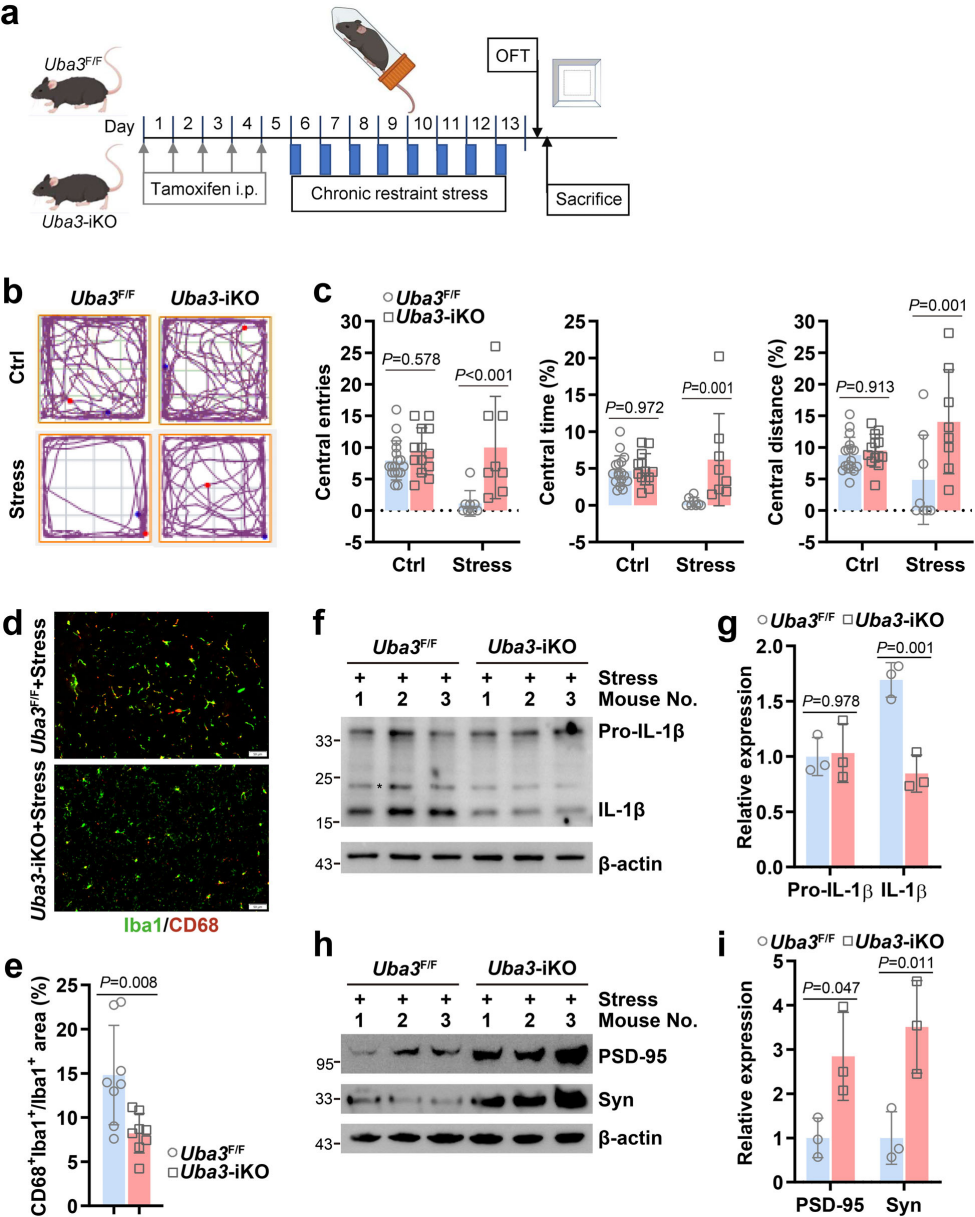
Inducible neddylation blockade in microglia mitigates psychological stress‐induced anxiety‐like behavior. *Uba3* ^F/F^ and *Uba3*‐iKO mice were subjected to restraint from 0:00 to 8:00 a.m. each day for 8 consecutive days (stress group) or placed in the home cage at the same time without food and water (control group), followed by the open field test (OFT). Then stressed mice were sacrificed. Amygdala coronal sections were prepared, or amygdala tissues were lysed in RIPA buffer. (a) The experiment schedule. (b,c) Representative tracks (b) and statistical data (c, *n* = 8–17/group) of OFT. (d,e) Immunofluorescence staining of Iba1 and CD68 in amygdala coronal sections of stressed mice (*n* = 8/group). Representative images (d, scale bar: 50 µm) and statistical data of Iba1 and CD68 co‐labeling (e) are shown. (f–i) IB analysis of IL‐1β maturation (f) and synaptic loss (h) in the amygdala of stressed mice (*n* = 3/group) and the corresponding densitometric readings (g,i). ^*^, nonspecific band; Syn, Synaptophysin. Error bars show mean ± SD. *P* values were determined by two‐tailed Student's *t* test (e) or two‐way ANOVA (c,g,i). All data in this figure are representative of two independent experiments.

Anxiety is one of the most prevalent psychiatric disorders with synaptic dysfunction [28, 29]. Since the NLRP3 inflammasome has been revealed to mediate psychological stress‐induced anxiety‐like behaviors through making a feed‐forward circle with microglia inflammatory activation [11, 12], we set out to explore whether neddylation is implicated in this process. For this purpose, *Uba3* ^F/F^ mice were crossed with *Cx3cr1‐CreERT2* mice, a model widely used for tamoxifen‐inducible Cre‐mediated recombination in microglia [30], to generate *Uba3* ^F/F; Cx3cr1‐CreERT2^ (*Uba3‐*iKO) mice. After tamoxifen injection, male *Uba3‐*iKO mice and their control littermates were subjected to an 8‐day‐restraint stress model (Figure 3a). Mice experience no physical injury during restraint, and therefore only undergo psychological stress [11, 31]. As expected, IB indicated that restraint stress led to augmented levels of P‐JNK, NLRP3, and mature IL‐1β in the amygdala (Figure S3a), a brain region critical for the generation of persistent anxiety [31, 32, 33]. In line with the previous findings that IL‐1β induces synaptic loss [34, 35], IB also revealed reduced levels of pre‐synaptic protein Synaptophysin [36] and post‐synaptic marker PSD‐95 [37] in this brain region of stressed mice (Figure S3b). Thus, this model is suitable to study the psychological stress→NLRP3 inflammasome↔microglia inflammatory activation,→ synaptic loss, →anxiety‐like behavior axis.

Without restraint treatment, *Uba3‐*iKO mice and their littermates showed similar activities in the open field test (OFT, Figure 3b,c). Restraint stress led to fewer entries into the central area and less time and distance traveled there in *Uba3* ^F/F^ mice (Figure 3b,c). However, *Uba3‐*iKO mice, which underwent restraint stress, exhibited more central area entries and more time and distance traveled there (Figure 3b,c), indicating mitigated anxiety‐like behavior. Then we tried to analyze microglia inflammatory activation in the amygdala with indirect immunofluorescence staining. For this method, ionized calcium binding adaptor molecule 1 (Iba1) is a widely used marker for detecting microglia, whereas macrophage M1 polarization marker CD68 is widely used to reflect microglia inflammatory activation [11, 12]. In line with their mitigated anxiety‐like behavior, stressed *Uba3‐*iKO mice displayed reduced co‐labeling of Iba1 and CD68 in the amygdala, as compared to their littermates (Figure 3d,e). Furthermore, IB of the amygdala tissue revealed a reduced level of mature IL‐1β in the absence of microglial UBA3, even though that of pro‐IL‐1β was largely unchanged (Figure 3f,g). Accordingly, protein levels of Synaptophysin and PSD‐95 were augmented in the absence of microglial UBA3 (Figure 3h,i), suggesting alleviated synaptic loss in the amygdala.

### 2.4 Neddylation Maintains the Protein Level of NLRP3

**FIGURE 4:**
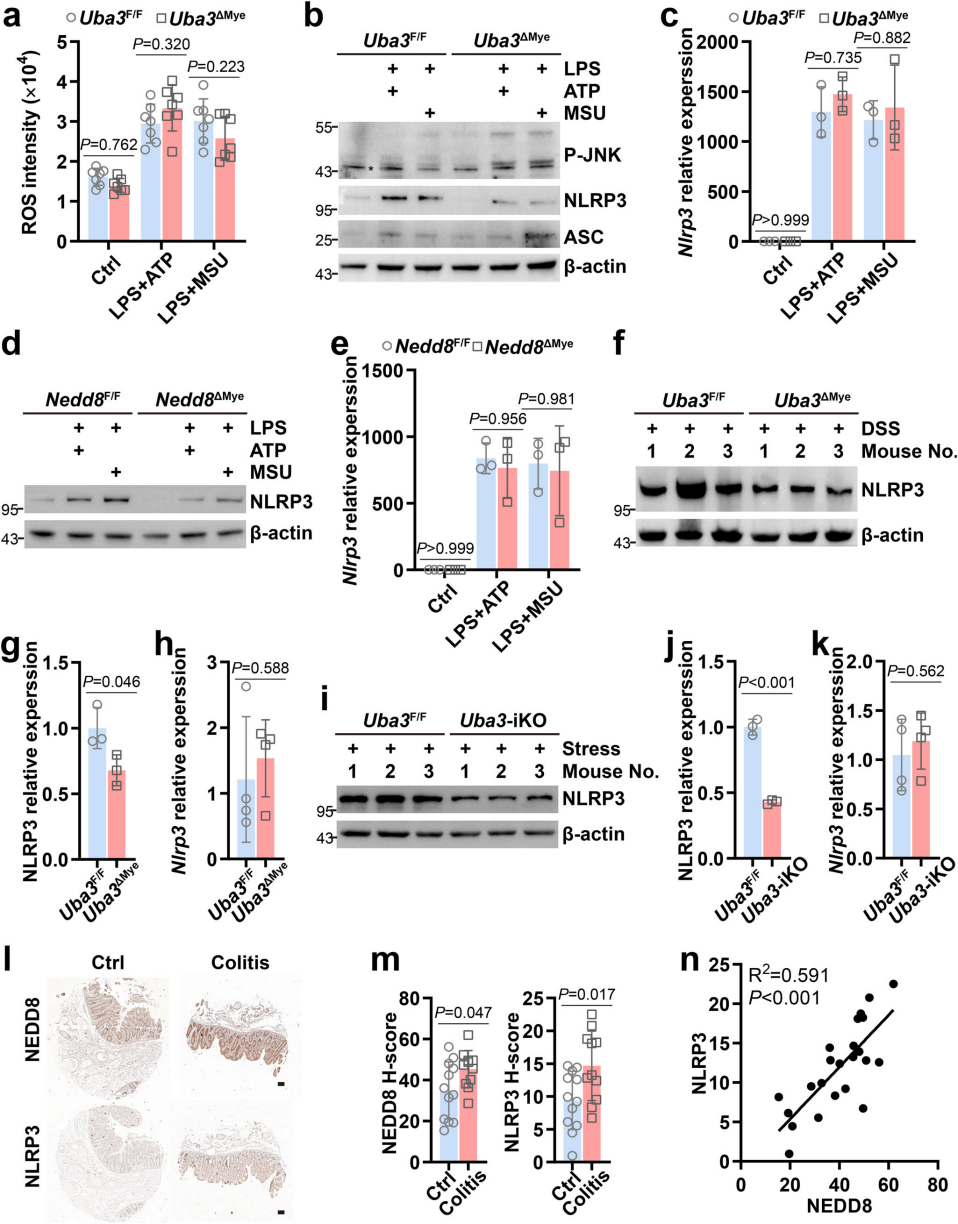
Neddylation maintains the protein level of NLRP3. (a–c) BMDMs from *Uba3* ^F/F^ and *Uba3* ^ΔMye^ mice were treated as described in Figure 1a, followed by ROS measurement (a, *n* = 7/group), IB analysis of upstream components of NLRP3 inflammasome (b), and quantitative RT‐PCR of *Nlrp3* (c, *n* = 3/group). (d,e) BMDMs from *Nedd8* ^F/F^ and *Nedd8* ^ΔMye^ mice were treated as stated in Figure 1a, followed by IB of NLRP3 (d) and quantitative RT‐PCR of *Nlrp3* (e, *n* = 3/group). (f–k) The indicated genotypes of mice were treated with 3% DSS for 4 consecutive days (f–h) or with restraint for 8 consecutive days (i–k). Colon tissues (f–h) or amygdala tissues (i–k) were harvested, respectively, followed by IB analysis of NLRP3 (f,i, *n* = 3/group), densitometric readings of IB blots (g,j), and quantitative RT‐PCR of *Nlrp3* (h,k, *n* = 4/group). (l–n) Clinical non‐colitis control colon tissues (*n* = 12) and colon tissues with chronic colitis (*n* = 11) were examined for NEDD8 and NLRP3 staining on tissue microarray slides. Representative immunohistochemistry images of paired samples (l, scale bar: 100 µm), comparison of NEDD8 and NLRP3 staining levels between the two groups (m), and Spearman's rank correlation of NEDD8 and NLRP3 staining levels (n) are shown. Error bars show mean ± SD. *P* values were determined by two‐tailed Student's *t* test (g,h,j,k,m) or two‐way ANOVA (a,c,e). ^*^, nonspecific band; P‐JNK, phospho‐JNK at Thr183/Tyr185. Data in (a–k) are representative of two independent experiments.

Next, we tried to analyze the mechanisms by which neddylation promotes the activation of the NLRP3 inflammasome. Because the generation of ROS is a key upstream event of NLRP3 inflammasome assembly and activation [13], we first checked ROS generation triggered by NLRP3 inflammasome agonists ATP and MSU. BMDMs from *Uba3* ^F/F^ and *Uba3* ^ΔMye^ mice were primed with LPS, followed by stimulation with ATP or MSU. The usage of the fluorescent probe 2’,7’‐dichlorodihydrofluorescein diacetate (DCFH‐DA) indicated similar ROS levels in UBA3‐sufficient and ‐deficient BMDMs before and after treatment with NLRP3 inflammasome agonists (Figure 4a). ROS activates JNK [14]. In line with our previous findings that neddylation of JNK upstream kinase mitogen‐activated protein kinase kinase 7 limits its activity [38], IB revealed enhanced P‐JNK in the absence of UBA3 (Figure 4b). JNK1 phosphorylates NLRP3, which is essential for NLRP3 inflammasome activation [15]. In our efforts to measure the phosphorylation of NLRP3 in the absence of UBA3, we unexpectedly found that the protein level of NLRP3, but not that of ASC, was reduced in UBA3‐deficient BMDMs, either before or after LPS priming (Figure 4b). Yet quantitative RT‐PCR indicated similar induction of *Nlrp3* by LPS + ATP or LPS + MSU in the presence or absence of UBA3 (Figure 4c). In this scenario, we re‐examined our microarray‐based transcriptome data for the mRNA level of NLRP3. As shown in Figure S1b, UBA3 showed no effect on the basal *Nlrp3* level and LPS‐induced up‐regulation. These data suggest that the reduction of NLRP3 protein level in UBA3‐deficient BMDMs occurs at the post‐transcriptional level. Reduced NLRP3 protein level but unchanged *Nlrp3* mRNA level before and after LPS priming were also observed in BMDMs from *Nedd8* ^ΔMye^ mice (Figure 4d,e). Importantly, colon tissues from *Uba3* ^ΔMye^ mice underwent DSS treatment (Figure 4f–h) and amygdala tissues from *Uba3‐*iKO mice underwent restraint stress (Figure 4i–k) exhibited lower levels of NLRP3 but unchanged levels of *Nlrp3*, as compared to their counterparts from *Uba3* ^F/F^ mice. Accordingly, immunohistochemistry revealed that, compared with non‐colitis control colon tissues, clinical colon tissues with chronic colitis presented enhanced staining of both NEDD8 and NLRP3 (Figure 4l,m). Notably, the level of NEDD8 was positively associated with that of NLRP3 (Figure 4l,n), suggesting the maintenance of NLRP3 protein level by neddylation is of clinical relevance.

### 2.5 NLRP3 is a Neddylation Substrate

**FIGURE 5:**
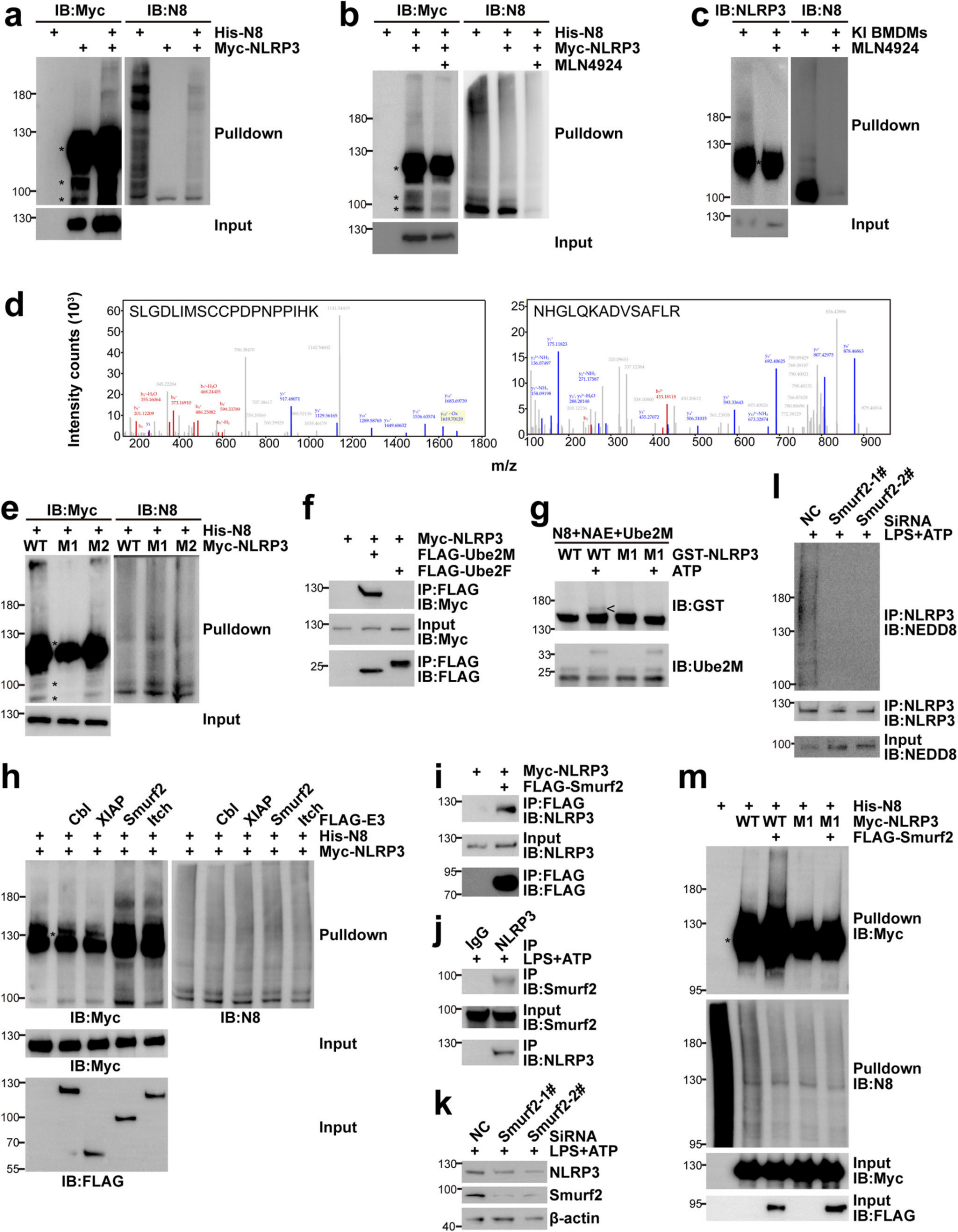
NLRP3 is a neddylation substrate. (a,b,e,h,m) Twenty‐four hours after transfection with the indicated plasmids, HEK‐293T cells were directly subjected to histidine pulldown under fully denaturing conditions to examine Myc‐NLRP3 neddylation (a,e,h,m) or subjected to histidine pulldown after treatment with 0.2 µm MLN4924 or DMSO of equal volume for 12 h (b). (c) After pre‐treatment with 0.2 µm MLN4924 or DMSO of equal volume for 2 h, BMDMs from 6 × His‐FLAG‐NEDD8 heterozygous KI mice were primed with 100 ng/mL LPS for 4 h, followed by treatment with 5 mm ATP for 30 min. NLRP3 neddylation was examined by histidine pulldown. (d) The smear band described in Figure S6 was subjected to mass spectrometry analysis. Possibly neddylated peptides of NLRP3 are shown. (f,i) Twenty‐four hours after HEK‐293T cells were transfected with plasmids encoding Myc‐NLRP3 and FLAG‐tagged Ube2M (f), Ube2F (f), or Smurf2 (i), the potential interaction between NLRP3 and the indicated E2/E3 was examined by IB after IP with an antibody against FLAG‐tag. (g) Bacterially expressed GST‐tagged NLRP3 WT or K287R was incubated with the indicated purified proteins at 37°C in the absence or presence of ATP for 1 h. The samples were then subjected to IB with antibodies against GST and Ube2M. Neddylated GST‐NLRP3 is indicated with the symbol‘<‘. (j–l) BMDMs from wild‐type mice were transfected with Smurf2 siRNA or non‐targeting control (NC) siRNA or left untreated, followed by LPS + ATP stimulation. The interaction between endogenous Smurf2 and endogenous NLRP3 was analyzed by IB after IP with an antibody against NLRP3 or control rabbit IgG (j). The protein level of NLRP3 was analyzed by IB (k). NLRP3 neddylation was examined by IB after IP with an antibody against NLRP3 or control rabbit IgG under partially denaturing conditions (l). Please note that in (b,c,e,f,h,i,l,m), the amount of lysates (c,l) or Myc‐NLRP3 plasmid (b,e,f,h,i,m) in each sample was adjusted to ensure a comparable protein level of NLRP3 between different samples. ^*^, nonspecific band; N8, NEDD8; WT, wild type; M1, K287R; M2, K494R. The data in this figure are representative of three independent experiments except (d).

To investigate whether NLRP3 is a novel neddylation substrate, we first co‐transfected plasmids encoding His‐NEDD8 and Myc‐NLRP3 in HEK‐293T cells. Histidine pulldown under fully denaturing conditions clearly demonstrated that His‐NEDD8 was covalently conjugated to Myc‐NLRP3 (Figure 5a). Notably, a major smear band is located at around 180 kDa, suggesting that 6–8 NEDD8 molecules can be covalently conjugated to a single NLRP3 protein. NEDD8 overexpression might lead to artificial conjugation independent of NAE [39]. To rule out this possibility, transfected HEK‐293T cells were treated with MLN4924 for 12 h. Histidine pulldown revealed that the covalent modification of Myc‐NLRP3 was reduced after MLN4924 treatment (Figure 5b). Intriguingly, the ubiquitination E1 UBA1 inhibitor TAK243 [40] exhibited opposite effects (Figure S4). Thus, the neddylation of Myc‐NLRP3 is not artificial. To confirm the covalent NEDD8 modification of endogenous NLRP3, we cultured BMDMs from 6 × His‐FLAG‐NEDD8 heterozygous knock‐in (KI) mice [41]. As expected, histidine pulldown clearly demonstrated that endogenous NLRP3 was neddylated, whereas no covalent conjugation of His‐NEDD8 to endogenous ASC, pro‐Caspase‐1, GSDMD, and pro‐IL‐1β was detected under the same conditions (Figure S5). Importantly, MLN4924 treatment abrogated the covalent His‐NEDD8 modification of endogenous NLRP3 (Figure 5c). Thus, NLRP3 is a neddylation target.

As neddylation occurs on specific lysine site(s), anti‐Myc precipitates from HEK‐293T cells co‐transfected with plasmids encoding His‐NEDD8 and Myc‐NLRP3 were obtained by IP under partially denaturing conditions. After SDS‐PAGE and silver staining (Figure S6), the major smear band at around 180 kDa was subjected to mass spectrometry analysis. As a result, lysine 287 and lysine 494 were revealed as neddylation or ubiquitination sites (Figure 5d). These two lysines were individually mutated to arginines. Histidine pulldown indicated that the neddylation of the K287R mutant, but not the K494R mutant, was abolished (Figure 5e). Therefore, NLRP3 undergoes neddylation at lysine 287.

Identification of a neddylation substrate should define the specific E2 and E3 enzymes [23]. There are two E2 enzymes for neddylation, Ube2M and Ube2F [22]. After co‐transfection in HEK‐293T cells, coimmunoprecipitation (co‐IP) revealed that Myc‐NLRP3 coprecipitated with coexpressed FLAG‐Ube2M, but not Ube2F (Figure 5f). An in vitro neddylation system can lead to E2‐dependent but E3‐independent mono‐neddylation to the substrate through generating NEDD8‐E2 thioesters [23, 26, 42, 43]. As expected, incubation of bacterially expressed GST‐tagged wild type (WT) NLRP3 with NAE, Ube2M, and NEDD8 in the presence of ATP resulted in the appearance of a slower‐migrating band about 8–10 kDa higher than free tagged NLRP3. Such a slower‐migrating band was not detected for GST‐NLRP3 K287R under the same conditions (Figure 5g). Thus, Ube2M is the neddylation E2 for NLRP3. To date, several neddylation E3 ligases have been identified, including Cbl, XIAP, Smurf2, and Itch [23, 44]. Next, we analyzed neddylation E3 ligase(s) for NLRP3. Histidine pulldown under fully denatured conditions revealed that FLAG‐Smurf2 significantly enhanced the neddylation of Myc‐NLRP3 in HEK‐293T cells, whereas FLAG‐tagged Cbl, XIAP, or Itch was less potent or showed no effect (Figure 5h). Co‐IP confirmed the interaction between Myc‐NLRP3 and FLAG‐Smurf2 in HEK‐293T cells (Figure 5i) and the interaction between endogenous NLRP3 and endogenous Smurf2 in BMDMs (Figure 5j). Accordingly, Smurf2 knockdown led to a reduced protein level of NLRP3 in BMDMs (Figure 5k). IP under partially denaturing conditions revealed that Smurf2 knockdown inhibited the neddylation of endogenous NLRP3 in BMDMs (Figure 5l). Furthermore, FLAG‐Smurf2 failed to augment the neddylation of Myc‐NLRP3 K287R under the conditions that it enhanced that of Myc‐NLRP3 WT in HEK‐293T cells (Figure 5m). Together, these data suggest that Smurf2 is a neddylation E3 for NLRP3.

### 2.6 Neddylation of NLRP3 Antagonizes Its Ubiquitination and Degradation

**FIGURE 6:**
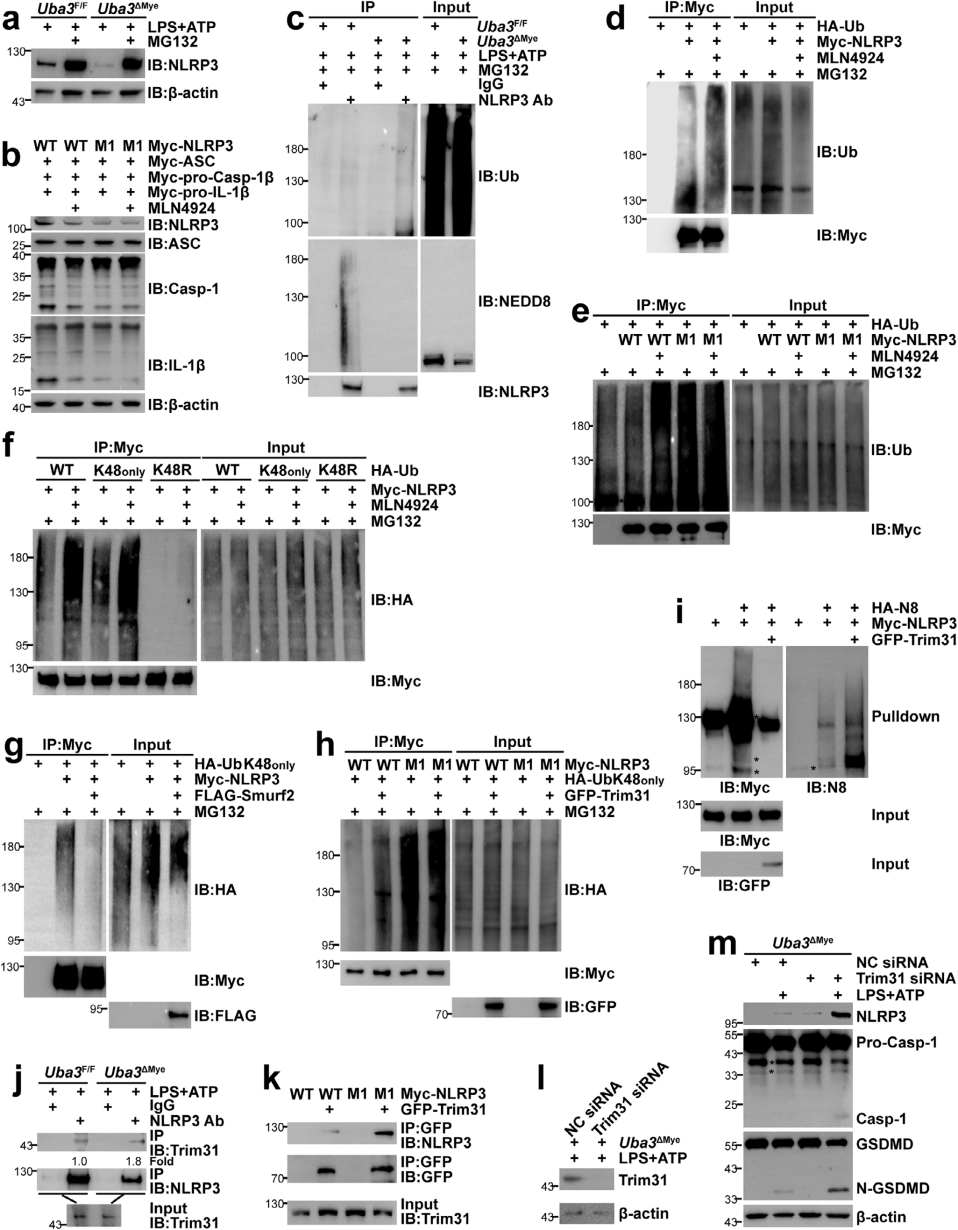
Neddylation of NLRP3 antagonizes its ubiquitination and degradation. (a,c) BMDMs from *Uba3* ^F/F^ and *Uba3* ^ΔMye^ mice were primed with 100 ng/mL LPS for 4 h in the presence or absence of 20 µm MG132, followed by treatment with 5 mm ATP for 30 min. The expression of NLRP3 and β‐actin was analyzed by IB (a). NLRP3 ubiquitination and neddylation were examined by IB after IP with an antibody against NLRP3 or control rabbit IgG under partially denaturing conditions (c). (b,d–h) Twenty‐four hours after transfection with the indicated plasmids, HEK‐293T cells were treated with or without MLN4924 (0.2 µm, 12 h) and MG132 (20 µm, 6 h) as indicated. Whole cell lysates were harvested and subjected to IB to detect inflammasome activation (b). Myc‐NLRP3 ubiquitination was examined by IB after IP with an antibody against Myc‐tag under partially denaturing conditions (d–h). (i) Twenty‐four hours after HEK‐293T cells were transfected with plasmids encoding His‐NEDD8, Myc‐NLRP3, and GFP‐Trim31, Myc‐NLRP3 neddylation was examined by histidine pulldown. (j) BMDMs from *Uba3* ^F/F^ and *Uba3* ^ΔMye^ mice were treated with LPS + ATP. The interaction between endogenous Trim31 and endogenous NLRP3 was analyzed by IB after IP with an antibody against NLRP3 or control rabbit IgG. Densitometric readings normalized with the sum of precipitated NLRP3 and input Trim31 bands. (k) Twenty‐four hours after HEK‐293T cells were transfected with Myc‐NLRP3 WT or K287R and GFP‐Trim31, the interaction between Myc‐NLRP3 and GFP‐Trim31 in was examined by IB after IP with an antibody against GFP. (l,m) BMDMs from *Uba3* ^ΔMye^ mice were transfected with trim31 siRNA or scramble control siRNA. After 48 h, BMDMs were treated with LPS + ATP. Whole cell lysates were then harvested and subjected to IB. Please note that in (i,k), the amount of Myc‐NLRP3 plasmid in each sample was adjusted to ensure a comparable protein level of NLRP3 between different samples. ^*^, nonspecific band; Ab, antibody; Ub, ubiquitin; WT, wild type; M1, K287R; N8, NEDD8; NC, non‐targeting control; Casp‐1, Caspase‐1. All data in this figure are representative of three independent experiments.

Because neddylation usually affects the stability of substrate proteins through the Ub‐proteasome system [23, 25, 41], we tested whether neddylation inhibition led to the reduction of NLRP3 protein in a proteasome‐dependent manner. Indeed, the proteasome inhibitor MG132 reversed the reduced protein level of endogenous NLRP3 in LPS + ATP‐treated UBA3‐deficient BMDMs (Figure 6a). MG132 also prevented the down‐regulation of Myc‐NLRP3 in HEK‐293T cells upon MLN4924 treatment (Figure S7a). Furthermore, the protein level of Myc‐NLRP3 K287R, the neddylation‐defective mutant, in HEK‐293T cells was always lower than that of its WT counterpart when the same amount of plasmid was used (Figure S7b). However, the difference disappeared upon MLN4924 treatment (Figure S7b). Unlike BMDMs, which require priming and triggering signals to activate the inflammasome, co‐transfection of the requisite components in HEK‐293T cells leads to spontaneous inflammasome activation [15, 20]. To facilitate the detection of active Caspase‐1 and mature IL‐1β by IB, we omitted GSDMD ectopic expression. As expected, co‐transfection of Myc‐NLRP3 WT, Myc‐ASC, Myc‐pro‐Caspase‐1β (about 40 kDa), and Myc‐pro‐IL‐1β in HEK‐293T cells resulted in the spontaneous generation of active Caspase‐1 and mature IL‐1β, which diminished upon MLN4924 treatment or NLRP3 neddylation‐defective mutation (Figure 6b). Furthermore, MLN4924 treatment failed to affect Myc‐NLRP3 K287R‐mediated Caspase‐1 activation and IL‐1β maturation (Figure 6b). Together, these data suggest that NLRP3 neddylation prevents its Ub‐proteasome–dependent degradation. IB with an antibody against Ub demonstrated no accumulation of either conjugated Ub or free Ub in UBA3‐deficient BMDMs before and after LPS + ATP or LPS + MSU treatment (Figure S8). Furthermore, IB revealed no reduction of LC3B and p53, which undergo ubiquitination and proteasome–dependent degradation [45, 46], in UBA3‐deficient BMDMs either before or after NLRP3 inflammasome activation (Figure S8). Thus, neddylation blockade does not affect the ubiquitination level globally in these settings. In this scenario, we aimed to explore how neddylation might affect NLRP3 ubiquitination. As expected, IP under partially denaturing conditions revealed that the ubiquitination of endogenous NLRP3 in BMDMs was augmented in the absence of UBA3 (Figure 6c). The ubiquitination of Myc‐NLRP3 in HEK‐293T cells was also augmented upon MLN4924 treatment (Figure 6d). Consistent with these data, the ubiquitination of neddylation‐defective Myc‐NLRP3 mutant K287R in HEK‐293T cells was more pronounced than that of Myc‐NLRP3 WT (Figure 6e). And MLN4924 failed to enhance the ubiquitination of Myc‐NLRP3 K287R as it did for Myc‐NLRP3 WT (Figure 6e).

Various types of poly‐Ub chains can be formed. Among them, K48‐linked chains target proteins for degradation by the proteasome [47]. As expected, HA‐Ub K48_only_, with lysines other than K48 mutated to arginines [47], could confer Myc‐NLRP3 ubiquitination to a similar extent to HA‐Ub WT in HEK‐293T cells either in the absence or presence of MLN4924, whereas HA‐Ub K48R‐supported Myc‐NLRP3 ubiquitination was weak and was only marginally affected by MLN4924 (Figure 6f). Thus, neddylation predominantly prevents NLRP3 K48‐linked ubiquitination, but not the conjugation of other poly‐Ub chains. All the known neddylation E3s are ubiquitination E3s [23]. It is of interest whether NLRP3 is neddylated and ubiquitinated by the same E3(s). Since FLAG‐Smurf2 significantly inhibited the K48‐linked ubiquitination of Myc‐NLRP3 in HEK‐293T cells (Figure 6g), obviously, it is not a K48‐linked ubiquitination E3 for NLRP3. Then we turned to Trim31, a reported K48‐linked ubiquitination E3 for NLRP3 in cooperation with E1 UBA1 and E2 UBCH5A [17]. As shown in Figure 6h, Myc‐NLRP3 K287R underwent augmented K48‐linked ubiquitination than Myc‐NLRP3 WT in HEK‐293T cells. GFP‐Trim31 enhanced the K48‐linked ubiquitination of Myc‐NLRP3 WT, but not that of Myc‐NLRP3 K287R. In contrast to the effects of the UBA1 inhibitor (Figure S4), GFP‐Trim31 blocked Myc‐NLRP3 neddylation in HEK‐293T cells (Figure 6i). Therefore, NLRP3 neddylation and K48‐linked ubiquitination are exerted by different E3s and antagonize each other.

It is reported that Trim31 mediates NLRP3 ubiquitination at lysine 496 [48]. Thus, NLRP3 neddylation and K48‐linked ubiquitination are not competing with each other at the same residue. How NLRP3 neddylation represses its K48‐linked ubiquitination is of interest. In vitro ubiquitination of GST‐NLRP3 with GFP‐Trim31 immunoprecipitated from HEK‐293T cells treated with or without MLN4924 indicated that neddylation blockade does not enhance the ubiquitination E3 ligase activity of Trim31 (Figure S9). In this scenario, we tried to analyze the interaction between NLRP3 and Trim31 upon neddylation blockade. Co‐IP demonstrated that the interaction between endogenous NLRP3 and endogenous Trim31 in BMDMs was augmented in the absence of UBA3 (Figure 6j). The interaction between Myc‐NLRP3 and GFP‐Trim31 in HEK‐293T cells was also augmented when its neddylation‐defective mutant K287R was used (Figure 6k). Trim31 knockdown in UBA3‐deficient BMDMs (Figure 6l) significantly reversed the reduced protein level of NLRP3 (Figure 6m), confirming its reported role as a major K48‐linked ubiquitination E3 for NLRP3 in this cell model [17, 48]. As expected, Trim31 knockdown also reversed the impaired generation of active Caspase‐1 and N‐GSDMD (Figure 6m) and the reduced secretion of IL‐1β (Figure S10). Together, these data indicate that NLRP3 neddylation represses its K48‐linked ubiquitination and subsequent degradation in macrophages via blocking the recruitment of Trim31.

### 2.7 MLN4924 Alleviates Psychological Stress‐Induced Microglia Inflammatory Activation and Anxiety‐Like Behavior

**FIGURE 7:**
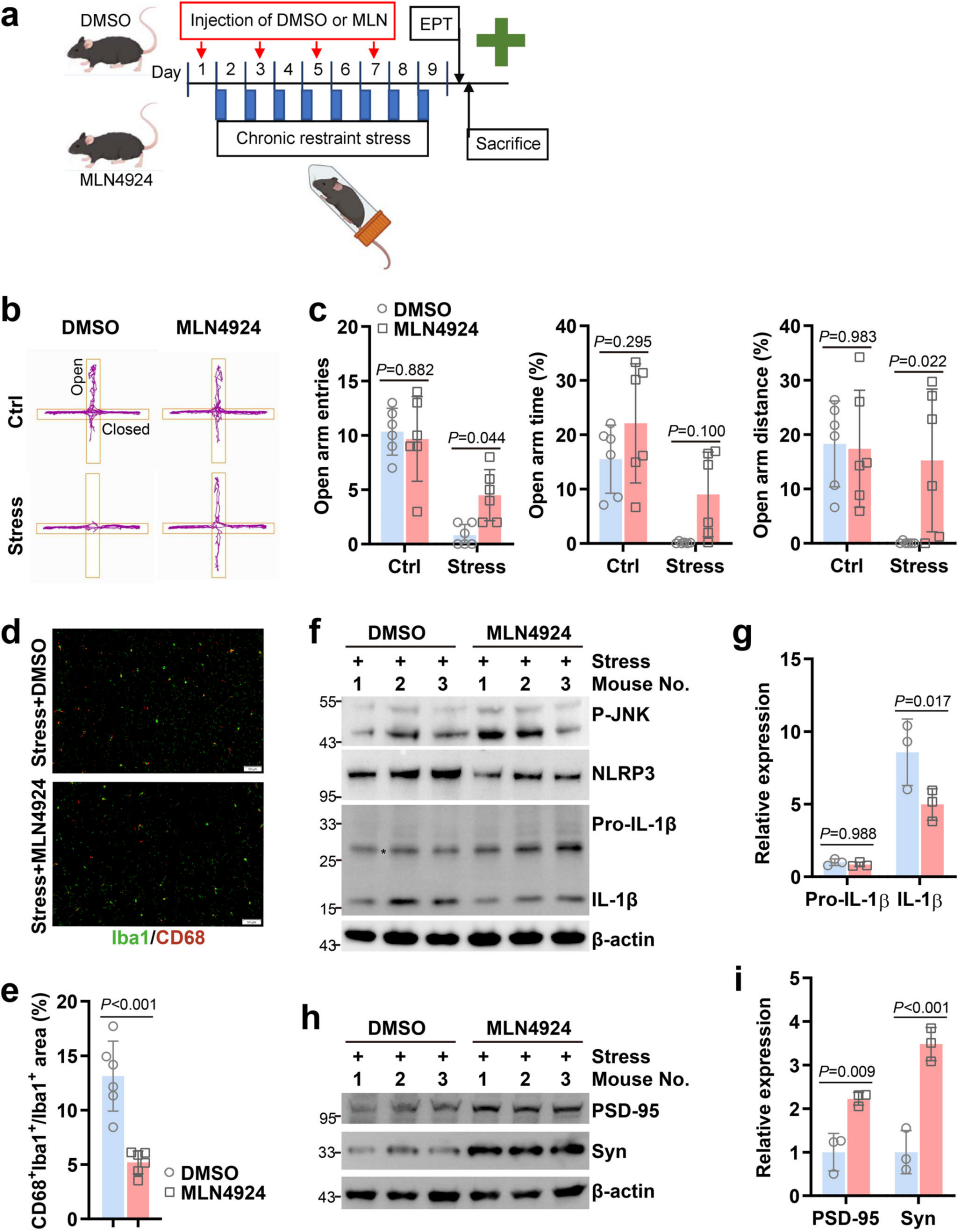
MLN4924 alleviates psychological stress‐induced microglia inflammatory activation and anxiety‐like behavior. Adult C57 BL/6 male mice were subjected to restraint from 0:00 to 8:00 a.m. each day for 8 consecutive days (stress group) or placed in the home cage at the same time without food and water (control group), followed by the elevated plus maze test (EPT). They were intraperitoneally injected with MLN4924 at a dose of 50 mg/kg or DMSO of equal volume 12 h before the 1, 3, 5, and seventh sessions of restraint. Then stressed mice were sacrificed. Amygdala coronal sections were prepared, or amygdala tissues were lysed in RIPA buffer. (a) The experiment schedule. (b,c) Representative tracks (b) and statistical data (c, *n* = 6/group) of EPT. (d,e) Immunofluorescence staining of Iba1 and CD68 in amygdala coronal sections of stressed mice (*n* = 6/group). Representative images (d, scale bar: 50 µm) and statistical data of Iba1 and CD68 co‐labeling (e) are shown. (f–i) IB analysis of inflammasome activation (f) and synaptic loss (h) in the amygdala of stressed mice (*n* = 3/group) and the corresponding densitometric readings (g,i). ^*^, nonspecific band; P‐JNK, phospho‐JNK at Thr183/Tyr185; Syn, Synaptophysin. Error bars show mean ± SD. *P* values were determined by two‐tailed Student's *t* test (c,e) or two‐way ANOVA (g,i). All data in this figure are representative of two independent experiments.

MLN4924 is in phase 1/2/3 clinical trials for cancers [49, 50, 51]. Previous studies showed that MLN4924 was protective for DSS‐induced colitis at the dose of 0.1 or 15 mg/kg/day but worsened 2,4,6‐trinitrobenzene sulfonic acid (TNBS)‐induced colitis at the dose of 3 mg/kg/day [52, 53, 54]. Despite that, the effects of MLN4924 on psychological stress‐induced mood disorders have not been reported. Possibly due to the existence of blood brain barrier, MLN4924 can only efficiently reduce ischemic brain injury in mice at the dose of 60 mg/kg, but not a lower dose [55]. In this scenario, we performed intraperitoneal administration of 50 mg/kg MLN4924 or DMSO of equal volume 12 h before the 1, 3, 5, and seventh sessions of restraint (Figure 7a). The elevated plus maze test showed that MLN4924 administration did not affect the behavior of control mice but led to more open arm entries and time and distance traveled there in mice that underwent restraint stress (Figure 7b,c), indicating alleviated anxiety‐like behavior. Next, we analyzed how MLN4924 might affect the inflammatory activation of microglia. Immunofluorescence revealed that MLN4924 administration resulted in diminished co‐labeling of Iba1 and CD68 in the amygdala of mice that underwent restraint stress (Figure 7d,e). Furthermore, IB revealed that MLN4924 administration antagonized restraint stress‐induced up‐regulation of NLRP3 and mature IL‐1β without lowering the levels of P‐JNK and pro‐IL‐1β (Figure 7f,g). Accordingly, protein levels of Synaptophysin and PSD‐95 were augmented with MLN4924 administration (Figure 7h,i), suggesting alleviated synaptic loss in the amygdala. Thus, MLN4924 alleviates psychological stress‐induced anxiety‐like behavior through limiting NLRP3 inflammasome‐mediated microglia inflammatory activation.

## 3 Discussion

Currently, the criteria for a genuine neddylation target include: (1) covalent attachment of NEDD8 through its C‐terminal glycine to a lysine residue in the target protein; (2) detectable neddylation under homeostatic conditions and at endogenous levels of NEDD8 and substrate expression; (3) neddylation of the proposed target is sensitive to MLN4924 treatment under conditions that block Cullin neddylation but not ubiquitylation, and NEDD8 is thus activated by NAE; (4) the specific neddylation E2 and E3 enzyme(s) should be defined; and (5) the regulation and biological consequences of neddylation should be disclosed [23]. We have shown the covalent conjugation of His‐NEDD8 to Myc‐NLRP3 with histidine pulldown under fully denaturing conditions. Furthermore, we have identified lysine 287 as the major neddylation site. With our 6 × His‐FLAG‐NEDD8 constitutive knock‐in mouse model, criteria (2) and (3) have been justified for NLRP3. With data demonstrating that Ube2M is the E2 and Smurf2 is an E3 for NLRP3 neddylation, criterion (4) has also been fulfilled, although more neddylation E3s may be identified for NLRP3 in the future. About the regulation, we have shown that NLRP3 neddylation at lysine 287 represses its K48‐linked ubiquitination and subsequent degradation in macrophages via blocking the recruitment of its major ubiquitination E3 Trim31. Consequently, neddylation promotes the activation of NLRP3 inflammasome and thereby contributes to DSS‐induced colitis and psychological stress‐induced anxiety.

A previous study reported the interaction of endogenous NEDD8 with endogenous Caspase‐1 in inflammasome‐activated macrophages [24]. However, we failed to detect the covalent conjugation of His‐NEDD8 to endogenous pro‐ and mature Caspase‐1 with histidine pulldown under fully denaturing conditions. Thus, the interaction of endogenous NEDD8 with endogenous Caspase‐1 in inflammasome‐activated macrophages might result from the assembly of neddylated NLRP3 into the complex with ASC and pro‐Caspase‐1.

Our findings suggest that neddylation in myeloid cells plays a significant role in exacerbating colonic inflammation and tissue damage. *Uba3* ^ΔMye^ mice exhibited reduced severity of DSS‐induced colitis. The attenuation of colitis severity in *Uba3* ^ΔMye^ mice was associated with a significant reduction in IL‐1β level but not TNF‐α and IL‐6 levels. These findings are consistent with previous studies that highlight the critical role of macrophage function in the progression of DSS‐induced colitis. Therefore, it is reasonable that MLN4924 administration has been demonstrated to alleviate DSS‐induced colitis [52, 53]. On the other hand, neddylation is also critical for the survival of intestinal epithelium upon treatment with a cytokine mixture via Cullin‐1‐dependent NF‐κB activation [54]. In TNBS‐induced colitis, the pathological mechanisms are more complicated. The disrupted epithelial barrier upon neddylation might play a predominant role in the reported enhancement of TNBS‐induced colitis by MLN4924 [54].

Recent studies revealed that psychological stress induces the activation of NLRP3 inflammasome in several brain regions important for emotion control [32, 33]. At the cellular level, NLRP3 inflammasome activation was predominantly detected in microglia and tissue‐resident macrophages in the central nervous system [32, 33]. Since our findings indicate that neddylation promotes NLRP3 inflammasome activation through stabilizing NLRP3 in macrophages, it is reasonable to propose that it can be a target for the intervention of psychological stress‐induced anxiety. Indeed, inducible neddylation blockade in microglia mitigates psychological stress‐induced anxiety‐like behavior. Neddylation inhibitor MLN4924 is in phase 1/2/3 clinical trials for cancers [49, 50, 51]. Our findings that MLN4924 alleviates psychological stress‐induced activation of NLRP3 inflammasome, microglia inflammatory activation, and anxiety‐like behavior, suggest novel clinical activity of MLN4924. In the reported protective role of MLN4924 in the colon, a dose of 15 mg/kg or less was used [52, 53]. However, a high dose at 60 mg/kg is required for MLN4924 to exert protective effects in the brain, possibly due to the existence of blood brain barrier [55]. Here, 50 mg/kg MLN4924 was intraperitoneally administrated 12 h before the 1, 3, 5, and seventh sessions of restraint. Neddylation has been implicated in various cellular processes, including metabolism and neurodevelopment [23]. Furthermore, off‐target effects of MLN4924 have been reported [56]. Thus, it is highly possible that prolonged systemic MLN4924 treatment at such a high dose triggers safety issues. The dose and the intervention procedure of MLN4924 should be optimized in the future.

## 4 Experimental Section/Methods

### 4.1 Mice

*Uba3* ^F/F; Lyz2‐Cre^ (*Uba3* ^ΔMye^), *Nedd8* ^F/F; Lyz2‐Cre^ (*Nedd8* ^ΔMye^), and 6 × His‐FLAG‐NEDD8 constitutive knock‐in mice on the C57BL/6 background have been reported previously [26, 27, 41]. *Uba3* ^F/F^ mice were crossed with *Cx3cr1‐CreERT2* mice on the C57BL/6 background (Shanghai Model Organisms Center, Inc.) to generate *Uba3* ^F/F; Cx3cr1‐CreERT2^ (*Uba3‐*iKO) mice. All mice were bred and maintained under specific pathogen‐free conditions with a 12 h light/dark cycle and were provided with free access to a standard laboratory diet and water. The animal experiments conducted in this study were approved by the Animal Care and Use Committee of Beijing Institute of Basic Medical Research (Permit number: IACUC‐DWZX‐2019‐521). All experimental procedures were carried out in accordance with the relevant standards and regulations set by the International Association for Laboratory Animal Evaluation and Certification.

### 4.2 Cell Culture and Transfection

The induction of BMDMs was performed as previously described [57]. BMDMs were primed with 100 ng/mL LPS (Cat# 2888, Sigma–Aldrich) for 4 h, followed by treatment with 5 mM ATP (Cat# tlrl‐atp, InvivoGen) for 30 min or 200 ng/mL MSU (Cat# tlrl‐msu, InvivoGen) for 6 h. HEK‐293T cells (Cat# CRL‐3216, ATCC) were cultured in DMEM complete medium at 37°C and 5% CO_2_. MG132 (Cat# C2211, Sigma–Aldrich), MLN4924 (Cat# 1231, Active Biochem), and TAK243 (Cat# S8341, Selleck) were dissolved in DMSO. Whenever MG132 or MLN4924 was used, an equal volume of DMSO was included as the control. Mammalian expression vector encoding Myc‐tagged human NLRP3 (Cat# HG11906‐NM) was purchased from Sino Biological Inc. Mutations were generated through overlapping PCR and confirmed by DNA sequencing. Mammalian expression vectors encoding HA‐tagged human Ub and its K48‐only (Cat# 17605) and K48R (Cat# 17604) mutants were obtained from Addgene [47]. Other mammalian or prokaryotic expression vectors used in this study were generated by cloning PCR‐amplified products of human cDNAs into the pcDNA3.1 (+) vector, pEGFP‐N1 vector, and pGEX‐KG vector, respectively, and confirmed by DNA sequencing. Murine Trim31 siRNA (5’‐GCUCAC UAAAUCCUUGAAA‐3’), murine Smurf2 siRNAs (5’‐CAACGCAGCAGGGTCAGGUAU AUUU‐3’ and 5’‐GCAGCAGGG UCAGGUAUAUUUCUUA), and non‐targeting control siRNA (5’‐UUCUCCGAACGUGUCACGU‐3’) were ordered from Shanghai GenePharma Co., Ltd. Transfection of plasmids and siRNAs were performed with jetPRIME (Cat# 101000046, Polyplus) and Lipofectamine RNAiMax (Cat# 13778075, Invitrogen), respectively, according to the manufacturers’ protocols.

### 4.3 ELISA

The colon tissue was homogenized as previously described [58], and the supernatants were collected to measure cytokine levels. TNF‐α, IL‐6, and IL‐1β levels in the supernatants of homogenized colon tissues or cultured macrophages were assayed with commercial kits (Cat# 88‐7324‐88, 88‐7064‐88, and 88‐7013A‐77, eBioscience), according to the manufacturer's protocols.

### 4.4 Microarray‐Based Transcriptome Analysis

RNA was labeled with Cy5 dyes (Amersham Pharmacia) and hybridized to Mouse Whole Genome OneArray with Phalanx hybridization buffer using Phalanx Hybridization System (Phalanx Biotech). Data were analyzed according to the manufacturer's protocol and deposited in NCBI's GEO database with accession number GSE290182 and reviewer token yterykealjwzfyn.

### 4.5 Quantitative RT‐PCR

Total RNA was extracted using TRIzol reagent (Cat# T9414, Sigma–Aldrich). cDNA was synthesized using a cDNA synthesis kit (Cat# RK204, Abclonal). Quantitative RT‐PCR was then performed in Roche LightCycler 480 fluorescence quantitative PCR instrument with SYBR Green Realtime PCR reagent (Cat# QPK‐101, TOYOBO). The primers are: murine *Tnf*, forward 5’‐CAGCCTCTTCTCATTCCTGC‐3’ and reverse 5’‐GGTCTGGGCCATAGAACTGA‐3’; murine *Il6*, forward 5’‐GATGGATGCTACCAAACT GGA‐3’ and reverse 5’‐TCTGAAGGACTCTGGCTTTG‐3’; murine *Il1b*, forward 5‐TGAAGCAGCTATGGCAACTG‐3’ and reverse 5’‐ AGGTCAAAGGTTTGGAAGCA‐3’; murine *Nlrp3*, forward 5‐ATTACCCGCCCGAGAAAGG‐3’ and reverse 5’‐TCGCAG CAAAGATCCACACAG‐3’; and murine *Gapdh*, forward 5’‐ TCTTGGGCTACACTGAG GAC‐3’ and reverse 5’‐CATACCAGGAAATGAGCTTGA‐3’. The fold change in mRNA levels, normalized to *Gapdh* and relative to the expression in the control group, was calculated for each sample as 2^−ΔΔCT^.

### 4.6 Immunoblotting (IB) and Immunoprecipitation (IP)

Immunoblotting was carried out in RIPA buffer (50 mM Tris‐HCl, pH 7.5, 1% NP40, 0.35% DOC, 150 mm NaCl, 1 mm EDTA, 1 mM EGTA, supplemented with protease and phosphatase inhibitor cocktails). After SDS‐PAGE, the proteins were transferred to Hybond‐P polyvinylidene difluoride membranes. The membranes were initially incubated with primary antibodies at 4°C overnight, and then with horseradish peroxidase‐conjugated secondary antibodies for 1 h at 25°C. Bound antibody was detected using SuperSignal West Pico PLUS Chemiluminescent Substrate. Co‐IP was carried out in IP lysis buffer (10 mm Tris‐HCl, pH 7.5, 2 mm EDTA, 1% NP40, 150 mm NaCl, supplemented with protease and phosphatase inhibitor cocktail). 1.0 mg protein of cell lysates/sample was then used for IP. Immunoprecipitated proteins were washed with IP lysis buffer containing 500 mm NaCl for three times, followed by IB. In vivo neddylation/ubiquitination assay with IP was performed as previously described [41]. Antibodies against murine Caspase‐1 (Cat# AG‐20B‐0042) and NLRP3 (Cat# AG‐20B‐0014) were ordered from Adipogen. Antibodies against NEDD8 (Cat# ab81264), Synaptophysin (Cat# ab14692), UBA3 (Cat# ab124728), human Caspase‐1 (Cat# ab207802), and GSDMD (Cat# ab209845) were from Abcam. Antibodies against IL‐1β (Cat# A1112) and Trim31 (Cat# A10639) were from Abclonal. Antibodies against phospho‐JNK (P‐JNK, Cat# 4671) at Thr183/Tyr185 and LC3B (Cat# 2775) were from Cell Signaling Technology. Antibodies against PSD‐95 (Cat# 20665‐1‐AP), p53 (Cat# 10442‐1‐AP), Ube2M (Cat# 14520‐1‐AP), GST (Cat# 66001‐2‐Ig), Smurf2 (Cat# 18038‐1‐AP), and Ub (Cat# 10201‐2‐AP) were from Proteintech. Antibody against Myc (Cat# M192‐3) was from MBL International. Antibody against FLAG (Cat# F1804) was from Sigma–Aldrich. Antibodies against IκBα (Cat# sc‐371), ASC (Cat# sc‐514414), and β‐actin (Cat# sc‐8432) were from Santa Cruz. The smear band cut from the SDS‐PAGE gel after IP (Figure S6) was sent to Shanghai OE Biotech Co., Ltd for mass spectrometry (LC‐MS/MS) analysis.

### 4.7 Establishment of DSS‐Induced Colitis

Ten‐week‐old male mice and their littermate controls were administered 3% DSS dissolved in drinking water for 7 consecutive days to induce colitis. Weight loss was recorded daily. On day 7, disease activity index was calculated by assessing body weight loss (score: 0, none; 1, 1%–5%; 2, 6%–10%; 3, 11%–18%; 4, >18%), stool consistency (score: 0, normal; 1, soft but still formed; 2, soft and loose stools; 3, very soft and wet; 4, watery diarrhea), and the presence of gross bleeding (score: 0, normal; 1 and 2, focal bloody stool; 3, blood traces in stool; 4, gross rectal bleeding) [10]. Then all animals were sacrificed. A portion of the colon tissue was stored directly at −80°C for ELISA analysis, while the remaining colon tissue was subjected to histology.

### 4.8 Histology

Colon tissues were removed from mice and fixed in 10% buffered formalin for at least 24 h, dehydrated, and infiltrated with paraffin. 5 µm paraffin sections were then prepared and stained with hematoxylin and eosin (H & E; Cat# G1005, Servicebio) for brightfield microscopy. Histological scoring was performed based on previously described criteria as follows: 0, normal epithelial cells with no signs of leukocyte infiltration; 1, absence of goblet cells and leukocyte infiltration around crypts; 2, widespread absence of goblet cells and leukocyte infiltration into the mucosal muscle layer; 3, mild crypt loss, significant leukocyte infiltration into the mucosal muscle layer, accompanied by mucosal thickening; 4, severe crypt loss or polypoid regeneration, with leukocyte infiltration extending into the submucosa [59].

### 4.9 Flow Cytometry

The colon was washed of fecal content and then was opened longitudinally and cut into pieces about 1 cm in length, followed by incubation on a shaker at 250 rpm for 15 min at 37°C in PBS supplemented with 5% FBS, 1 mm EDTA, and 1 mm DTT. Thereafter, colon tissues were digested on a shaker at 170 rpm for 60 min at 37°C in a culture medium containing 0.6 mg/mL type IV collagenase (Cat# C5138, Sigma–Aldrich) and 0.01 mg/mL DNase I (Cat# DN25, Sigma–Aldrich), according to a previous report [60]. After being filtered through a 70 µm cell strainer, cell suspensions were centrifuged at 300 g, 4°C for 5 min. The pellets were suspended with 35% Percoll (Cat# 17‐0891‐09, GE Healthcare) solution in RPMI‐1640 medium and centrifuged at 500 *g*, 4°C for 5 min. After depletion of red blood cells, single‐cell suspensions were washed once with FACS washing buffer (2% FBS and 0.1% NaN3 in PBS). Cells were then incubated with fluorescence‐conjugated antibodies PerCP‐CD45 (Cat# 103130, BioLegend), BV510‐CD11b (Cat# 101263, BioLegend), and PE‐F4/80 (Cat# 111604, BioLegend) for 30 min on ice. To determine cell viability, Ghost Dye Red 780 Viability Dye (Cat# 13‐0865‐T100, CYTEK) was used according to the manufacturer's instructions. After washing with FACS buffer, flow cytometry was immediately performed using a Becton Dickinson FACS Fortessa machine, and the data were analyzed using the FlowJo version 10.

### 4.10 Restraint Stress Model

Adult male mice were placed in 50 mL conical tubes with 0.5 cm holes for airflow, from 0:00 to 8:00 a.m. each day, in separate sound‐ and light‐attenuating boxes for 8 consecutive days. Once the restraint ended, mice were put back to their home cages immediately with access to food and water freely. For the control group, the mice were placed in the home cage at the same time, without food, and water.

### 4.11 Open Field Test (OFT)

OFT was conducted in a white plastic open‐field arena (50 cm × 50 cm × 40 cm). The bottom was divided into a 30 cm × 30 cm central area and the surrounding border zone. Mice were adapted to the dark and sound‐insulated experimental room for 1 h prior to testing. Then mice were individually gently placed in the center and free to explore the area for 5 min. A video camera positioned directly above the arena was used to track the movement of each mouse. A computer with software (Any‐maze by Stoelting) was used to record their entries into the central area, and the total distance, and the amount of time spent there. The arena was cleaned with 70% ethanol after every trial.

### 4.12 Elevated Plus Maze Test (EPT)

EPT was conducted in a 4‐arm maze with two open arms without walls and two closed arms with walls (25 cm long, 5 cm wide). This structure was elevated 60 cm above the floor. Mice were adapted to the dark and sound‐insulated experimental room for 1 h prior to testing. Then mice were individually placed in the center and faced to a closed arm at the start of a trial. A video camera positioned directly above the arena was used to track the movement of each mouse throughout a 5 min session. A computer with software (Any‐maze by Stoelting) was used to record their entries into the open arm, the total distance, and the amount of time spent there. The arena was cleaned with 70% ethanol after every trial.

### 4.13 Indirect Immunofluorescence

Brain tissues embedded in OCT (Cat# 23‐730‐571, Thermo Fisher Scientific) were prepared, and immunofluorescence staining of 10 µm thick amygdala coronal sections was performed as previously described [61]. Briefly, Samples were then incubated with antibodies against Iba1 (1:200, Cat# 019‐19741, Wako Chemicals) and CD68 (1:200, Cat# 97778, Cell Signaling Technology) diluted in blocking buffer overnight at 4°C. After being washed three times in PBS, the samples were incubated with fluorescence‐conjugated secondary antibodies for 45 min at room‐temperature. ImageJ was used to calculate the co‐labeling of Iba1 and CD68.

### 4.14 Measurement of ROS Generation

Measurement of ROS generation was performed as previously described [62]. Briefly, BMDMs were homogenized in IP lysis buffer. After centrifugation, the supernatant was mixed with the fluorescent probe DCFH‐DA (Cat# D6883, Sigma–Aldrich) to a final concentration of 5 µm. The mixture was incubated in the dark for 1 h at 37°C. DCFH‐DA is converted to DCFH by intracellular esterase. DCFH is oxidized by ROS to the strong fluorescent DCF. Finally, the DCF fluorescence intensity was measured using a Spectrophotometer (VICTOR X3 2030 Multilabel Reader, PerkinElmer) with an excitation and emission wavelength of 485 and 530 nm, respectively. Data were normalized to protein concentration as determined by the Bradford assay.

### 4.15 Immunohistochemistry

Clinical non‐colitis control colon tissues (*n* = 12) and colon tissues with chronic colitis (*n* = 11) on tissue microarray slides (Cat# DP096Co01) were obtained from Xi'an Bioaitech Co., Ltd. Patients’ consent and approval by the Institional Review Board of Beijing Institute of Basic Medical Sciences (Permit number: AF/SC‐08/02.249) were obtained for the use of the clinical materials in research. Immunohistochemistry was performed using standard protocols with citrate buffer (pH 6.0) pretreatment. Briefly, formaldehyde‐fixed and paraffin‐embedded sections were incubated with an antibody against NEDD8 (Cat# ab81264, Abcam) or NLRP3 (Cat# HPA012878, Sigma–Aldrich) at 4°C overnight and then with a horseradish peroxidase‐conjugated goat anti‐mouse antibody at 37°C for 30 min. The sections were finally incubated with diaminobenzidine and counterstained with hematoxylin for detection. The array images were captured with a panoramic scanner pannoramic (3DHISTECH) using the software CaseViewer 2.4 (3DHISTECH). H‐score was analyzed with the software Aipathwell (Servicebio). H‐score = (percentage of weak intensity × 1) + (percentage of moderate intensity × 2) + (percentage of strong intensity × 3).

### 4.16 In Vivo Neddylation Assay with Histidine Pulldown

Cells were solubilized in buffer A (6 m guanidine‐HCl, 0.1 m Na_2_HPO_4_/NaH_2_PO_4_, 10 mm imidazole, pH 8.0). After sonication, cell lysates were incubated with nickel‐nitrilotriacetic acid (Ni‐NTA) matrices (Cat# 30210, Qiagen) overnight at room‐temperature. Histidine pulldown products were washed sequentially once in buffer A, twice in buffer A/TI mixture (buffer A:buffer TI = 1:3), and once in buffer TI (25 mm Tris‐HCl and 20 mm imidazole, pH 6.8). Precipitates were then subjected to IB analysis [41].

### 4.17 In Vitro Neddylation/Ubiquitination Assay

GST‐NLRP3 WT and K287R were expressed and purified with glutathione‐agarose beads (Cat# G4510, Sigma–Aldrich). In vitro neddylation of GST‐NLRP3 (200 ng in each sample, 37°C, 1 h) was performed with a commercial kit (Cat# BML‐UW0590, Enzo Life Sciences), according to the manufacturer's instructions. For in vitro ubiquitination, GFP‐Trim31 immunoprecipitated from HEK‐293T cells was incubated with purified GST‐NLRP3 WT, Ub, E1 UBA1, and E2 UBCH5A in the reaction buffer provided by the in vitro neddylation kit at 37°C for 1 h. The tubes were tapped every 5 min. The neddylation/ ubiquitination of GST‐NLRP3 was analyzed by IB.

### 4.18 Statistical Analysis

Error bars represent mean ± standard deviations (SD) and were assessed by two‐tailed Student's *t* test or two‐way ANOVA. Statistical calculations were performed using GraphPad Prism 8.3.0. *P* values of less than 0.05 were considered statistically significant.

## Funding

This study is supported by grants from the National Natural Science Foundation of China (82530063, 92169207, and 81930027).

## Conflicts of Interest

The authors declare no conflicts of interest.

## Supporting information




**Supporting File 1**: advs73747‐sup‐0001‐SuppMat.docx.

**Supporting File 2**: advs73747‐sup‐0002‐Data.zip.

## Data Availability

All data are included in the article and/or supplemental information. In addition, microarray‐based transcriptome data were deposited in NCBI's GEO database with accession number GSE290182 and reviewer token yterykealjwzfyn. For IP‐MS, all raw data and search results have been deposited to the PRIDE database (http://www.iprox.org/index) with the accession number IPX0013333000.

## References

[1] J. Fu and H. Wu , “Structural Mechanisms of NLRP3 Inflammasome Assembly and Activation,” Annual Review of Immunology 41 (2023): 301–316, 10.1146/annurev-immunol-081022-021207. PMC10159982 36750315

[2] P. Broz and V. M. Dixit , “Inflammasomes: Mechanism of Assembly, Regulation and Signalling,” Nature Reviews Immunology 16, no. 7 (2016): 407–420, 10.1038/nri.2016.58. 27291964

[3] U. C. Frising , S. Ribo , M. G. Doglio , B. Malissen , G. van Loo , and A. Wullaert , “Nlrp3 Inflammasome Activation in Macrophages Suffices for Inducing Autoinflammation in Mice,” EMBO Reports 23, no. 7 (2022): 54339, 10.15252/embr.202154339. PMC9253760 35574994

[4] S. Mariathasan , D. S. Weiss , K. Newton , et al., “Cryopyrin Activates the Inflammasome in Response to Toxins and ATP,” Nature 440, no. 7081 (2006): 228–232, 10.1038/nature04515. 16407890

[5] F. Martinon , V. Pétrilli , A. Mayor , A. Tardivel , and J. Tschopp , “Gout‐Associated Uric Acid Crystals Activate the NALP3 Inflammasome,” Nature 440, no. 7081 (2006): 237–241, 10.1038/nature04516. 16407889

[6] P. Duewell , H. Kono , K. J. Rayner , et al., “NLRP3 Inflammasomes Are Required for Atherogenesis and Activated by Cholesterol Crystals,” Nature 464, no. 7293 (2010): 1357–1361, 10.1038/nature08938. 20428172 PMC2946640

[7] F. Martinon , A. Mayor , and J. Tschopp , “The Inflammasomes: Guardians of the Body,” Annual Review of Immunology 27 (2009): 229–265, 10.1146/annurev.immunol.021908.132715. 19302040

[8] J. Shi , Y. Zhao , K. Wang , et al., “Cleavage of GSDMD by Inflammatory Caspases Determines Pyroptotic Cell Death,” Nature 526, no. 7575 (2015): 660–665, 10.1038/nature15514. 26375003

[9] J.‐S. Kim , H. K. Kim , J. Lee , et al., “Inhibition of CD82 Improves Colitis by Increasing NLRP3 Deubiquitination by BRCC3,” Cellular & Molecular Immunology 20, no. 2 (2023): 189–200, 10.1038/s41423-022-00971-1. 36600050 PMC9887069

[10] X. Liu , Y. Fang , X. Lv , et al., “Deubiquitinase OTUD6A in Macrophages Promotes Intestinal Inflammation and Colitis via Deubiquitination of NLRP3,” Cell Death & Differentiation 30, no. 6 (2023): 1457–1471, 10.1038/s41418-023-01148-7. 36932155 PMC10244424

[11] S. Huang , W. Dong , X. Lin , et al., “Disruption of the Na+/K+‐ATPase‐Purinergic P2X7 Receptor Complex in Microglia Promotes Stress‐Induced Anxiety,” Immunity 57, no. 3 (2024): 495–512, 10.1016/j.immuni.2024.01.018. 38395698

[12] Y.‐L. Wang , Q.‐Q. Han , W.‐Q. Gong , et al., “Microglial Activation Mediates Chronic Mild Stress‐Induced Depressive‐ and Anxiety‐Like Behavior in Adult Rats,” Journal of Neuroinflammation 15, no. 1 (2018): 21, 10.1186/s12974-018-1054-3. 29343269 PMC5773028

[13] C. J. Groß , R. Mishra , K. S. Schneider , et al., “K+ Efflux‐Independent NLRP3 Inflammasome Activation by Small Molecules Targeting Mitochondria,” Immunity 45, no. 4 (2016): 761–773. 27692612 10.1016/j.immuni.2016.08.010

[14] K. Tobiume , A. Matsuzawa , T. Takahashi , et al., “ASK1 Is Required for Sustained Activations of JNK/p38 MAP Kinases and Apoptosis,” EMBO Reports 2, no. 3 (2001): 222–228, 10.1093/embo-reports/kve046. 11266364 PMC1083842

[15] N. Song , Z.‐S. Liu , W. Xue , et al., “NLRP3 Phosphorylation Is an Essential Priming Event for Inflammasome Activation,” Molecular Cell 68, no. 1 (2017): 185–197.e6, 10.1016/j.molcel.2017.08.017. 28943315

[16] D. Wang , Y. Zhang , X. Xu , et al., “YAP Promotes the Activation of NLRP3 Inflammasome via Blocking K27‐Linked Polyubiquitination of NLRP3,” Nature Communications 12, no. 1 (2021): 2674, 10.1038/s41467-021-22987-3. PMC8113592 33976226

[17] H. Song , B. Liu , W. Huai , et al., “The E3 Ubiquitin Ligase TRIM31 Attenuates NLRP3 Inflammasome Activation by Promoting Proteasomal Degradation of NLRP3,” Nature Communications 7 (2016): 13727, 10.1038/ncomms13727. PMC5155141 27929086

[18] J. Tang and S. Tu , “Sequential Ubiquitination of NLRP3 by RNF125 and Cbl‐b Limits Inflammasome Activation and Endotoxemia,” Journal of Experimental Medicine 217, no. 4 (2020): 20182091, 10.1084/jem.20182091. PMC7144527 31999304

[19] T. Tang , P. Li , X. Zhou , et al., “The E3 Ubiquitin Ligase TRIM65 Negatively Regulates Inflammasome Activation Through Promoting Ubiquitination of NLRP3,” Frontiers in Immunology 12 (2021): 741839, 10.3389/fimmu.2021.741839. 34512673 PMC8427430

[20] P. Wan , Q. Zhang , W. Liu , et al., “Cullin1 Binds and Promotes NLRP3 Ubiquitination to Repress Systematic Inflammasome Activation,” The FASEB Journal 33, no. 4 (2019): 5793–5807, 10.1096/fj.201801681R. 30653357

[21] H. Walden , M. S. Podgorski , D. T. Huang , et al., “The Structure of the APPBP1‐UBA3‐NEDD8‐ATP Complex Reveals the Basis for Selective Ubiquitin‐Like Protein Activation by an E1,” Molecular Cell 12, no. 6 (2003): 1427–1437, 10.1016/S1097-2765(03)00452-0. 14690597

[22] W. Zhou , J. Xu , M. Tan , et al., “UBE2M Is a Stress‐Inducible Dual E2 for Neddylation and Ubiquitylation that Promotes Targeted Degradation of UBE2F,” Molecular Cell 70, no. 6 (2018): 1008–1024, 10.1016/j.molcel.2018.06.002. 29932898 PMC6021141

[23] T. Zou and J. Zhang , “Diverse and Pivotal Roles of Neddylation in Metabolism and Immunity,” The FEBS Journal 288, no. 13 (2021): 3884–3912, 10.1111/febs.15584. 33025631

[24] M. A. Read , J. E. Brownell , T. B. Gladysheva , et al., “Nedd8 Modification of Cul‐1 Activates SCF βTrCP ‐Dependent Ubiquitination of IκBα,” Molecular and Cellular Biology 20, no. 7 (2000): 2326–2333, 10.1128/MCB.20.7.2326-2333.2000. 10713156 PMC85397

[25] J. A. Segovia , S.‐Y. Tsai , T.‐H. Chang , et al., “Nedd8 Regulates Inflammasome‐Dependent Caspase‐1 Activation,” Molecular and Cellular Biology 35, no. 3 (2015): 582–597, 10.1128/MCB.00775-14. 25452302 PMC4285429

[26] M. Zhao , Y. Zhang , X. Yang , et al., “Myeloid Neddylation Targets IRF7 and Promotes Host Innate Immunity Against RNA Viruses,” PLoS Pathogens 17, no. 9 (2021): 1009901, 10.1371/journal.ppat.1009901. PMC8432861 34506605

[27] X. Zhang , Z. Ye , Y. Pei , et al., “Neddylation Is Required for Herpes Simplex Virus Type I (HSV‐1)‐induced Early Phase Interferon‐Beta Production,” Cellular & Molecular Immunology 13, no. 5 (2016): 578–583, 10.1038/cmi.2015.35. 27593482 PMC5037273

[28] F. Charlson , M. van Ommeren , A. Flaxman , J. Cornett , H. Whiteford , and S. Saxena , “New WHO Prevalence Estimates of Mental Disorders in Conflict Settings: A Systematic Review and Meta‐analysis,” The Lancet 394, no. 10194 (2019): 240–248, 10.1016/S0140-6736(19)30934-1. PMC6657025 31200992

[29] B. Rodrigues , R. A. Leitão , M. Santos , et al., “MiR‐186‐5p Inhibition Restores Synaptic Transmission and Neuronal Network Activity in a Model of Chronic Stress,” Molecular Psychiatry 30, no. 3 (2025): 1034–1046, 10.1038/s41380-024-02715-1. 39237722 PMC11835755

[30] V. Sahasrabuddhe and H. S. Ghosh , “Cx3Cr1‐Cre Induction Leads to Microglial Activation and IFN‐1 Signaling Caused by DNA Damage in Early Postnatal Brain,” Cell Reports 38, no. 3 (2022): 110252, 10.1016/j.celrep.2021.110252. 35045285

[31] K.‐Q. Fan , Y.‐Y. Li , H.‐L. Wang , et al., “Stress‐Induced Metabolic Disorder in Peripheral CD4+ T Cells Leads to Anxiety‐Like Behavior,” Cell 179, no. 4 (2020): 864–879, 10.1016/j.cell.2019.10.001. 31675497

[32] G. S. Chiu , P. T. Darmody , J. P. Walsh , et al., “Adenosine Through the A2A Adenosine Receptor Increases IL‐1β in the Brain Contributing to Anxiety,” Brain, Behavior, and Immunity 41 (2014): 218–231, 10.1016/j.bbi.2014.05.018. 24907587 PMC4167209

[33] C.‐Y. Wang , S.‐Y. Jiang , S.‐M. Liao , et al., “Dimethyl Fumarate Ameliorates Chronic Stress‐Induced Anxiety‐Like Behaviors by Decreasing Neuroinflammation and Neuronal activity in the Amygdala,” International Immunopharmacology 137 (2024): 112414, 10.1016/j.intimp.2024.112414. 38897132

[34] A. Mishra , H. J. Kim , A. H. Shin , and S. A. Thayer , “Synapse Loss Induced by Interleukin‐1β Requires Pre‐ and Post‐synaptic Mechanisms,” Journal of Neuroimmune Pharmacology 7, no. 3 (2017): 571–578, 10.1007/s11481-012-9342-7. PMC3415563 22311599

[35] B. Parajuli , Y. Sonobe , H. Horiuchi , H. Takeuchi , T. Mizuno , and A. Suzumura , “Oligomeric Amyloid β Induces IL‐1β Processing via Production of ROS: Implication in Alzheimer's Disease,” Cell Death & Disease 4, no. 12 (2013): 975, 10.1038/cddis.2013.503. PMC3877570 24357806

[36] S. E. Kwon , “Synaptophysin Regulates the Kinetics of Synaptic Vesicle Endocytosis in Central Neurons,” Neuron 70, no. 5 (2011): 847–854, 10.1016/j.neuron.2011.04.001. 21658579 PMC3136197

[37] L. Matt , K. Kim , A. C. Hergarden , et al., “α‐Actinin Anchors PSD‐95 at Postsynaptic Sites,” Neuron 97, no. 5 (2018): 1094–1109, 10.1016/j.neuron.2018.01.036. 29429936 PMC5963734

[38] T. Zhu , J. Wang , Y. Pei , et al., “Neddylation Controls Basal MKK7 Kinase Activity in Breast Cancer Cells,” Oncogene 35, no. 20 (2016): 2624–2633, 10.1038/onc.2015.323. 26364603

[39] R. Hjerpe , Y. Thomas , J. Chen , et al., “Changes in the Ratio of Free NEDD8 to Ubiquitin Triggers NEDDylation by Ubiquitin Enzymes,” Biochemical Journal 441, no. 3 (2012): 927–939, 10.1042/BJ20111671. 22004789 PMC3280039

[40] M. L. Hyer , M. A. Milhollen , J. Ciavarri , et al., “A Small‐molecule Inhibitor of the Ubiquitin Activating Enzyme for Cancer Treatment,” Nature Medicine 24, no. 2 (2018): 186–193, 10.1038/nm.4474. 29334375

[41] X. Zhang , Y.‐L. Zhang , G. Qiu , et al., “Hepatic Neddylation Targets and Stabilizes Electron Transfer Flavoproteins to Facilitate Fatty Acid β‐Oxidation,” Proceedings of the National Academy of Sciences 117, no. 5 (2020): 2473–2483, 10.1073/pnas.1910765117. PMC7007566 31941714

[42] D. P. Xirodimas , M. K. Saville , J. C. Bourdon , R. T. Hay , and D. P. Lane , “Mdm2‐Mediated NEDD8 Conjugation of p53 Inhibits its Transcriptional Activity,” Cell 118, no. 1 (2004): 83–97, 10.1016/j.cell.2004.06.016. 15242646

[43] I. Aoki , M. Higuchi , and Y. Gotoh , “NEDDylation Controls the Target Specificity of E2F1 and Apoptosis Induction,” Oncogene 32, no. 34 (2013): 3954–3964, 10.1038/onc.2012.428. 23001041

[44] J. Shu , C. Liu , R. Wei , P. Xie , S. He , and L. Zhang , “Nedd8 Targets Ubiquitin Ligase Smurf2 for Neddylation and Promote its Degradation,” Biochemical and Biophysical Research Communications 474, no. 1 (2016): 51–56, 10.1016/j.bbrc.2016.04.058. 27086113

[45] L. Xu , X. Lyu , Y. Wang , et al., “Neddylation Modification Stabilizes LC3B by Antagonizing its Ubiquitin‐Mediated Degradation and Promoting Autophagy in Skin,” Proceedings of the National Academy of Sciences 122, no. 15 (2025): 2411429122, 10.1073/pnas.2411429122. PMC12012473 40208944

[46] G. Liu and D. P. Xirodimas , “NUB1 Promotes Cytoplasmic Localization of p53 Through Cooperation of the NEDD8 and Ubiquitin Pathways,” Oncogene 29, no. 15 (2010): 2252–2261, 10.1038/onc.2009.494. 20101219

[47] K. L. Lim , K. C. M. Chew , J. M. M. Tan , et al., “Parkin Mediates Nonclassical, Proteasomal‐Independent Ubiquitination of Synphilin‐1: Implications for Lewy Body Formation,” The Journal of Neuroscience 25, no. 8 (2005): 2002–2009, 10.1523/JNEUROSCI.4474-04.2005. 15728840 PMC6726069

[48] W. Zhao , C.‐S. Shi , K. Harrison , et al., “AKT Regulates NLRP3 Inflammasome Activation by Phosphorylating NLRP3 Serine 5,” The Journal of Immunology 205, no. 8 (2020): 2255–2264, 10.4049/jimmunol.2000649. 32929041 PMC7541779

[49] J. H. Foster , J. M. Reid , C. Minard , et al., “Phase 1 Study of NEDD8 Activating Enzyme Inhibitor Pevonedistat in Combination With Chemotherapy in Pediatric Patients With Recurrent or Refractory Solid Tumors (ADVL1615),” European Journal of Cancer 209 (2024): 114241, 10.1016/j.ejca.2024.114241. 39096851 PMC11392690

[50] A. Qin , L. Wells , B. Malhotra , et al., “A Phase II Trial of Pevonedistat and Docetaxel in Patients With Previously Treated Advanced Non–Small‐Cell Lung Cancer,” Clinical Lung Cancer 25, no. 2 (2024): 128–134, 10.1016/j.cllc.2023.10.011. 37977950

[51] L. Adès , L. Girshova , V. A. Doronin , et al., “Pevonedistat plus Azacitidine vs Azacitidine Alone in Higher‐Risk MDS/chronic Myelomonocytic Leukemia or Low‐blast‐percentage AML,” Blood Advances 6, no. 17 (2022): 5132–5145. 35728048 10.1182/bloodadvances.2022007334 PMC9631625

[52] V. F. Curtis , S. F. Ehrentraut , E. L. Campbell , et al., “Stabilization of HIF Through Inhibition of Cullin‐2 Neddylation is Protective in Mucosal Inflammatory Responses,” The FASEB Journal 29, no. 1 (2015): 208–215, 10.1096/fj.14-259663. 25326537 PMC4285538

[53] P. Wan , X.‐D. Zhu , X.‐P. Liu , et al., “Inhibition of Neddylation Ameliorates DSS‐Induced Colitis,” Cellular & Molecular Immunology 15, no. 6 (2018): 649–650, 10.1038/cmi.2017.144. 29375125 PMC6078971

[54] S. F. Ehrentraut , V. F. Curtis , R. X. Wang , et al., “Perturbation of Neddylation‐Dependent NF‐κB Responses in the Intestinal Epithelium Drives Apoptosis and Inhibits Resolution of Mucosal Inflammation,” Molecular Biology of the Cell 27, no. 23 (2016): 3687–3694, 10.1091/mbc.e16-05-0273. 27682585 PMC5170552

[55] H. Yu , H. Luo , L. Chang , et al., “The NEDD8‐Activating Enzyme Inhibitor MLN4924 Reduces Ischemic Brain Injury in Mice,” Proceedings of the National Academy of Sciences 119, no. 6 (2022): 2111896119, 10.1073/pnas.2111896119. PMC8833173 35101976

[56] S. Zhang , X. You , T. Xu , et al., “PD‐L1 Induction via the MEK‐JNK‐AP1 Axis by a Neddylation Inhibitor Promotes Cancer‐Associated Immunosuppression,” Cell Death & Disease 13, no. 10 (2022): 844, 10.1038/s41419-022-05292-9. 36192389 PMC9529958

[57] J. Zhang , N. Zhu , Q. Wang , et al., “MEKK3 Overexpression Contributes to the Hyperresponsiveness of IL‐12–Overproducing Cells and CD4+ T Conventional Cells in Nonobese Diabetic Mice,” The Journal of Immunology 185, no. 6 (2010): 3554–3563, 10.4049/jimmunol.1000431. 20720201

[58] G. Liu , Q. Wang , L. Deng , et al., “Hepatic RACK1 Deficiency Protects Against Fulminant Hepatitis Through Myeloid‐derived Suppressor Cells,” Theranostics 12, no. 5 (2022): 2248–2265, 10.7150/thno.65916. 35265209 PMC8899575

[59] C. Qu , Z.‐W. Yuan , X.‐T. Yu , et al., “Patchouli Alcohol Ameliorates Dextran Sodium Sulfate‐induced Experimental Colitis and Suppresses Tryptophan Catabolism,” Pharmacological Research 121 (2017): 70–82, 10.1016/j.phrs.2017.04.017. 28456683

[60] J. Du , J. Zhang , L. Wang , et al., “Selective Oxidative Protection Leads to Tissue Topological Changes Orchestrated by Macrophage During Ulcerative Colitis,” Nature Communications 14 (2023): 3675, 10.1038/s41467-023-39173-2. PMC10284839 37344477

[61] G. Xu , T. Zou , L. Deng , et al., “Nonerythropoietic Erythropoietin Mimetic Peptide ARA290 Ameliorates Chronic Stress‐Induced Depression‐Like Behavior and Inflammation in Mice,” Frontiers in Pharmacology 13 (2022): 896601, 10.3389/fphar.2022.896601. 36046815 PMC9421426

[62] C. Xu , H. Zhou , Y. Jin , et al., “Hepatic Neddylation Deficiency Triggers Fatal Liver Injury via Inducing NF‐κB‐Inducing Kinase in Mice,” Nature Communications 13 (2022): 7782, 10.1038/s41467-022-35525-6. PMC9758150 36526632

