# Supplementary material for: Neddylation Targets and Stabilizes NLRP3 to Augment Inflammasome‐Mediated Colitis and Mood Disorder
Source: Adv Sci (Weinh). 2026 Jan 9;13(16):e05906. doi: 10.1002/advs.202505906 (PMC13042396; doi:10.1002/advs.202505906)
Supplement: Supplementary file 1 — Supporting File 1: advs73747‐sup‐0001‐SuppMat.docx. [file ADVS-13-e05906-s002.docx]

Supporting Information

**Neddylation Targets and Stabilizes NLRP3 to Augment Inflammasome-mediated Colitis and Mood Disorder**

*Wenbin Gai, Mengyao Wu, Anbiao Wu, Zhaofei Jing, Zhenjie Ye, Jiayan Jin, Yaolin Zhang, Min Zhao, Genyu Liu, Xu Wang, Xiqin Yang, Jie Dong***, Yunlu Xu***,* and *Jiyan Zhang**


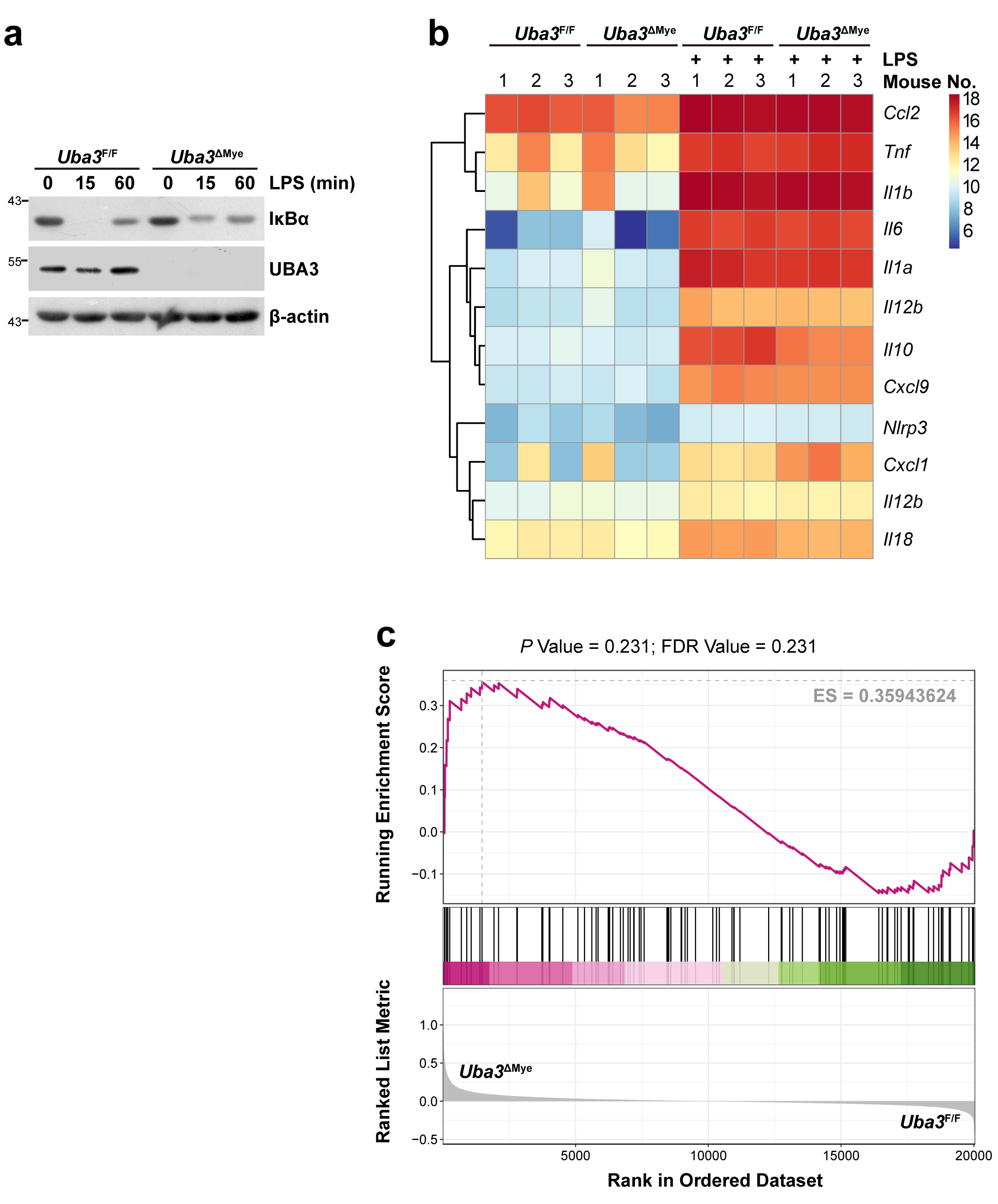


**Figure S1.** Effects of UBA3 deficiency on LPS-induced NF-κB activation in BMDMs. a)

BMDMs from *Uba3*^F/F^ and *Uba3*^ΔMye^ mice were stimulated with 100 ng/mL LPS for 0, 15, 60 min, followed IB analysis of IκBα, UBA3, and β-actin. b,c) BMDMs from *Uba3*^F/F^ and *Uba3*^ΔMye^ mice were stimulated with or without 100 ng/mL LPS for 4h, followed by microarray-based transcriptome analysis. mRNA levels of key NF-κB target genes are shown in the heatmap (b). Gene set enrichment analysis of NF-κB target genes in LPS-stimulated UBA3-sufficient and -deficient BMDMs (c).


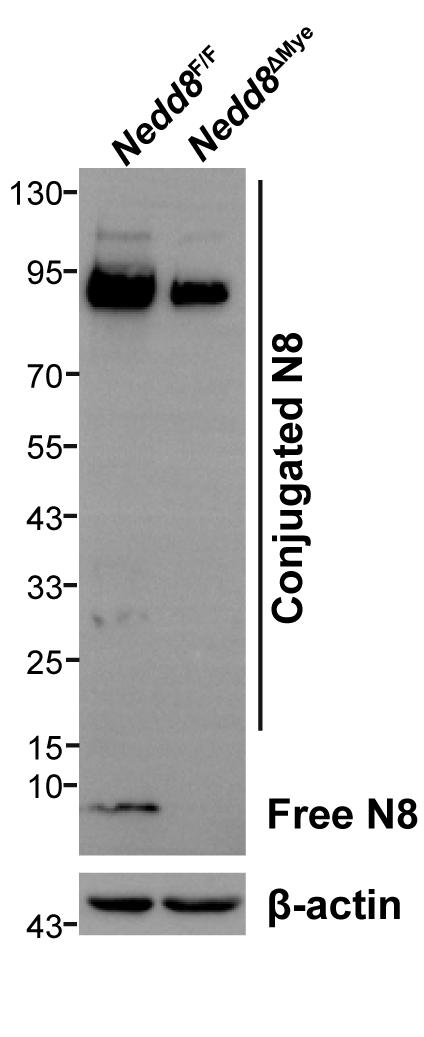


**Figure S2.** IB analysis of conjugated and free NEDD8 in BMDMs from *Nedd8*^F/F^ and *Nedd8*^ΔMye^ mice. N8, NEDD8.


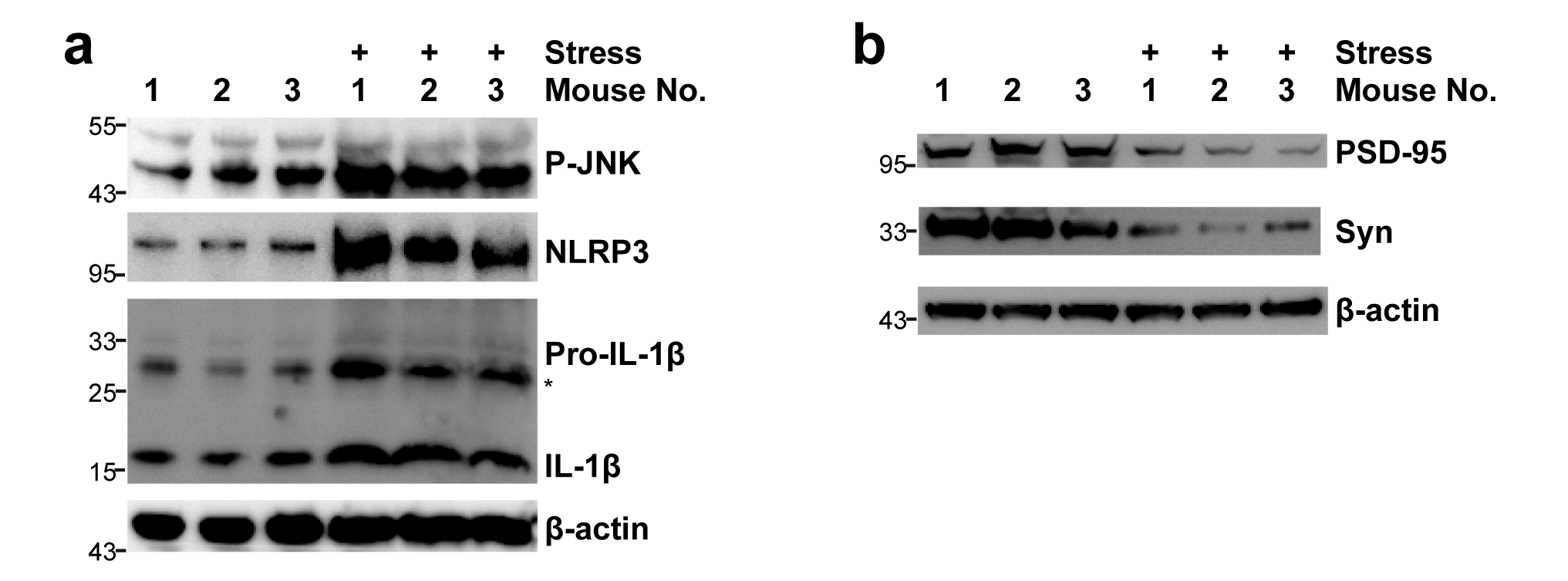


**Figure S3.** Effects of restraint stress on inflammasome activation and synaptic loss in the amygdala. a,b) Adult C57 BL/6 male mice were subjected to restraint from 0:00 a.m. to 8:00 a.m. each day for 8 consecutive days (stress group, *n* = 3) or placed in the home cage at the same time without food and water (control group, *n* = 3). Then all mice were sacrificed. Amygdala tissues were lysed in RIPA buffer, followed by IB analysis of inflammasome activation (a) and synaptic loss (b). *, nonspecific band; P-JNK, phospho-JNK at Thr183/Tyr185; Syn, Synaptophysin.

**
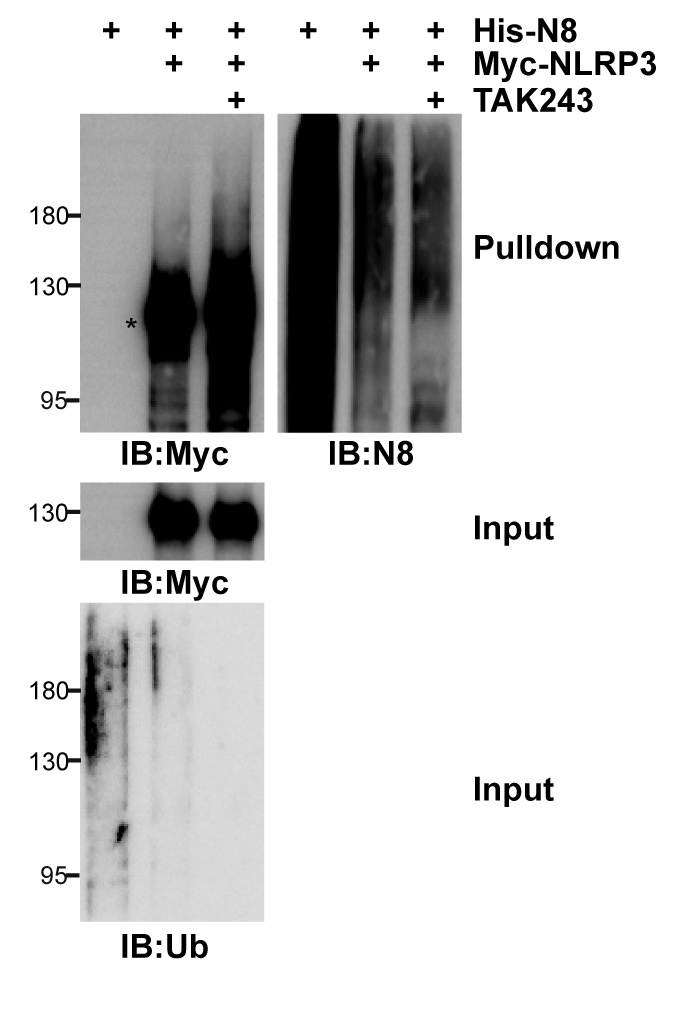
**

**Figure S4.** Effects of ubiquitination E1 UBA1 inhibitor TAK243 on NLRP3 neddylation. Twenty-four hours after transfection with His-NEDD8 and Myc-NLRP3, HEK-293T cells were treated with 1 μM TAK243 or DMSO of equal volume for 6 h. Myc-NLRP3 neddylation was examined by histidine pulldown. *, nonspecific band; Ub, ubiquitin; N8, NEDD8.


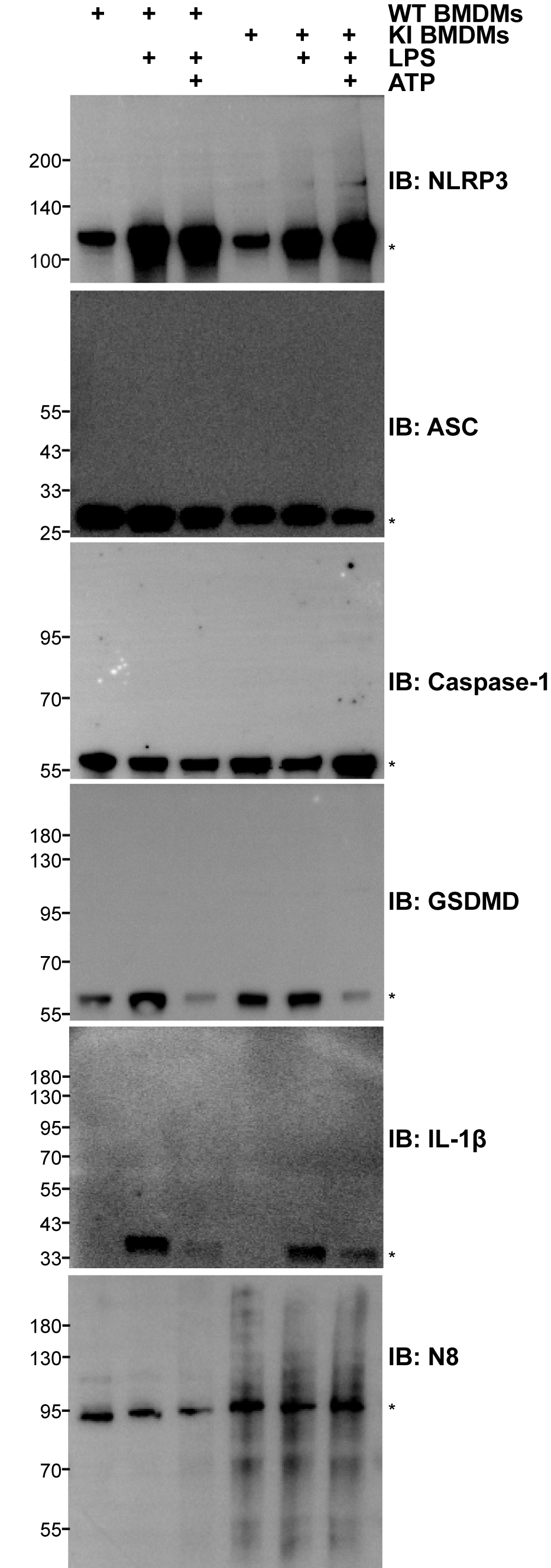


**Figure S5.** Screening possible neddylation substrate(s) in the NLRP3 inflammasome pathway. BMDMs from WT and 6 × His-FLAG-NEDD8 heterozygous KI mice were primed with 100 ng/mL LPS for 4 h, followed by treatment with 5 mM ATP for 30 min. The possible neddylation of endogenous NLRP3, ASC, pro-Caspase-1, GSDMD, pro-IL-1β was examined by histidine pulldown.


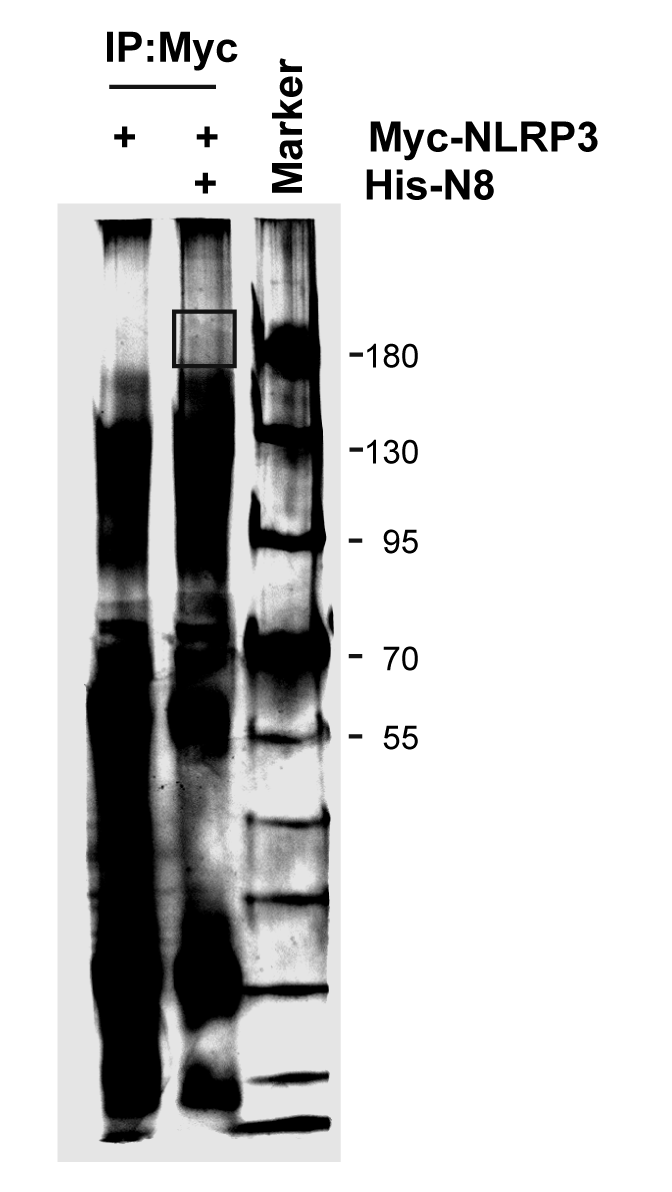


**Figure S6.** Preparation of the sample to detect neddylation site(s) by mass spectrometry. Twenty-four hours after transfection with the indicated mammalian expression vectors, HEK-293T cells were subjected to IP with an anti-Myc antibody under partially denaturing conditions. Precipitates were then subjected to SDS-PAGE, followed by silver staining. Smear bands in the indicated regions were cut and subjected to mass spectrometry.


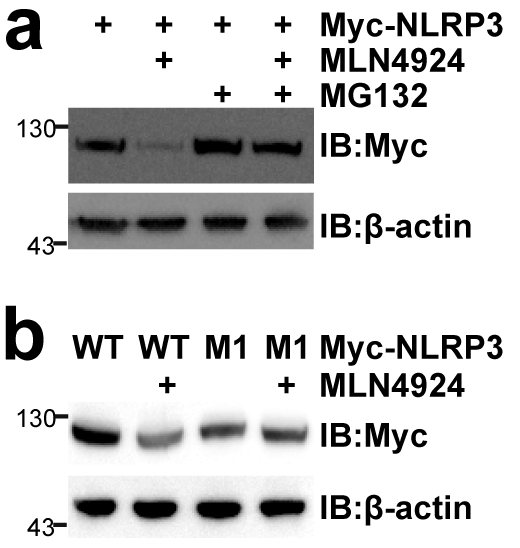


**Figure S7.** NLRP3 neddylation prevents its Ub-proteasome–dependent degradation. a,b) Twenty-four hours after transfection with the indicated plasmids, HEK-293T cells were treated with or without MLN4924 (0.2 μM, 12 h) and MG132 (20 μM, 6 h) as indicated. The expression of Myc-NLRP3 and β-actin was analyzed by IB.


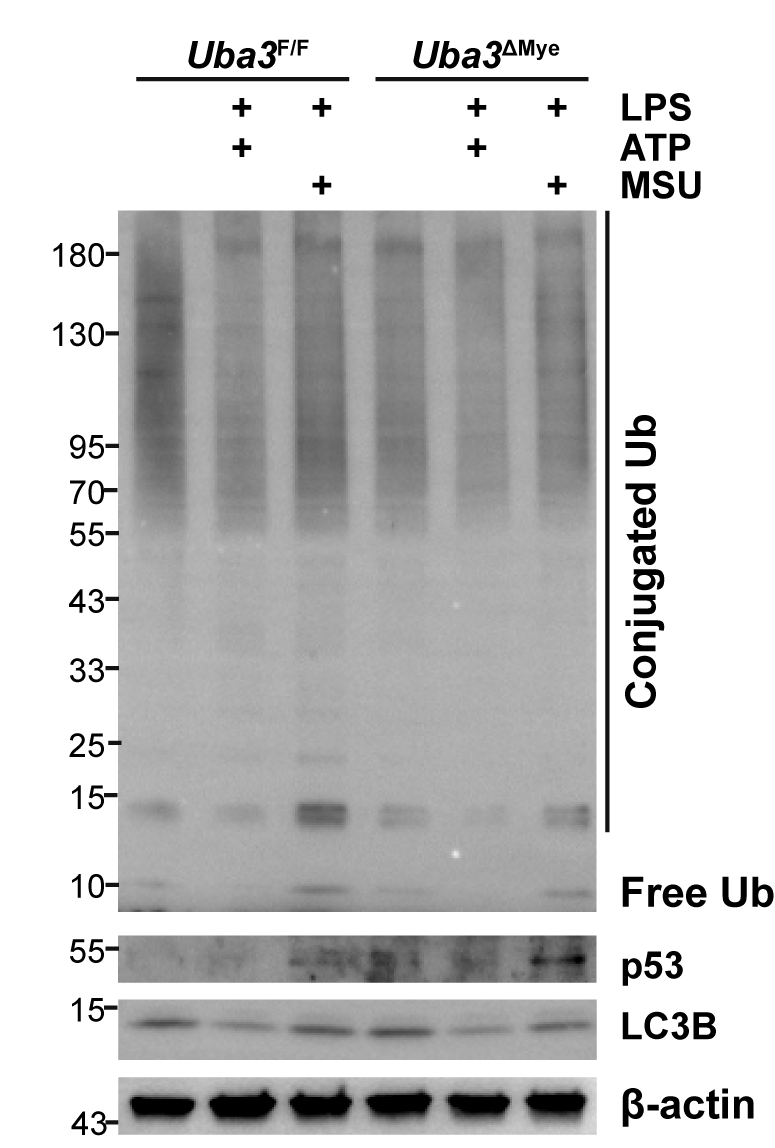


**Figure S8.** The global ubiquitination level in UBA3-sufficient and -deficient BMDMs before and after NLRP3 inflammasome activation. BMDMs from *Uba3*^F/F^ and *Uba3*^ΔMye^ mice were primed with 100 ng/mL LPS for 4 h, followed by treatment with 5 mM ATP for 30 min or 200 ng/mL MSU for 6 h. Whole cell lysates were harvested and subjected to IB with the indicated antibodies. Ub, ubiquitin.


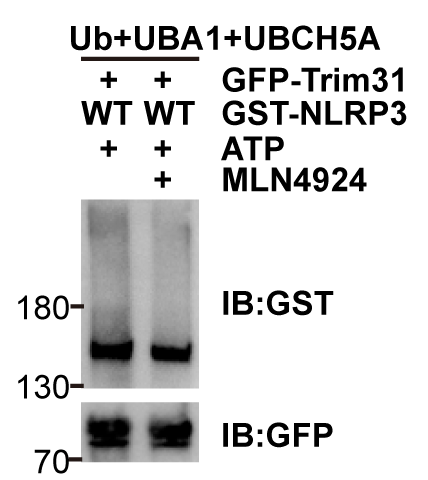


**Figure S9.** Effects of neddylation blockade on the ubiquitination E3 ligase activity of Trim31. Twenty-four hours after transfection with a plasmid encoding GFP-Trim31, HEK-293T cells were treated with 0.2 μM MLN4924 or DMSO of equal volume for 12 h. GFP-Trim31 was then immunoprecipitated with an antibody against GFP, followed by incubation with purified GST-NLRP3 WT, Ub, E1 UBA1, and E2 UBCH5A in the presence of ATP for 1 h. The samples were then subjected to IB with antibodies against GST and GFP.


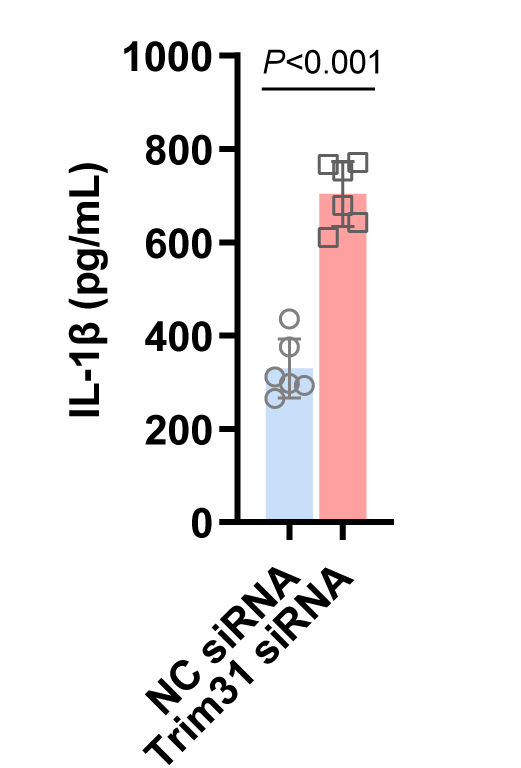


**Figure S10.** Effects of Trim31 knockdown on LPS + ATP-triggered IL-1β secretion from UBA3-deficient BMDMs. The supernatants harvested from BMDMs (*n* = 6/group) in Figure 6m,n with LPS + ATP treatment were subjected to IL-1β ELISA. Error bars show mean ± SD. *P* values were determined by two-tailed Student’s *t* test.
