# Supplementary material for: Neddylation Targets and Stabilizes NLRP3 to Augment Inflammasome‐Mediated Colitis and Mood Disorder
Source: Adv Sci (Weinh). 2026 Jan 9;13(16):e05906. doi: 10.1002/advs.202505906 (PMC13042396; doi:10.1002/advs.202505906)
Supplement: Supplementary file 2 — Supporting File 2: advs73747‐sup‐0002‐Data.zip. [file ADVS-13-e05906-s001.zip › advs73747-sup-0002-Data/uncroppedblots.pdf]

Source Figure 1c

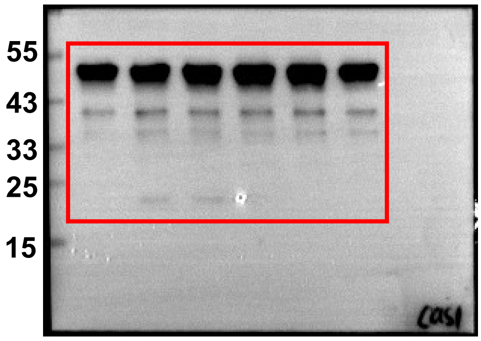

IB:Caspase-1

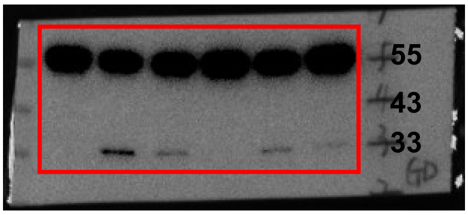

IB:GSDMD

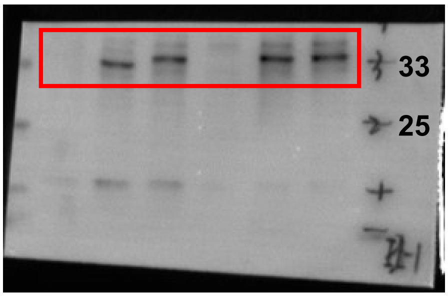

IB:IL-1β

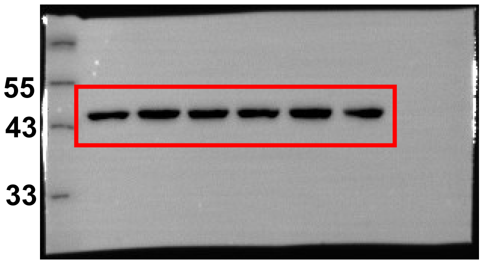

IB:β-actin

Source Figure 1f

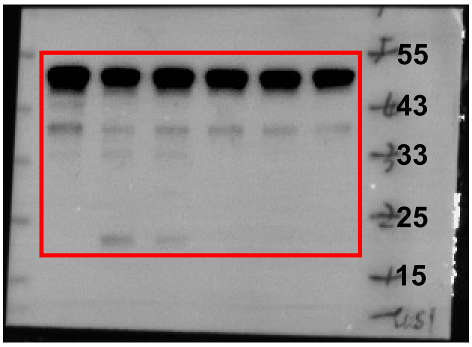

IB:Caspase-1

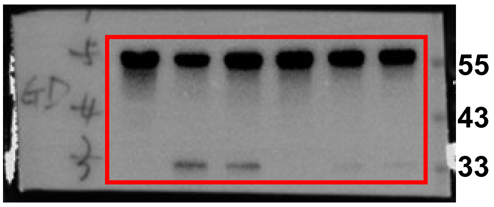

IB:GSDMD

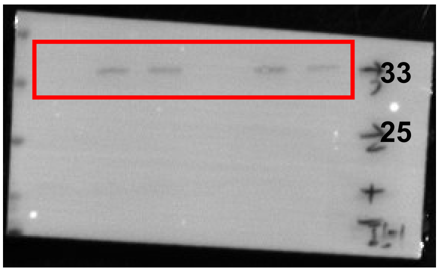

IB:IL-1β

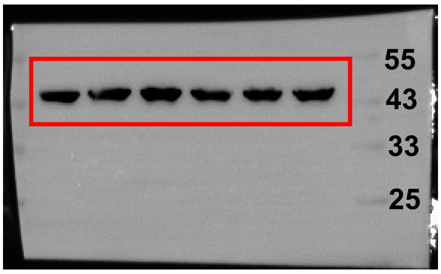

IB:β-actin

Source Figure 2i

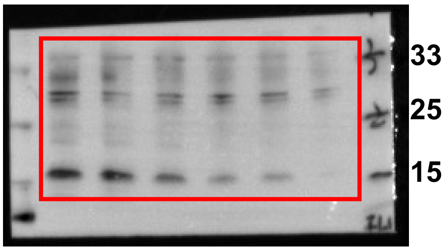

IB:IL-1β

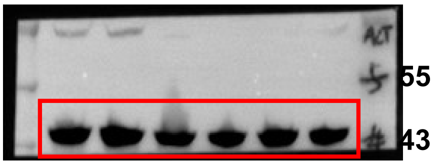

IB:β-actin

Source Figure 3f

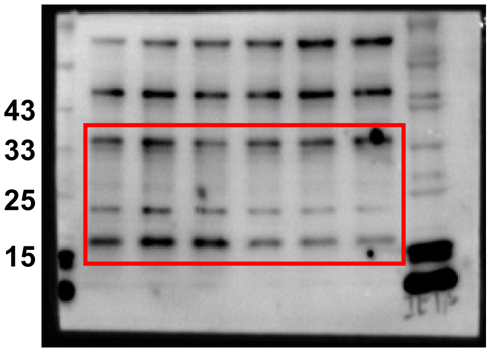

IB:IL-1β

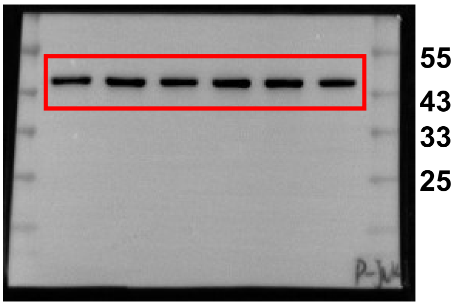

IB:β-actin

Source Figure 3h

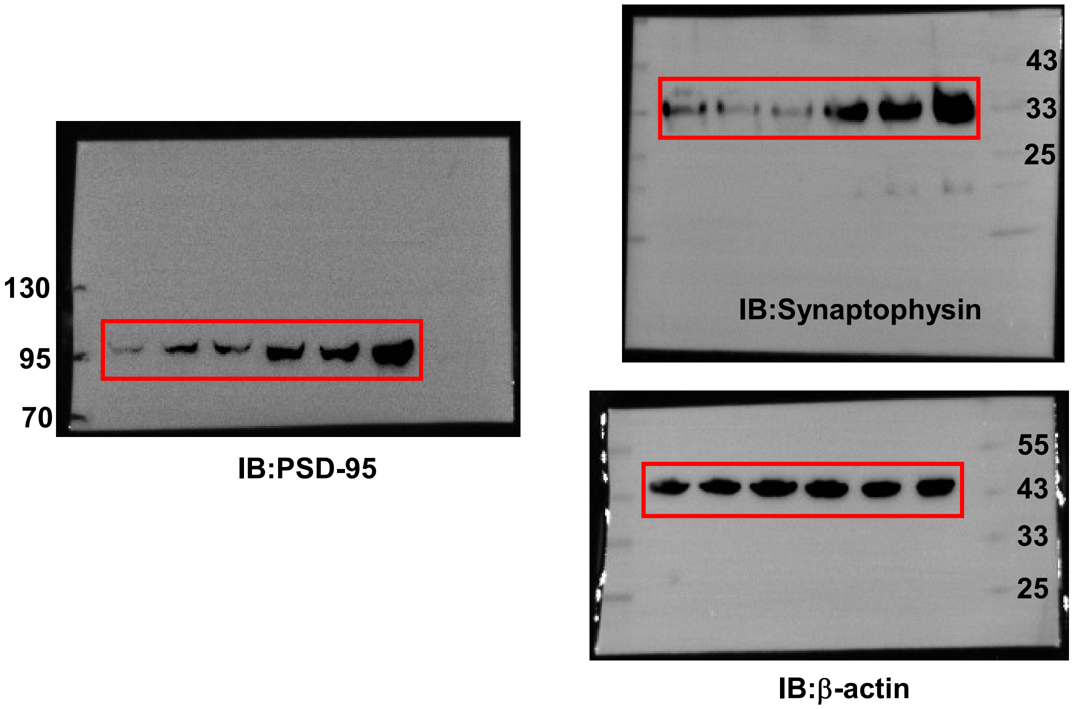

Source Figure 4b

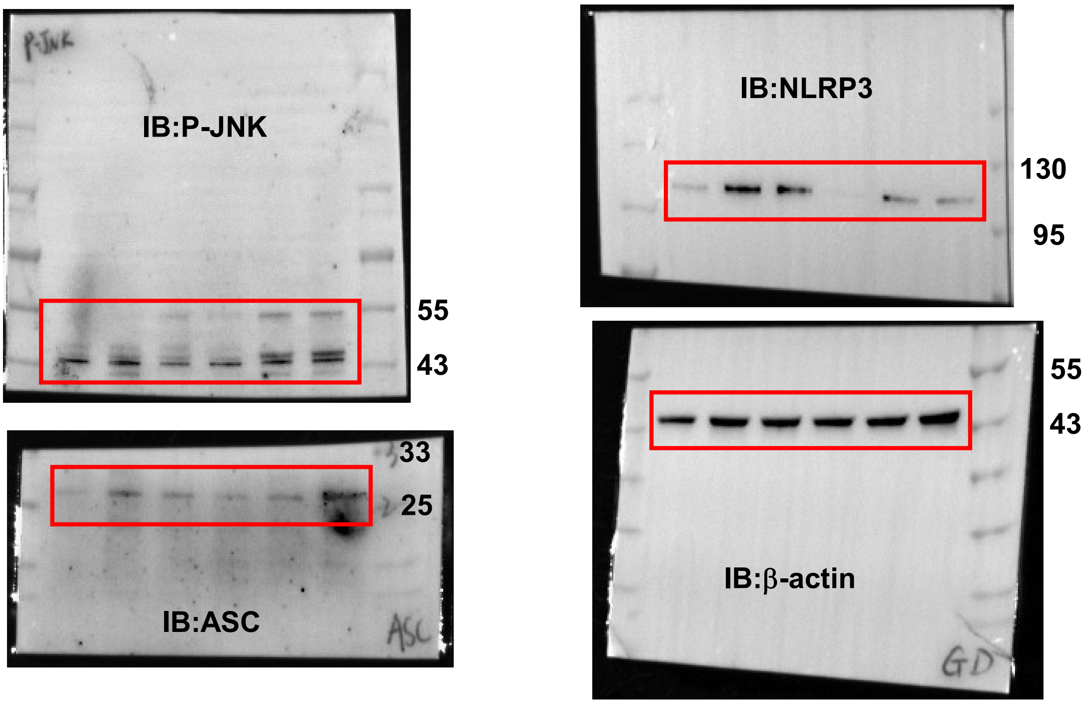

Source Figure 4d

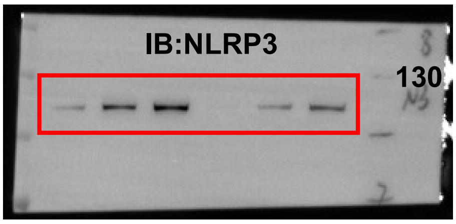

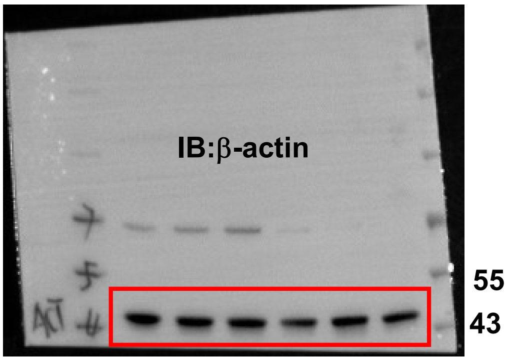

Source Figure 4f

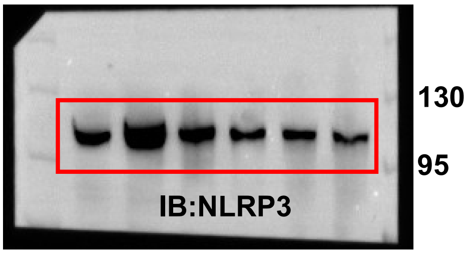

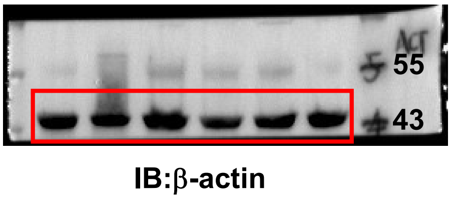

Source Figure 4i

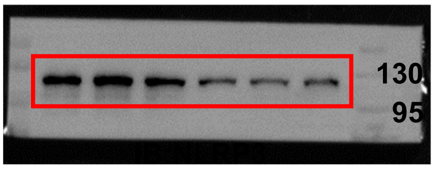

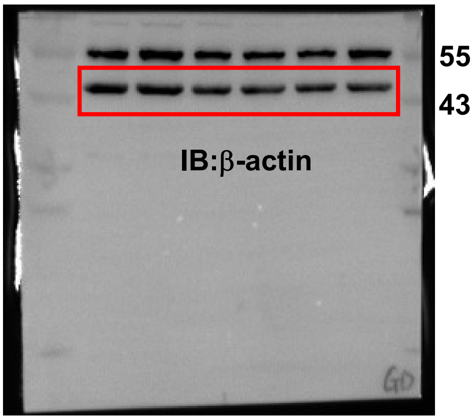

Source Figure 5a

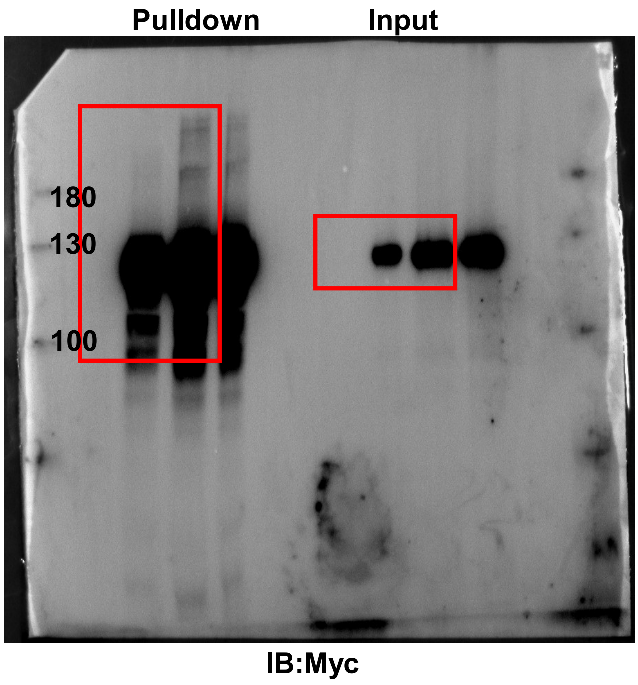

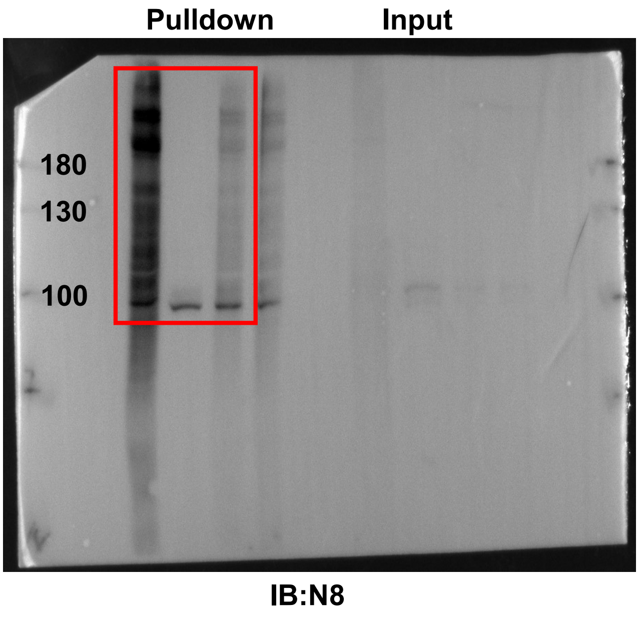

Source Figure 5b

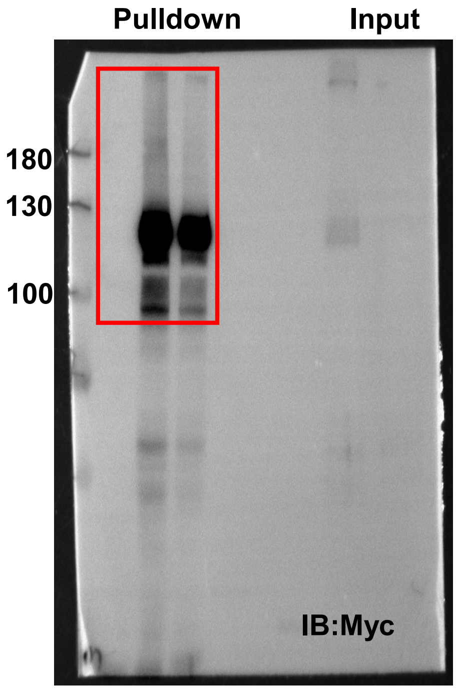

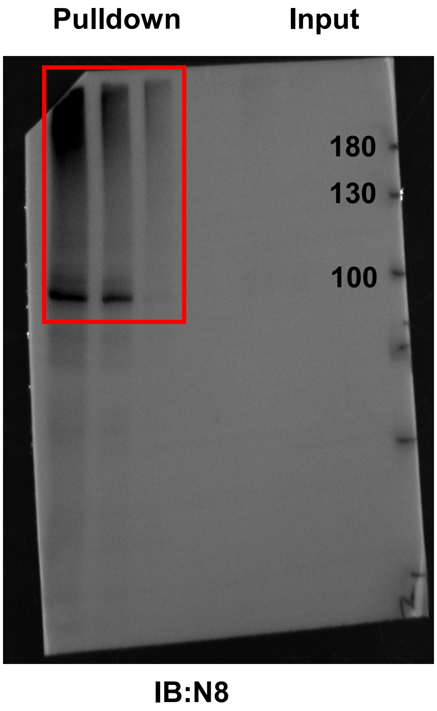

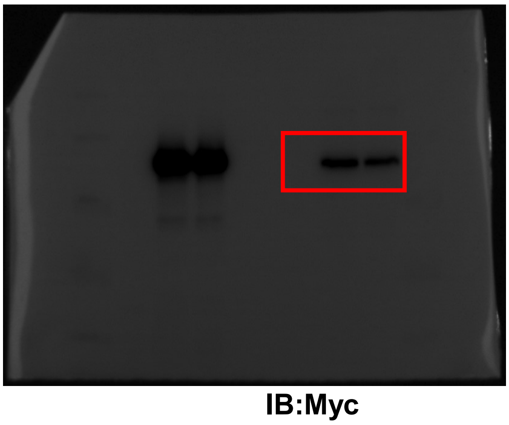

Source Figure 5c

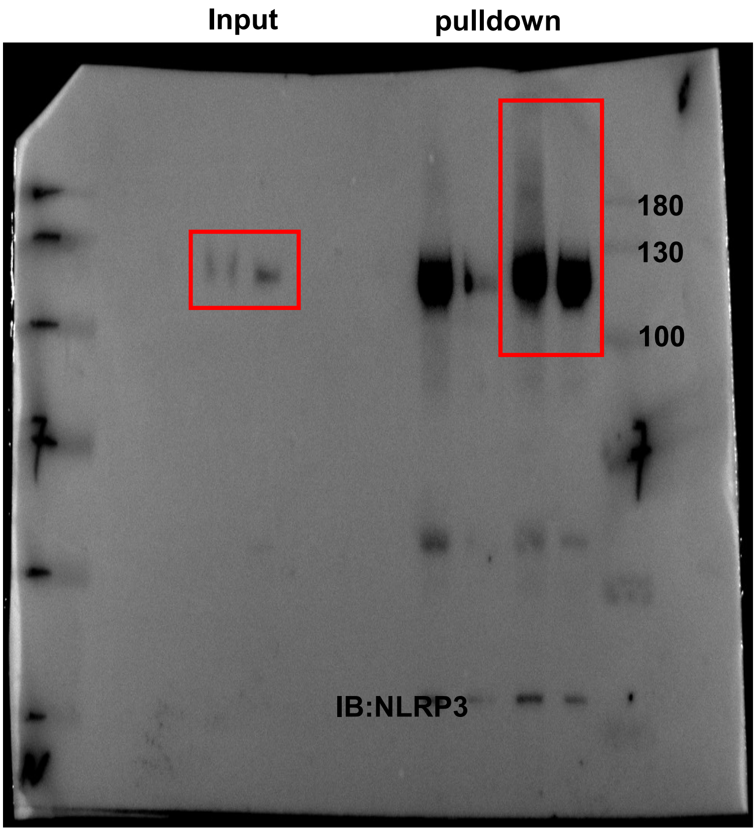

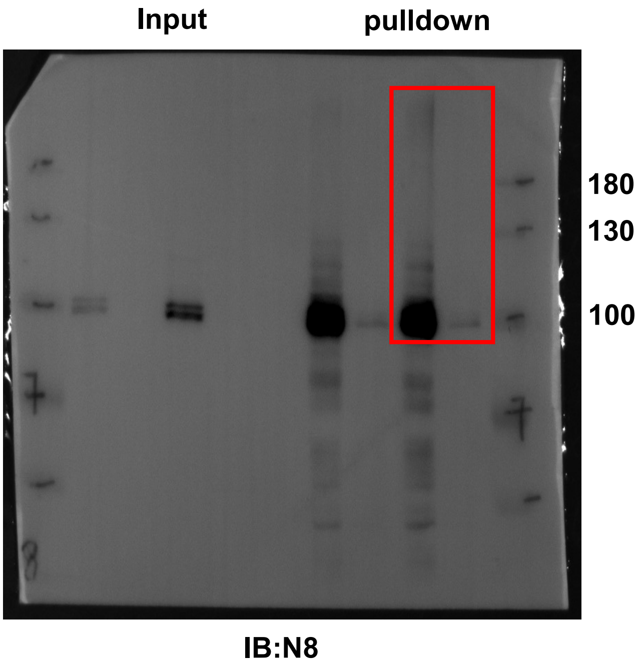

Source Figure 5e

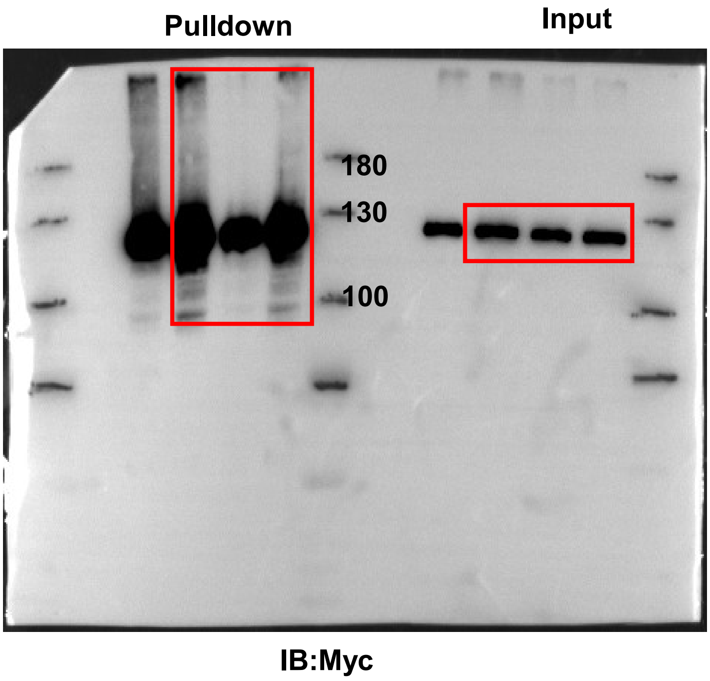

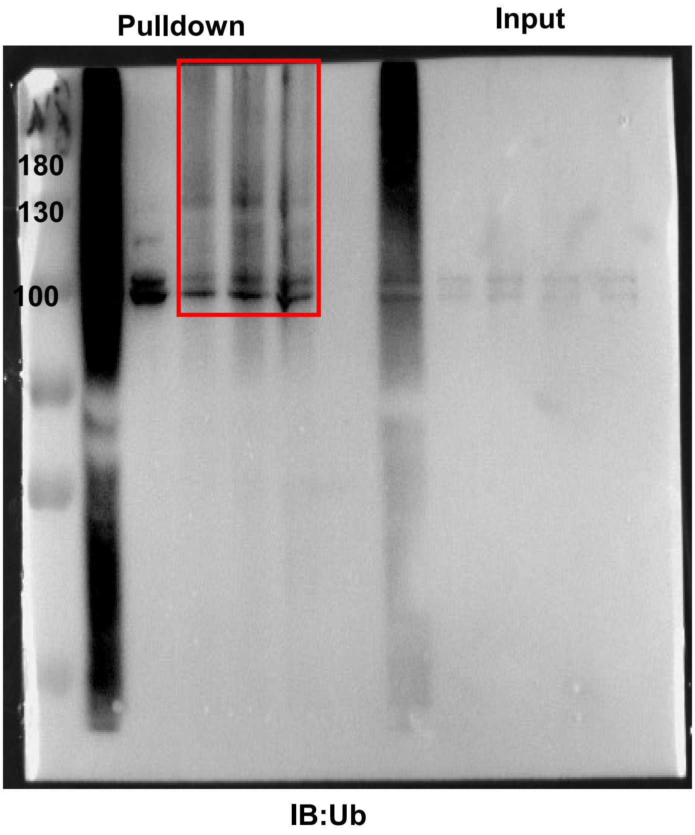

Source Figure 5f

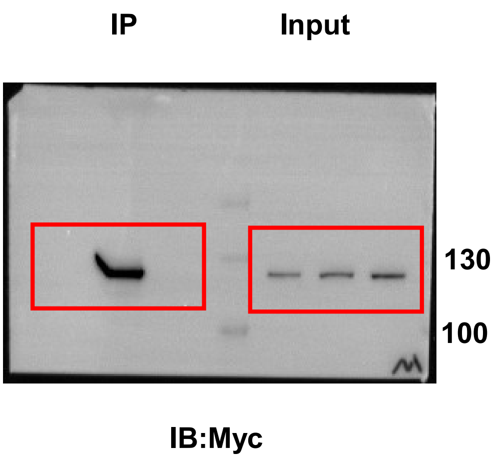

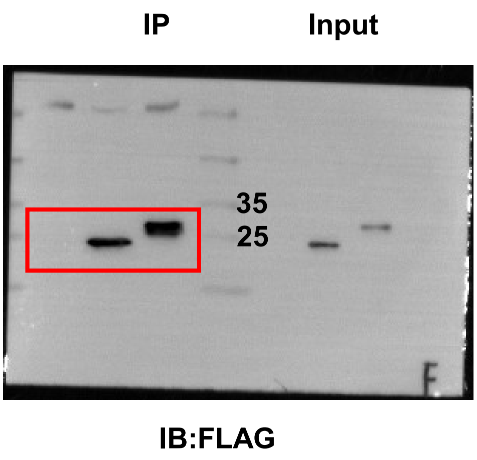

Source Figure 5g

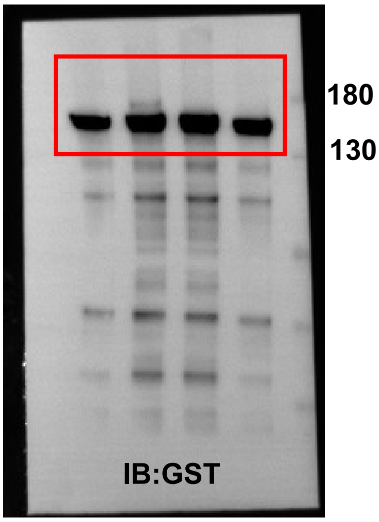

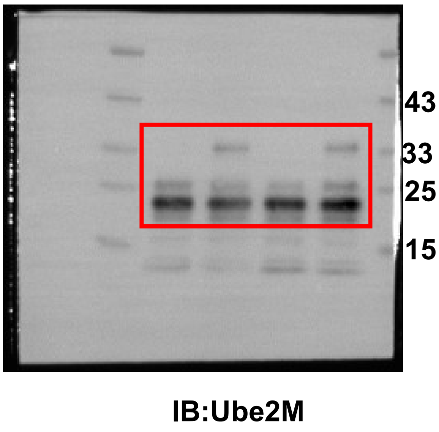

Source Figure 5h

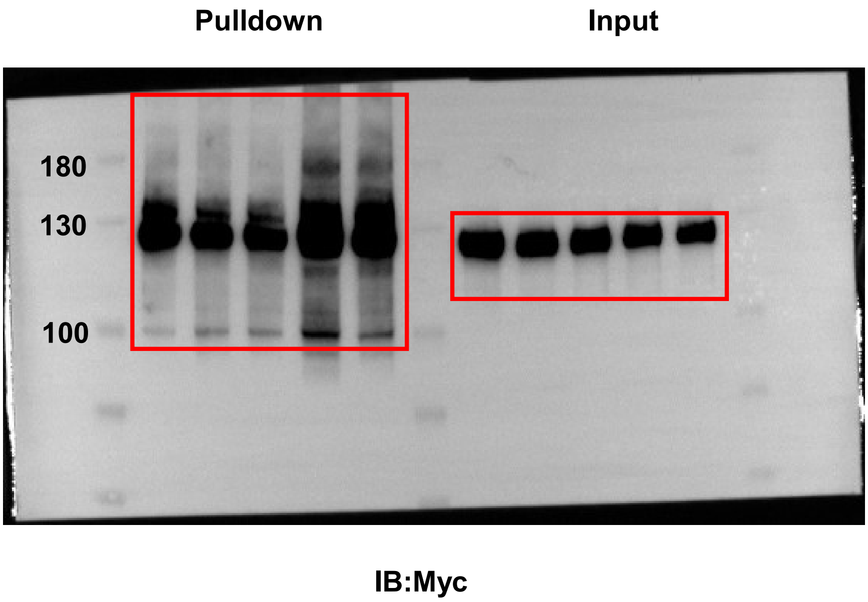

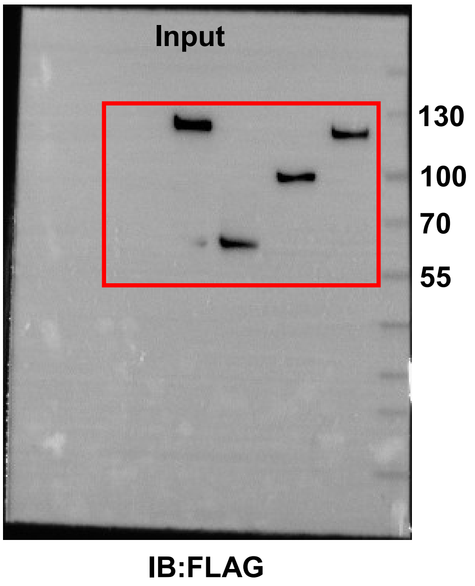

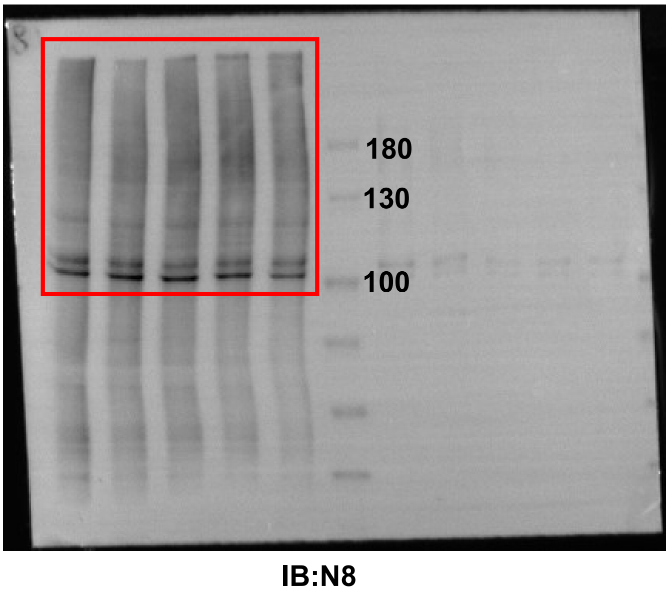

Source Figure 5i

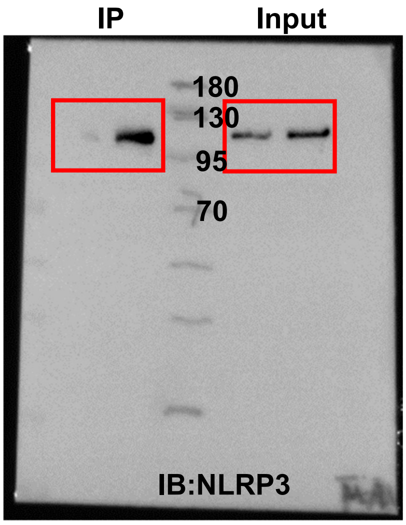

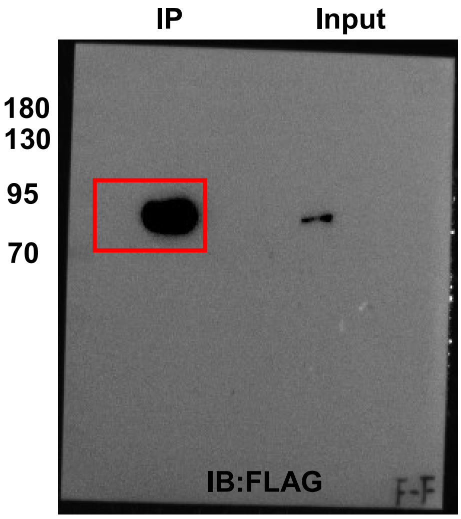

Source Figure 5j

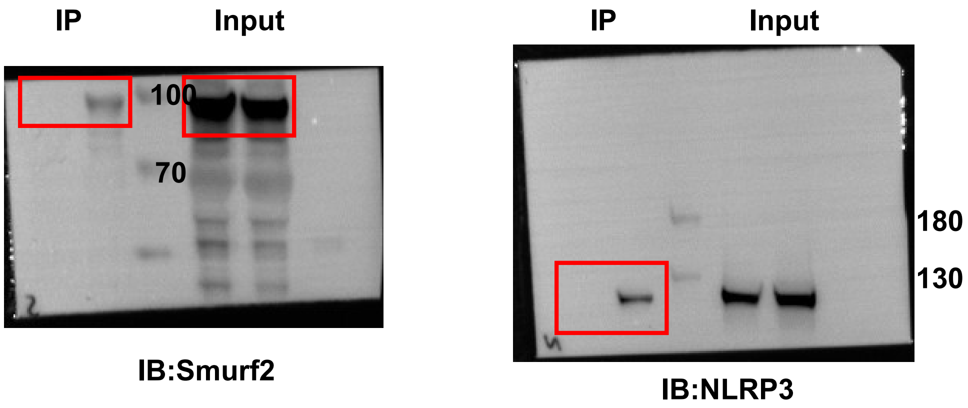

Source Figure 5k

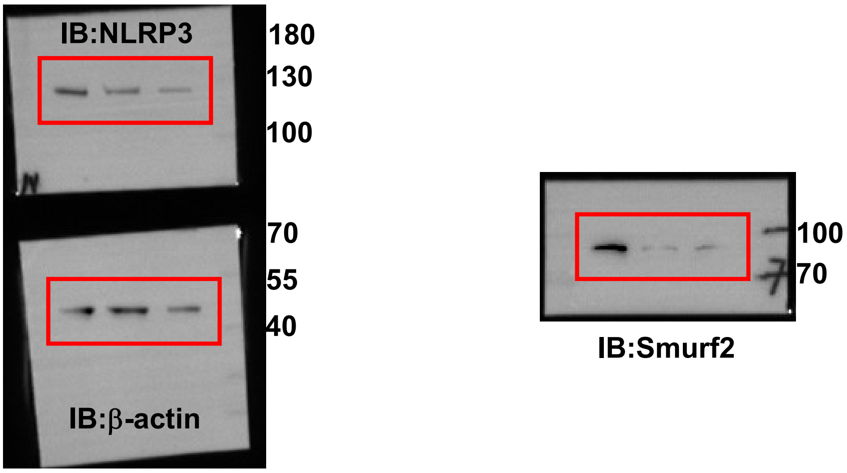

Source Figure 5l

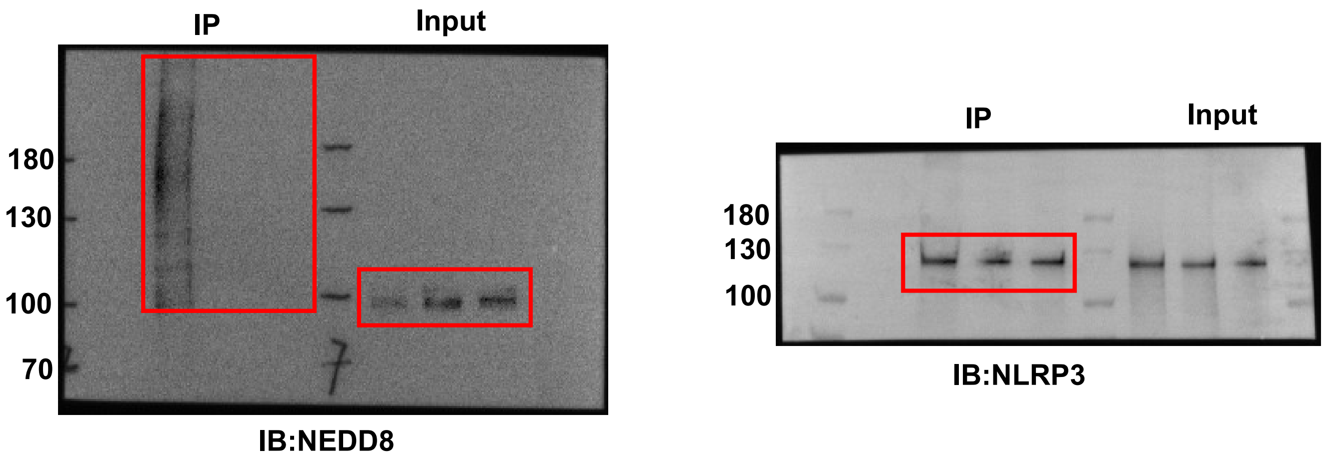

Source Figure 5m

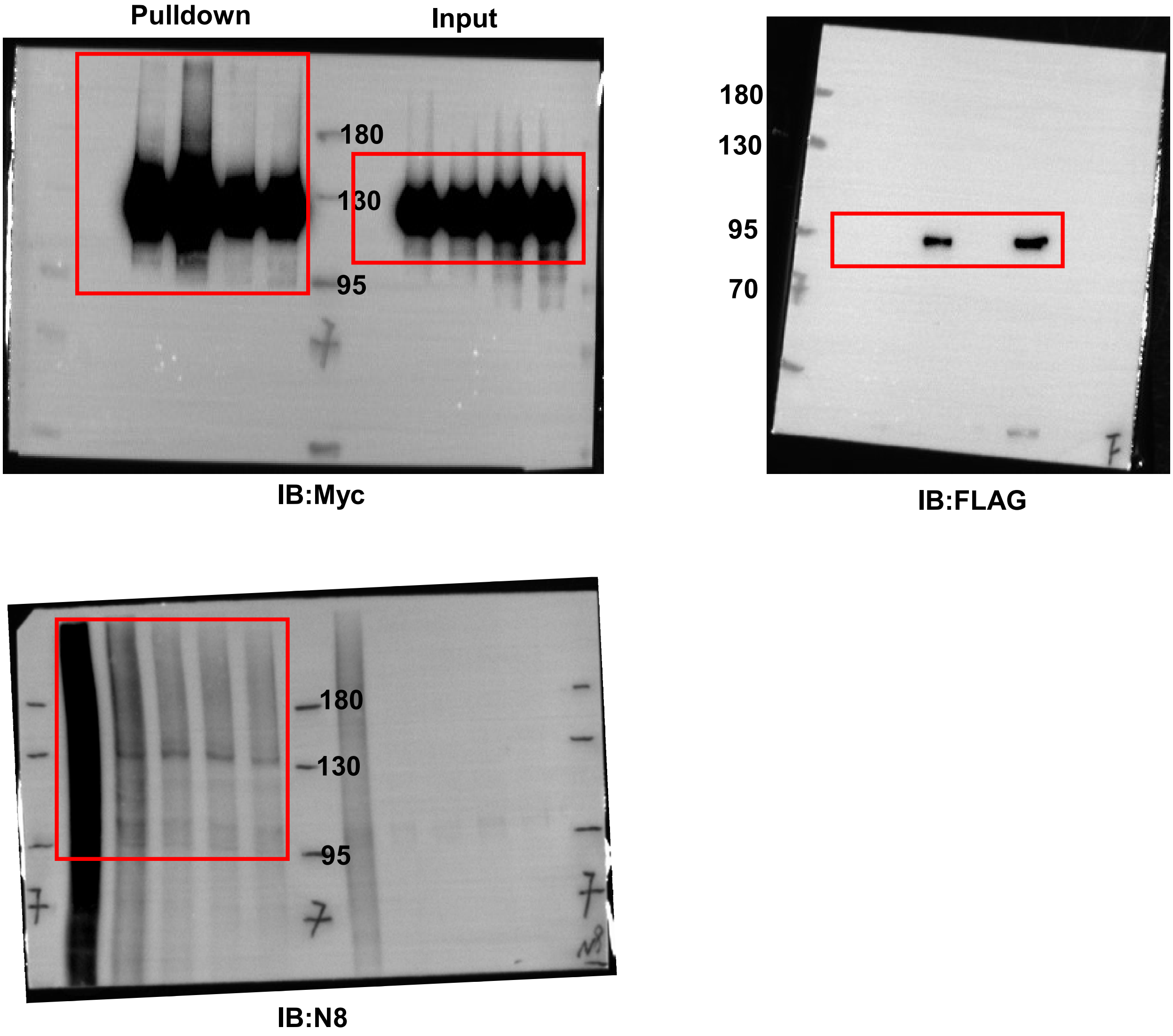

Source Figure 6a

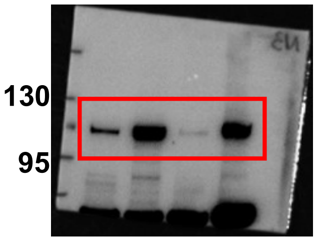

IB:NLRP3

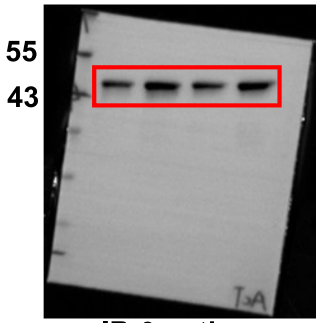

IB:β-actin

Source Figure 6b

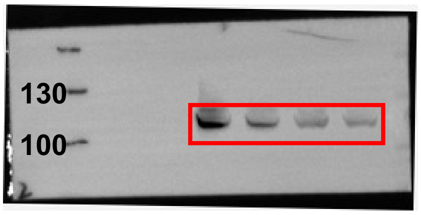

IB:NLRP3

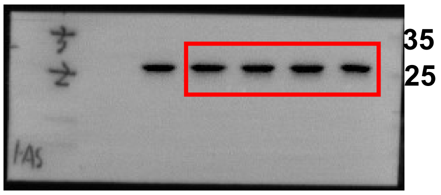

IB:ASC

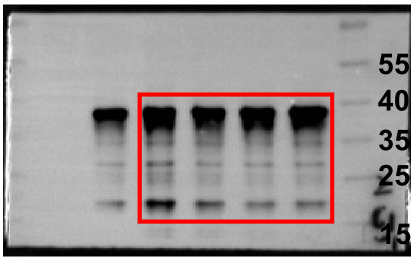

IB:Caspase-1

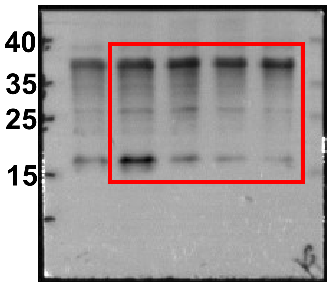

IB:IL-1β

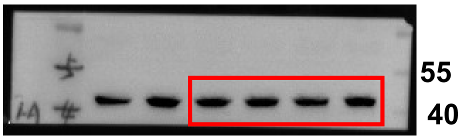

IB:β-actin

Source Figure 6d

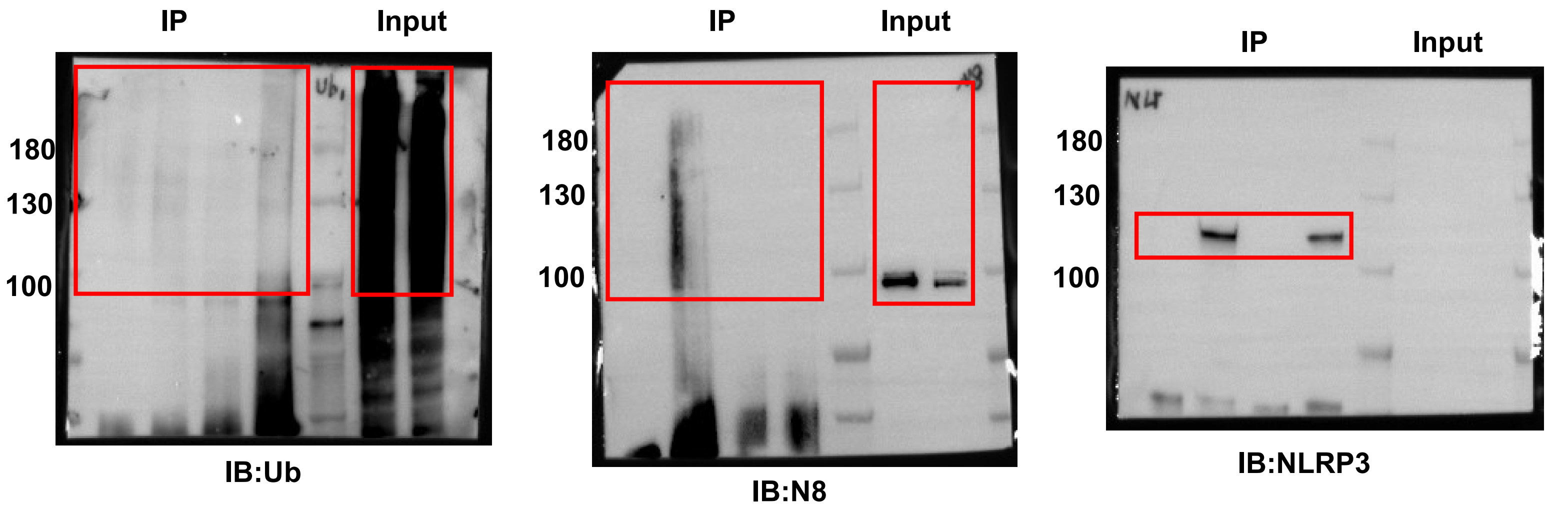

Source Figure 6e

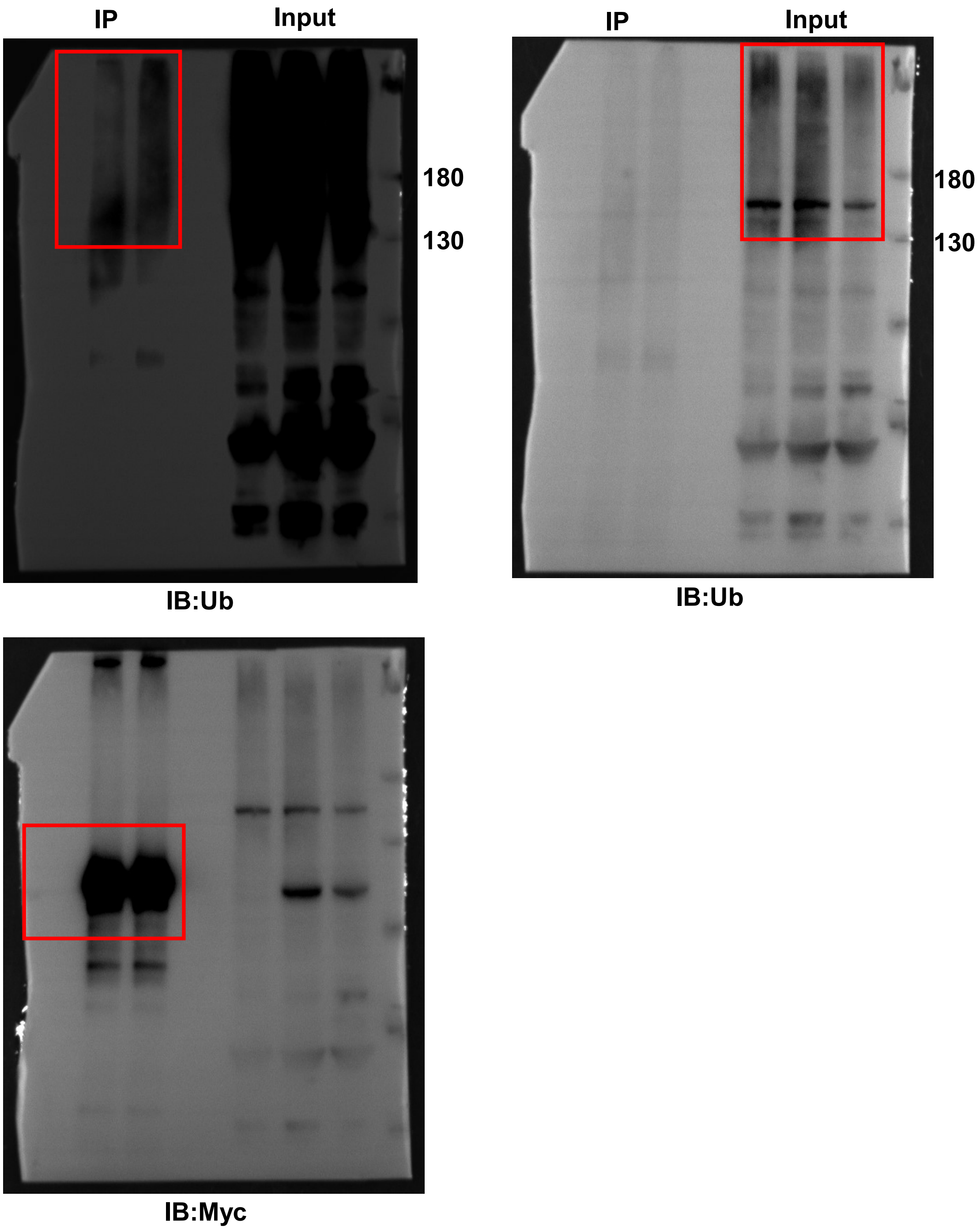

Source Figure 6f

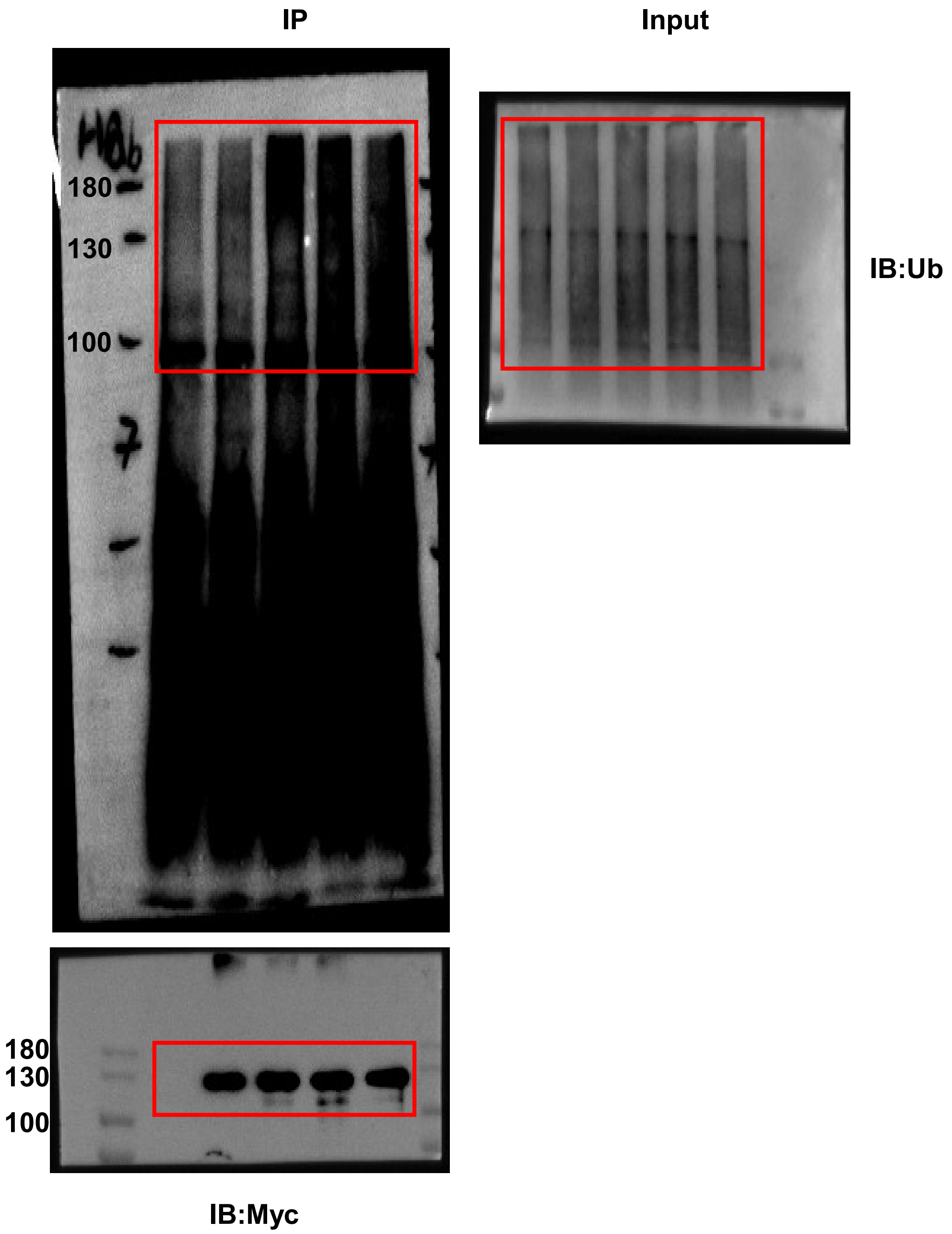

Source Figure 6g

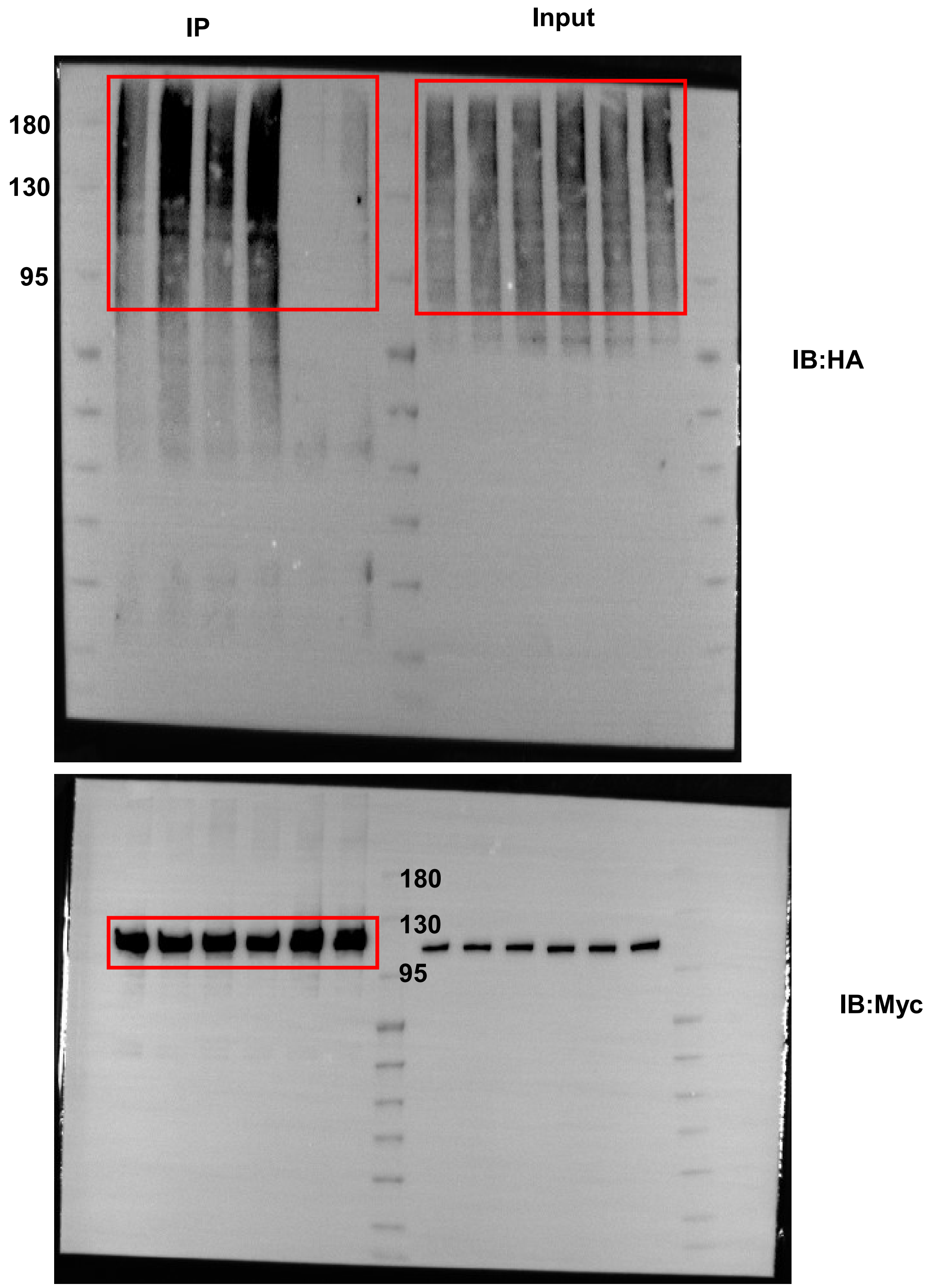

Source Figure 6h

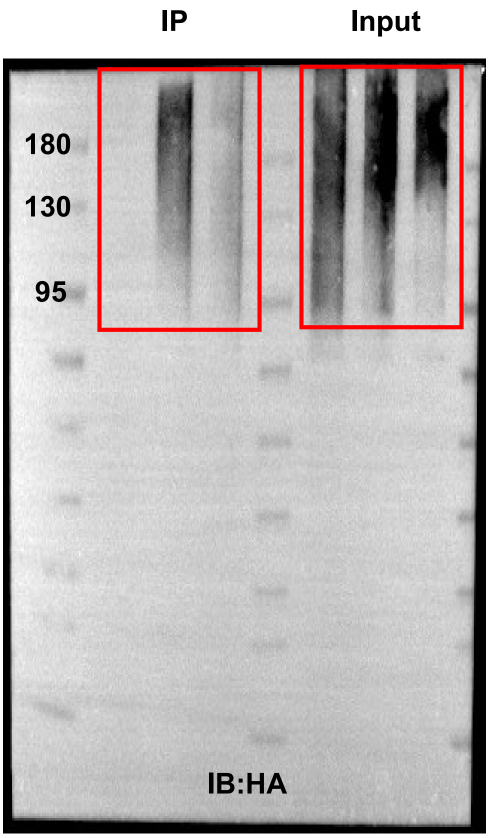

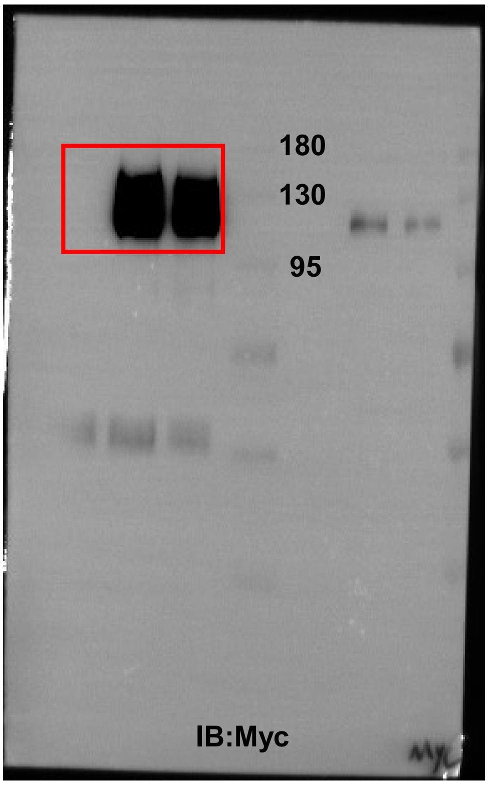

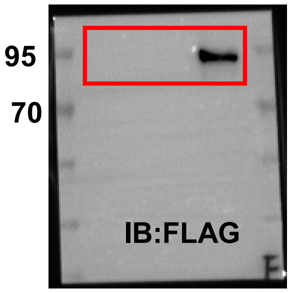

Source Figure 6i

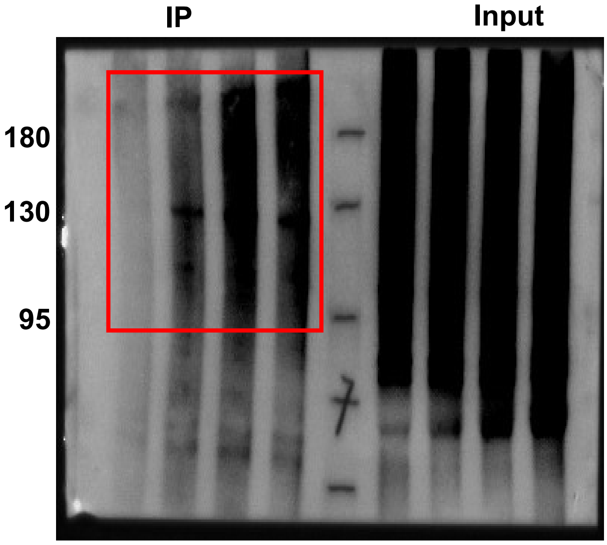

IB:HA long exposure

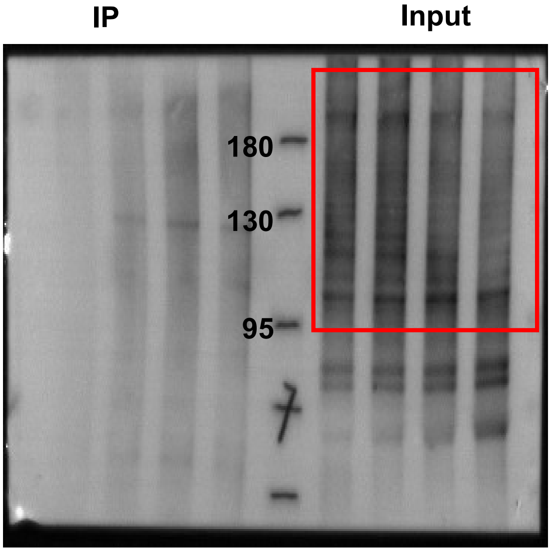

IB:HA short exposure

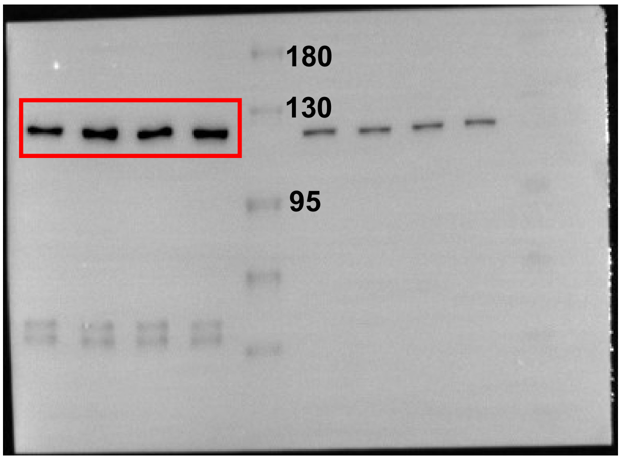

IB:Myc

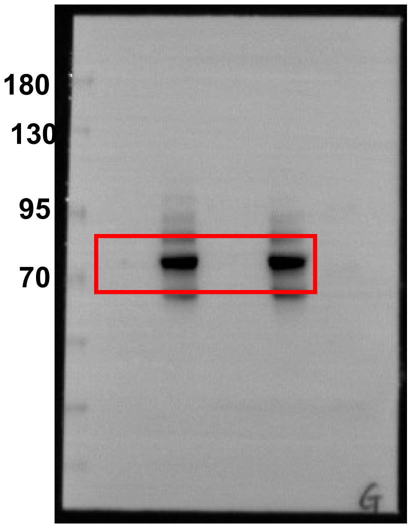

IB:GFP

Source Figure 6j

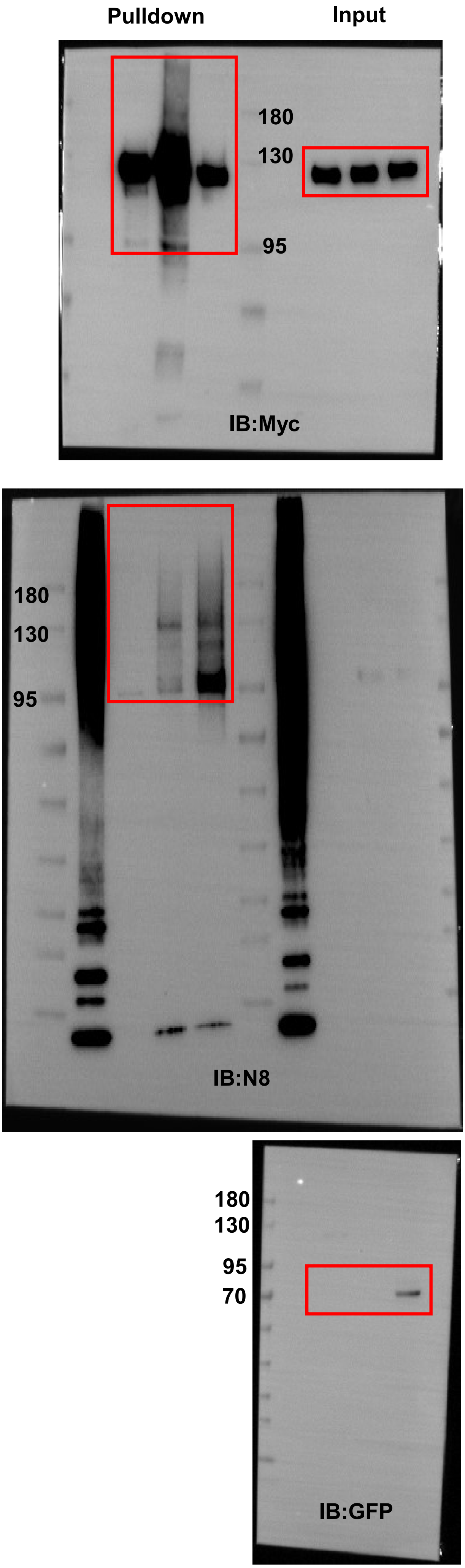

Source Figure 6k

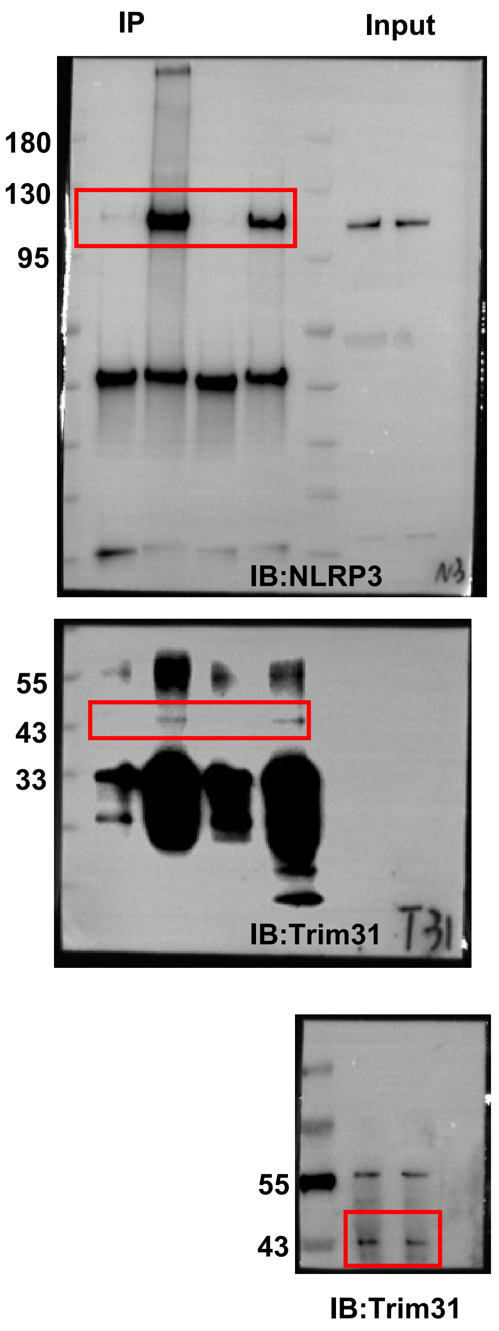

Source Figure 6l

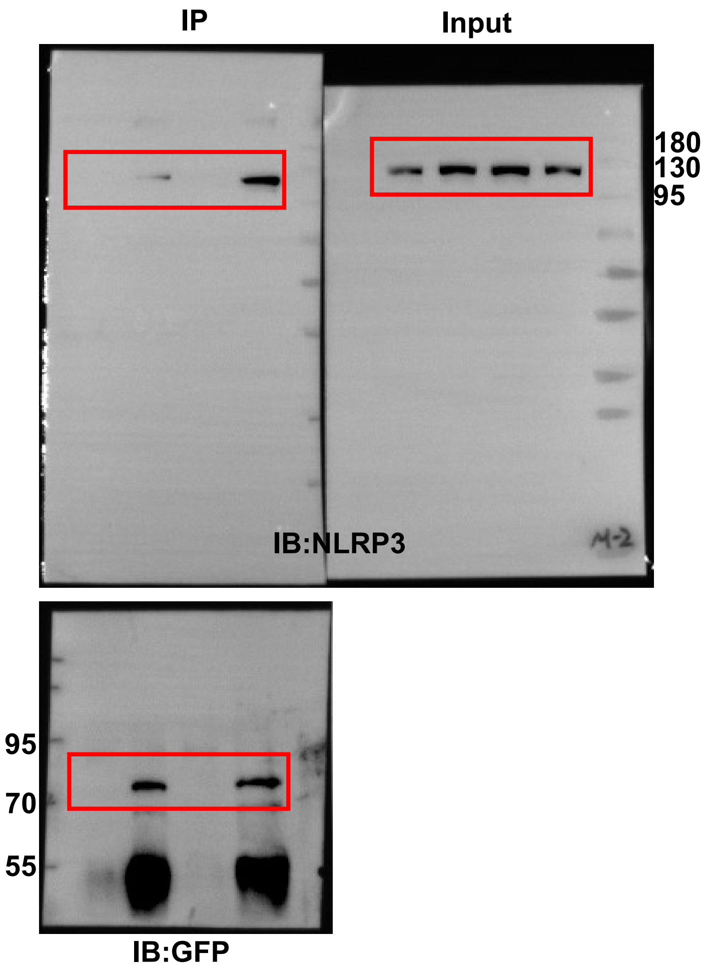

Source Figure 6m

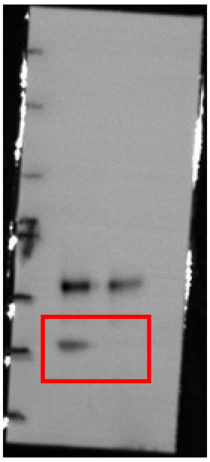

IB:Trim31

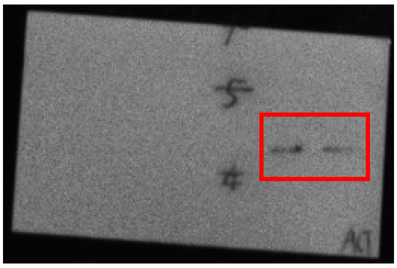

IB:β-actin

Source Figure 6n

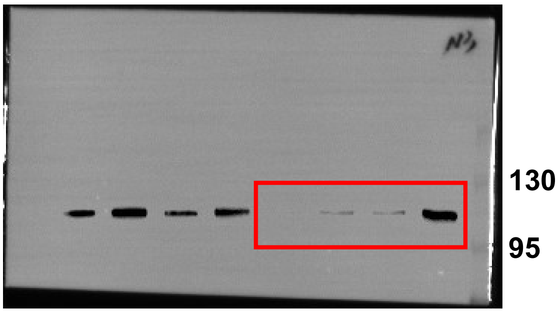

IB:NLRP3

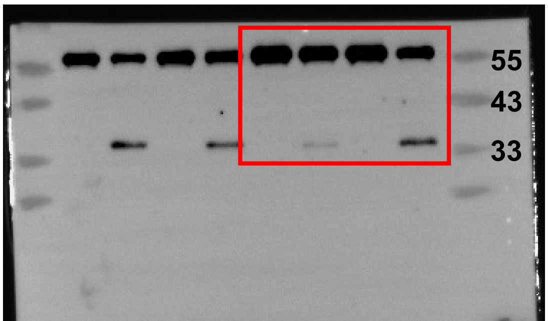

IB:GSDMD

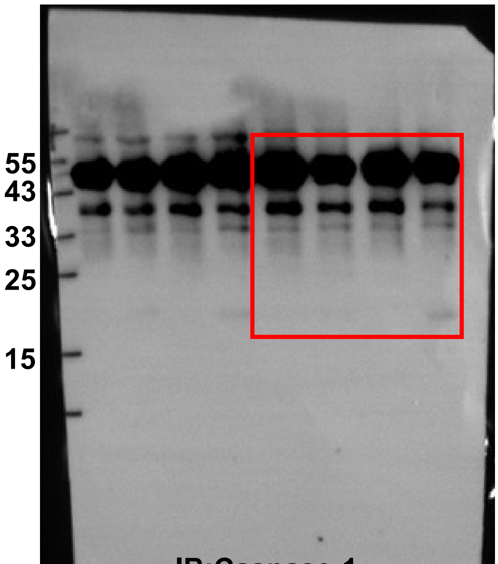

IB:Caspase-1

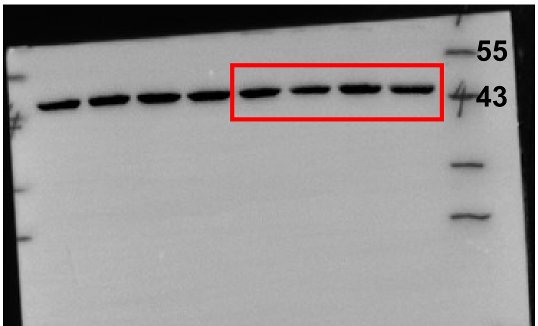

IB:β-actin

Source Figure 7f

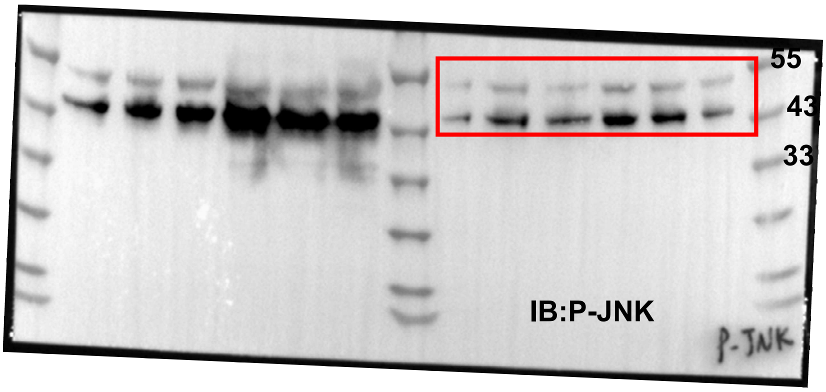

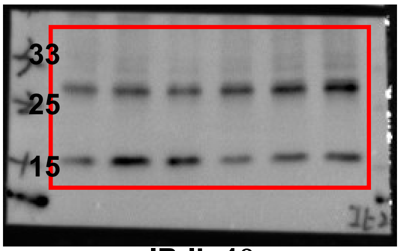

IB:IL-1β

130
95

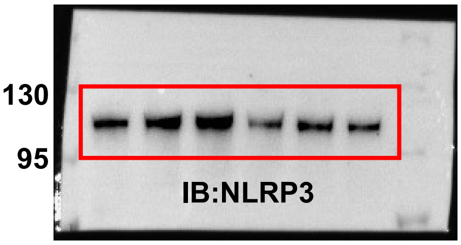

55
43

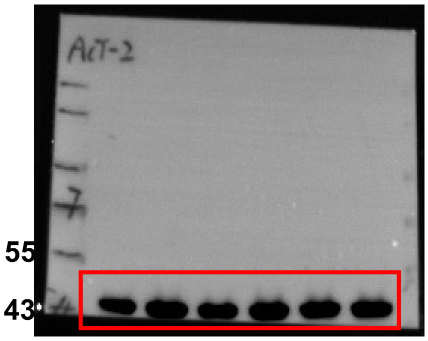

IB:β-actin

Source Figure 7h

130
95

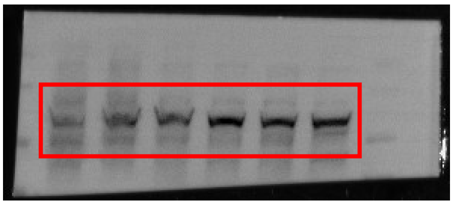

IB:PSD-95

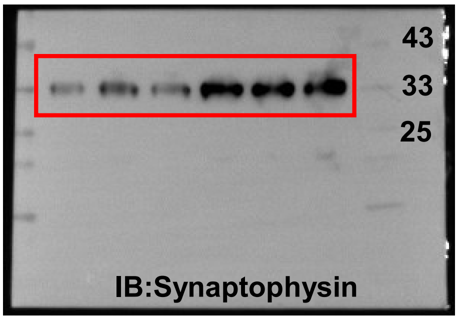

55
43

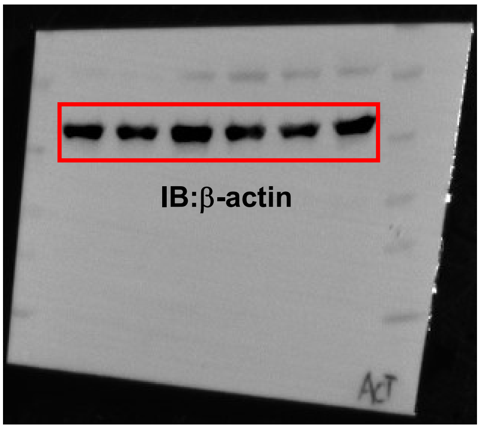

Source Figure S1a

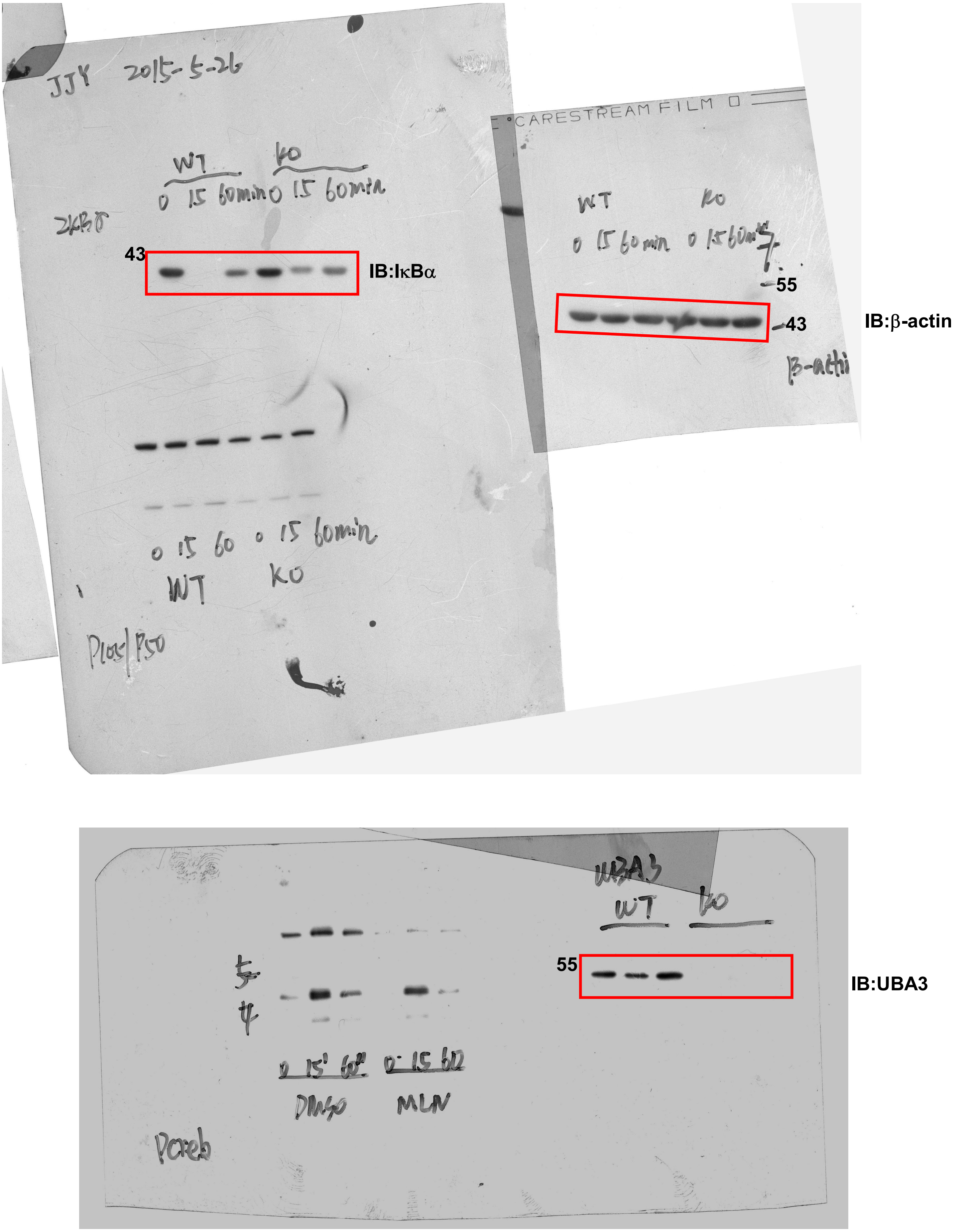

Source Figure S2

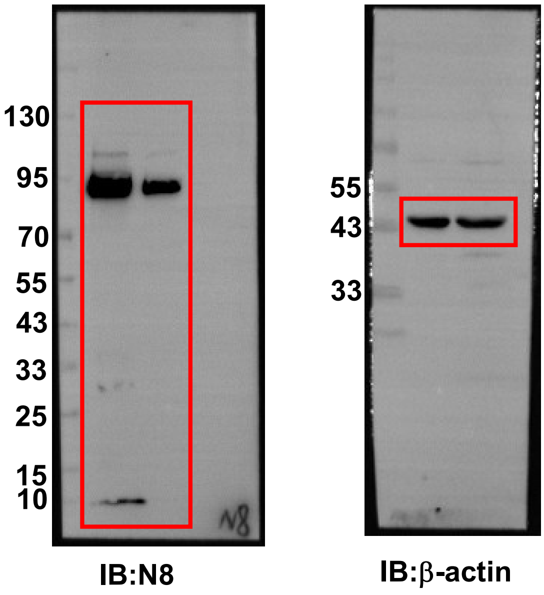

Source Figure S3a

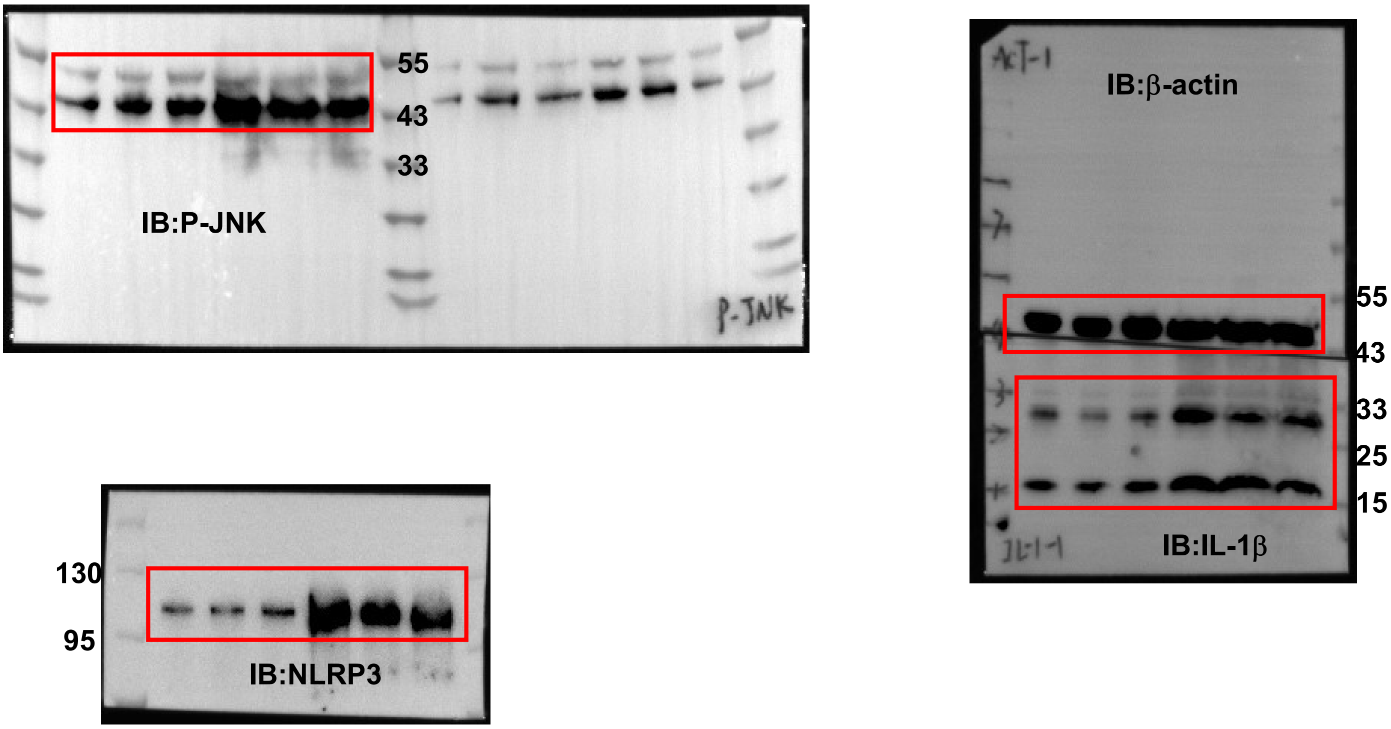

Source Figure S3b

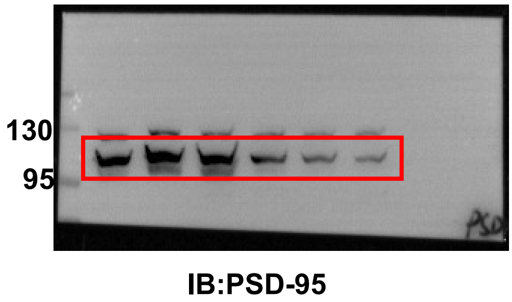

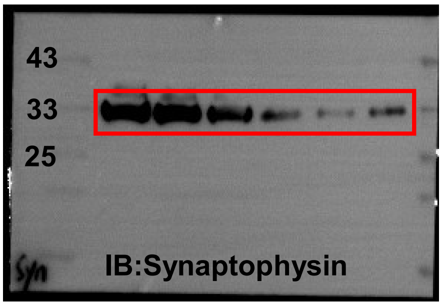

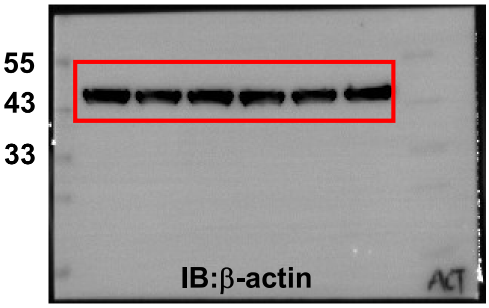

Source Figure S4

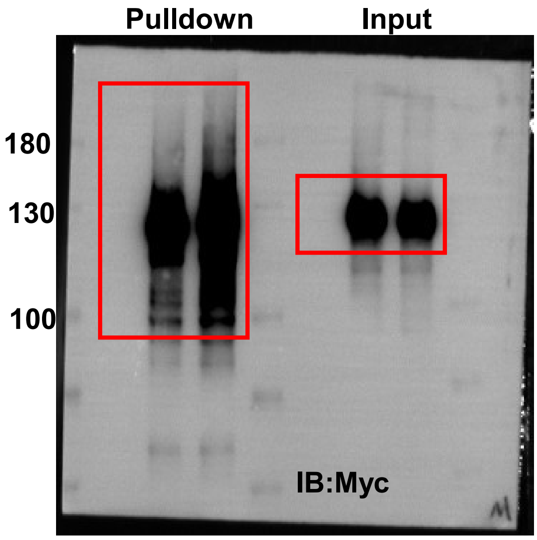

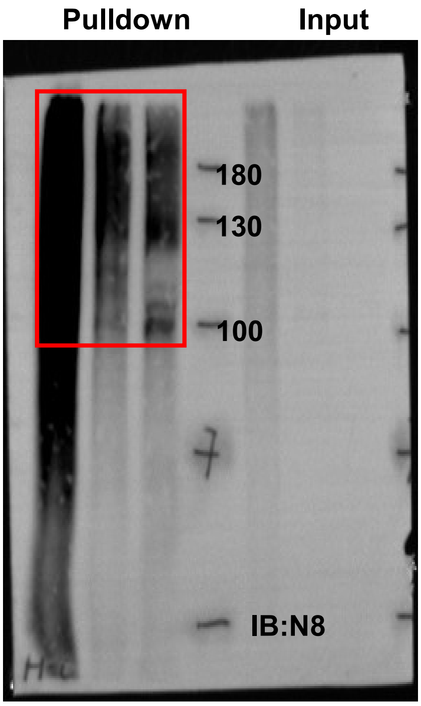

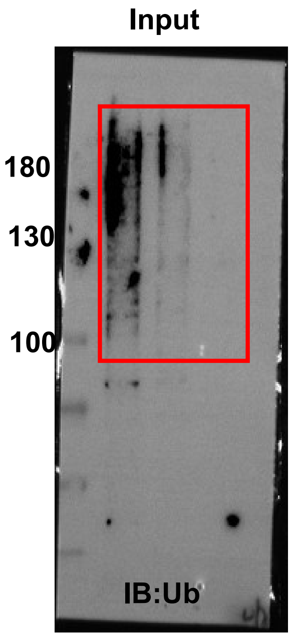

Source Fig. S5

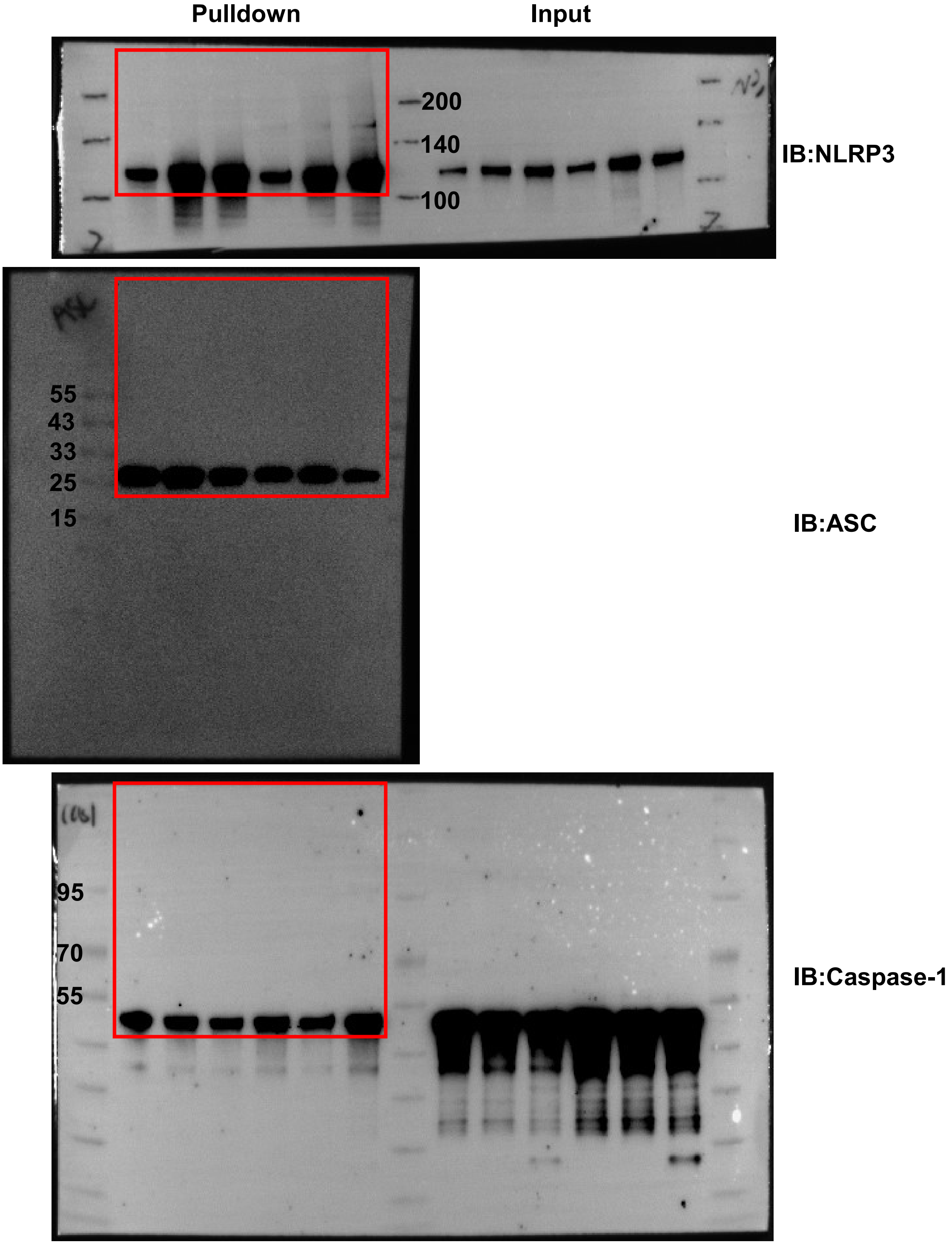

Source Fig. S5 continued

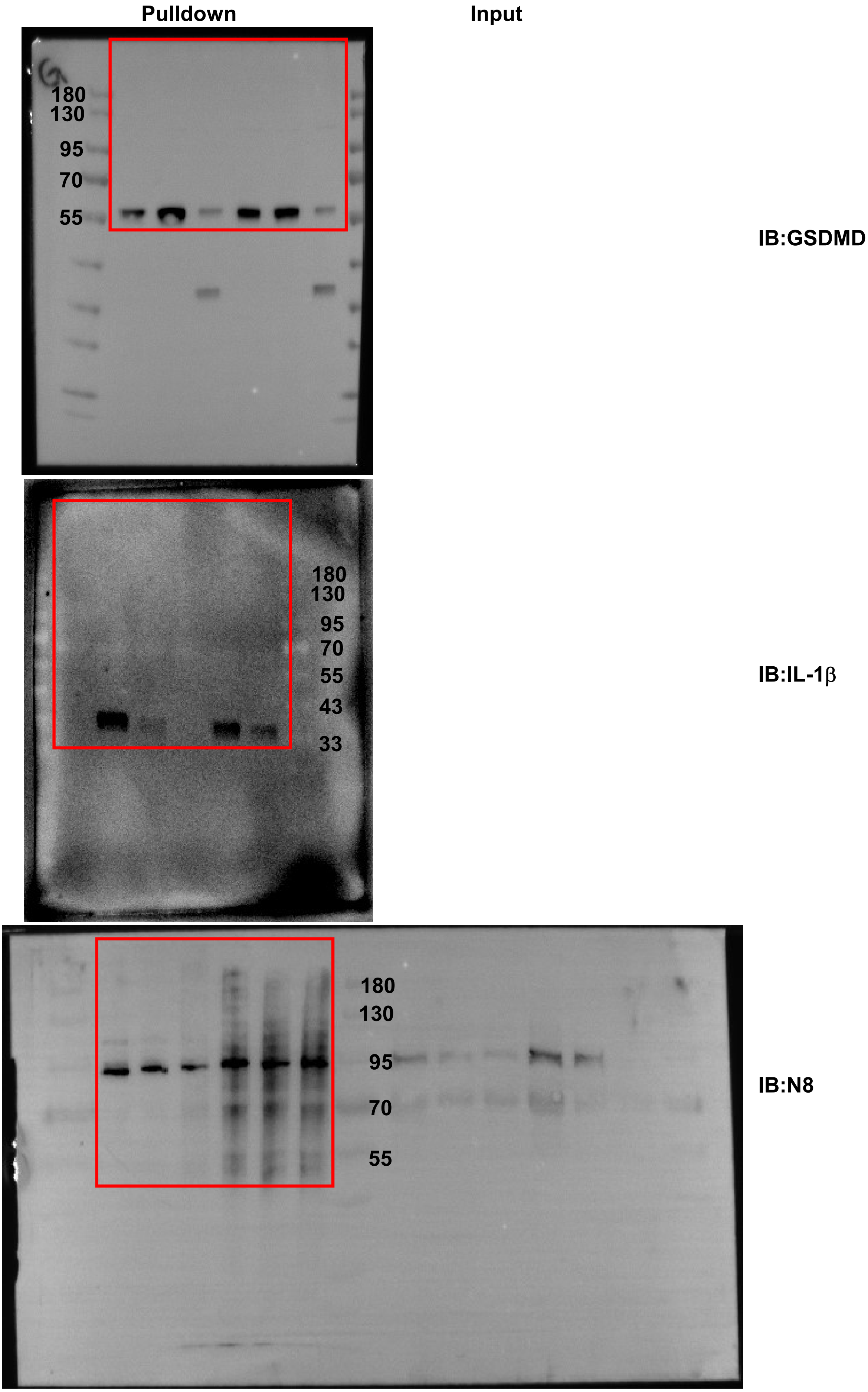

Source Figure S7a

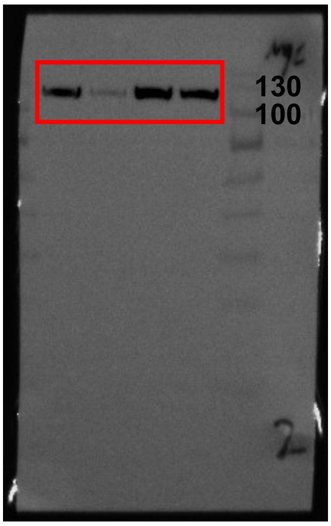

IB:Myc

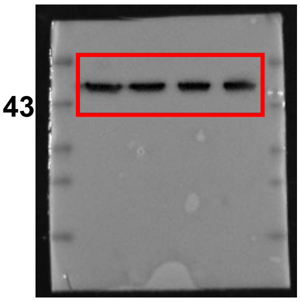

IB:β-actin

Source Figure S7b

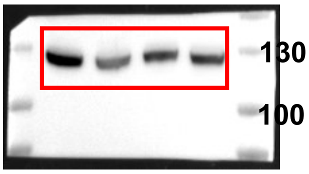

IB:Myc

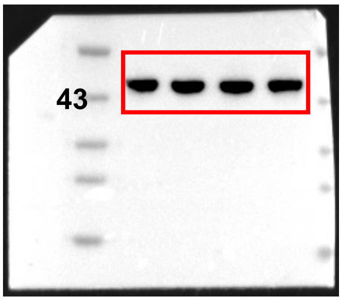

IB:β-actin

Source Figure S8

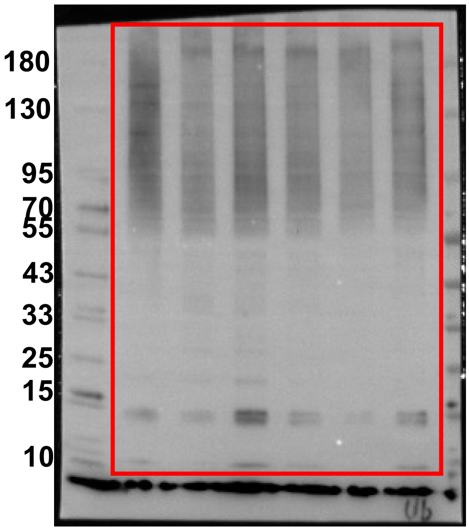

IB:Ub

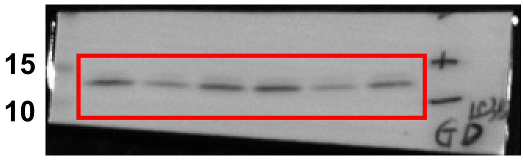

IB:LC3B

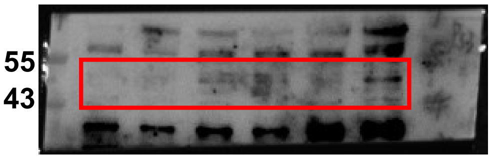

IB:p53

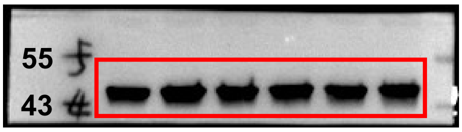

IB:β-actin

Source Figure S9

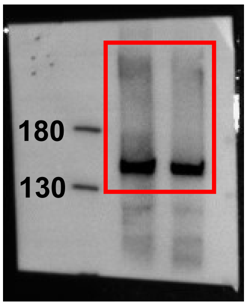

IB:GST

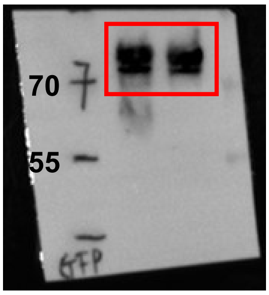

IB:GFP
